# High-Dose Aumolertinib for Untreated *EGFR*-Variant Non–Small Cell Lung Cancer With Brain Metastases: The ACHIEVE Phase 2 Nonrandomized Clinical Trial

**DOI:** 10.1001/jamaoncol.2025.1779

**Published:** 2025-06-26

**Authors:** Hui Li, Kaiyan Chen, Lei Gong, Jing Qin, Ying Jin, Rongrong Zhou, Zhiyu Huang, Yanjun Xu, Xiaoling Xu, Jingdong He, Junfei Zhu, Sizhe Yu, Hongyang Lu, Yujin Xu, Xinmin Yu, Guang Han, Jun Chen, Wei Tan, Guangyuan Lou, Biyong Ren, Xiangqi Chen, Dianbao Zhang, Wenxian Wang, Xun Shi, Fajun Xie, Jun Zhao, Na Han, Bing Li, Yun Fan

**Affiliations:** 1 Department of Thoracic Medical Oncology, Zhejiang Cancer Hospital, Hangzhou Institute of Medicine (HIM), Chinese Academy of Sciences, Hangzhou, China; 2 Department of Radiation Oncology, Xiangya Hospital, Central South University, Changsha, China; 3 Department of Medical Oncology, Huai’an First People’s Hospital, Huai’an, China; 4 Department of Respiratory Medicine, Taizhou Central Hospital, Taizhou, China; 5 Department of Thoracic Radiotherapy, Zhejiang Cancer Hospital, Hangzhou Institute of Medicine (HIM), Chinese Academy of Sciences, Hangzhou, China; 6 Department of Radiation Oncology, Hubei Cancer Hospital, Tongji Medical College, Huazhong University of Science and Technology, Wuhan, China; 7 Department of Chemoradiotherapy, The Affiliated People’s Hospital of Ningbo University, Ningbo, China; 8 Department of Respiratory Medicine, Weifang People’s Hospital, Weifang, China; 9 Department of Oncology, Chongqing University Three Gorges Hospital, Chongqing, China; 10 Department of Pulmonary and Critical Care Medicine, Fujian Medical University Union Hospital, Fuzhou, China; 11 Department of Medical Oncology, The First Affiliated Hospital of Henan University of Science and Technology, Luoyang, China; 12 Burning Rock Biotech, Guangzhou, China

## Abstract

**Importance:**

Central nervous system (CNS) metastases remain a significant challenge in the management of *EGFR*-variant non–small cell lung cancer (NSCLC).

**Objective:**

To evaluate the activity and safety of high-dose aumolertinib in patients with untreated *EGFR*-variant NSCLC and brain metastases.

**Design, Setting, and Participants:**

This was a phase 2 nonrandomized clinical trial conducted at 10 centers in China. Patients with untreated *EGFR*-variant metastatic NSCLC and brain metastases were enrolled between July 6, 2021, and August 31, 2022. The data cutoff date was October 10, 2024.

**Interventions:**

Patients received aumolertinib, 165 mg, orally once daily until disease progression or unacceptable toxic effects.

**Main Outcomes and Measures:**

The primary end point was 12-month progression-free survival (PFS) rate assessed by investigators according to the Response Evaluation Criteria In Solid Tumors (RECIST), version 1.1.

**Results:**

A total of 63 patients (39 female [61.9%]; median age, 60 [range, 47-76] years) were enrolled (full analysis set), and 49 had at least 1 measurable brain lesion (CNS evaluable-for-response set). Median follow-up duration was 28.8 months (95% CI, 27.0-29.8). In the full analysis set, the 12-month PFS rate was 62.1% (95% CI, 48.7-73.0), the median PFS was 20.5 months (95% CI, 12.0-26.9), the 12-month intracranial PFS rate was 76.8% (95% CI, 63.2-85.9), and the median intracranial PFS and overall survival were not reached. Systemic and intracranial objective response rates per RECIST 1.1 were 56 of 63 (88.9% [95% CI, 78.4-95.4]) and 52 of 63 (82.5% [95% CI, 70.9-90.9]) in the full analysis set and 43 of 49 (87.8% [95% CI, 75.2-95.4]) and 42 of 49 (85.7% [95% CI, 72.8-94.1]) in the CNS evaluable-for-response set, respectively. The most common grade 3 or 4 treatment-related adverse event was increased blood creatine phosphokinase (17 participants [27.0%]). No treatment-related deaths occurred. *EGFR* variant clearance in plasma circulating tumor DNA at day 1 of cycle 2 was independently associated with longer PFS (hazard ratio, 0.14 [95% CI, 0.04-0.47]; *P* = .001).

**Conclusions and Relevance:**

The findings of this nonrandomized clinical trial suggest that high-dose aumolertinib is associated with long-term survival benefit in patients with untreated *EGFR*-variant NSCLC and brain metastases, with a manageable safety profile.

**Trial Registration:**

ClinicalTrials.gov Identifier: NCT04808752

## Key Points

**Question:**

What is activity of treatment with high-dose third-generation epidermal growth factor receptor (EGFR) tyrosine kinase inhibitor in patients with untreated *EGFR*-variant non–small cell lung cancer (NSCLC) and brain metastases?

**Findings:**

In this multicenter, phase 2 nonrandomized clinical trial of 63 patients treated with high-dose aumolertinib, the 12-month progression-free survival rate was 62.1%. The safety profile was manageable.

**Meaning:**

The findings of this study suggest that high-dose aumolertinib is associated with long-term survival benefit in patients with untreated *EGFR*-variant NSCLC and brain metastases, which warrants further validation in a randomized clinical trial.

## Teaser

This nonrandomized clinical trial evaluates the activity and safety of high-dose aumolertinib in patients with untreated *EGFR*-variant non–small cell lung cancer and brain metastases.

## Introduction

*EGFR* variants are common gene alterations in lung adenocarcinoma, with an incidence of 10% to 50% based on different races.^1^ The central nervous system (CNS) is one of the most common metastatic sites in patients with *EGFR*-variant non–small cell lung cancer (NSCLC). CNS metastases can be detected in 25% to 40% of this population at diagnosis, with the incidence increasing to 50% over the course of the disease.^2,3^ Once CNS metastases appear, they seriously affect patient survival and quality of life.

Epidermal growth factor receptor (EGFR) tyrosine kinase inhibitors (TKIs) have consistently shown superior efficacy and safety compared to platinum-based chemotherapy in multiple phase 3 trials,^4,5,6,7,8,9,10^ establishing the standard first-line treatment for patients with *EGFR*-variant advanced NSCLC. Especially due to increased CNS permeability and superior survival data compared with first-generation TKIs,^11,12^ third-generation EGFR TKIs have been recommended as the preferred first-line treatment option for this population.^10^ Aumolertinib is a third-generation, irreversible EGFR TKI. It has been approved for the first-line treatment of patients with *EGFR*-variant advanced NSCLC in China based on the phase 3 AENEAS study.^13^ The subgroup analysis in patients with baseline brain metastases also showed better intracranial objective response rate (ORR; 62.7% vs 49.1%) and median intracranial progression-free survival (PFS; 29.0 vs 8.3 months; hazard ratio [HR], 0.31 [95% CI, 0.17-0.56]) with aumolertinib compared to gefitinib.^14^

Despite promising CNS efficacy with third-generation EGFR TKIs, patients with brain metastases still have poorer survival compared to those without.^10,13^ More effective regimens are still needed. A phase 2 study showed that osimertinib, 160 mg, could result in a promising intracranial ORR of 55.0% and a median PFS of 7.6 months in patients with *EGFR* T790M–positive NSCLC and brain metastases who progressed with prior EGFR TKI therapy,^15^ suggesting the potential of high-dose strategy. Likewise, a pharmacokinetic study of aumolertinib has shown that escalating the dosage from 55 mg to 220 mg enhances drug exposure while maintaining an acceptable safety profile.^16^

Liquid biopsy analysis of plasma circulating tumor DNA (ctDNA) has shown valuable insight in monitoring treatment efficacy during systemic therapy in advanced NSCLC. Baseline ctDNA negativity and early clearance or reduction of ctDNA after treatment are associated with prolonged survival.^17,18^ However, the sensitivity and prediction performance in NSCLC with brain metastases are controversial.^19,20,21^

Therefore, we conducted this study to investigate high-dose aumolertinib as first-line treatment in patients with untreated *EGFR*-variant NSCLC and brain metastases. Potential biomarkers associated with efficacy were also explored.

## Methods

### Study Design and Participants

ACHIEVE was a multicenter, single-arm, nonrandomized phase 2 trial conducted across 10 centers in China. This study was conducted in accordance with the Declaration of Helsinki and Good Clinical Practice. The study protocol was approved by the ethics committee of each participating center. All patients provided written informed consent before enrollment. This trial followed the Transparent Reporting of Evaluations With Nonrandomized Designs (TREND) reporting guideline.

Patients could be included if they were 18 years and older; had pathologically confirmed metastatic NSCLC; had *EGFR* exon 19 deletion or 21 L858R variant, detected by certified local laboratories using next-generation sequencing or real-time polymerase chain reaction from either tumor or plasma samples; had brain metastases without neurological symptoms or with mild to moderate symptoms that could be controlled by steroids or mannitol; had an Eastern Cooperative Oncology Group performance status of 0 or 1; had at least 1 measurable extracranial target lesion and at least 1 intracranial lesion with the longest diameter of at least 5 mm; had no previous systemic therapy for metastatic NSCLC or local radiotherapy for brain lesions; had a life expectancy of 3 months or more; and had adequate organ function. The key exclusion criteria were leptomeningeal metastases, mixed small cell lung cancer and NSCLC, large cell neuroendocrine carcinoma of the lung, or lung sarcomatoid carcinoma. Patients were enrolled between July 6, 2021, and August 31, 2022. The data cutoff date was October 10, 2024. Full eligibility criteria are listed in the study protocol (Supplement 1).

### Procedures

Eligible patients received aumolertinib, 165 mg, orally once daily in each 28-day cycle until disease progression, unacceptable toxic effects, withdrawal of consent, or death. Continuation of treatment after disease progression was allowed if the investigators judged that patients could still benefit from aumolertinib. Dose reductions or interruptions were permitted to manage adverse events (AEs).

Brain magnetic resonance imaging and body computed tomography scans were performed at baseline and every 8 weeks. Baseline magnetic resonance imaging images were centrally reviewed by an independent radiologist. Intracranial and systemic responses were assessed by investigators per the Response Evaluation Criteria In Solid Tumors (RECIST), version 1.1. Survival follow-up was conducted every 12 weeks. AEs were monitored and graded per the National Cancer Institute Common Terminology Criteria for Adverse Events, version 5.0.

### End Points

The primary end point was 12-month PFS rate. PFS was defined as time from treatment initiation to disease progression at any site or death from any cause.

Secondary end points were intracranial PFS (defined as time from treatment initiation to intracranial progression or death from any cause), systemic objective response rate (defined as the proportion of patients with a confirmed systemic complete response [CR] or partial response [PR]), intracranial ORR (defined as the proportion of patients with a confirmed intracranial CR or PR), duration of systemic response (defined as the time from the first documented systemic CR or PR to disease progression at any site or death from any cause), duration of intracranial response (defined as the time from the first documented intracranial CR or PR to intracranial progression or death from any cause), systemic disease control rate (defined as the proportion of patients with a confirmed systemic CR, PR, or stable disease), intracranial disease control rate (defined as the proportion of patients with a confirmed intracranial CR, PR, or stable disease), overall survival (OS; defined as time from treatment initiation to death from any cause), and safety. The exploratory end points were biomarkers associated with antitumor activity.

### Cell-Free DNA Sequencing Analysis

Cell-free DNA (cfDNA) isolation, library construction, and sequencing were performed according to standardized protocols.^22^ The cfDNA extracted from plasma was purified using the QIAamp Circulating Nucleic Acid Kit (Qiagen). DNA yield was quantified with the Qubit dsDNA High Sensitivity Assay Kit and Qubit 2.0 Fluorometer (Life Technologies). The OncoScreen Plus gene panel targeting 520 cancer genes across 1.86 megabases was used for cfDNA hybridization and capture. Indexed samples were sequenced on the NovaSeq 6000 Platform (Illumina) using 2 × 150 base pair reads, achieving a mean depth of 16 022 × (9797 − 24071). To exclude clonal hematopoiesis variants, sequencing of matched white blood cells was performed. Bioinformatics analysis postsequencing used sophisticated pipelines for the identification and characterization of a broad range of cancer-associated somatic variants. The limit of detection was 0.05% cfDNA content.

### Statistical Analysis

We assumed that the null hypothesis of 12-month PFS rate was 40%, and the alternative hypothesis was 60%. With a 2-sided significance level of .10 and a power of 80%, 49 patients were required. Considering a dropout rate of 20%, the study needed to enroll 63 patients.

Patients who received at least 1 dose of aumolertinib were included in the full analysis set and safety set, of whom patients with at least 1 measurable brain lesion were included in the CNS evaluable-for-response set. Survival was mainly analyzed in the full analysis set. Baseline characteristics, tumor response, and duration of response were analyzed in both the full analysis set and CNS evaluable-for-response set. Safety was analyzed in the safety set. Survival and duration of response were estimated using the Kaplan-Meier method, and the corresponding CIs were estimated using the Brookmeyer-Crowley method. Follow-up duration was estimated using the reverse Kaplan-Meier approach. The 95% CIs of ORR and disease control rate were estimated using the Clopper-Pearson method.

A post hoc assessment of intracranial response was conducted by investigators according to the Response Assessment in Neuro-Oncology-Brain Metastases (RANO-BM) criteria, with prospectively collected data. Post hoc analyses on the potential associations of *EGFR* variant clearance in plasma ctDNA after 1 cycle of treatment (day 1 of cycle 2) and baseline characteristics with PFS were performed using the univariate and multivariate Cox regression models. The Benjamini–Hochberg method was used to control the false discovery rate for multiple testing. Independent variables were included in the multivariate model using the backward selection method with a significance level of .50. The proportional hazards assumption in the univariate and multivariate models was tested by examining Schoenfeld residuals, and time-varying covariates would be included when the Schoenfeld residual plots suggested violation of proportional hazards assumption. Statistical analysis was performed using R, version 4.3.2 (R Project for Statistical Computing), and SAS, version 9.4 (SAS Institute). Two-sided *P* < .05 was considered statistically significant.

## Results

### Participant Characteristics

**Figure 1.**
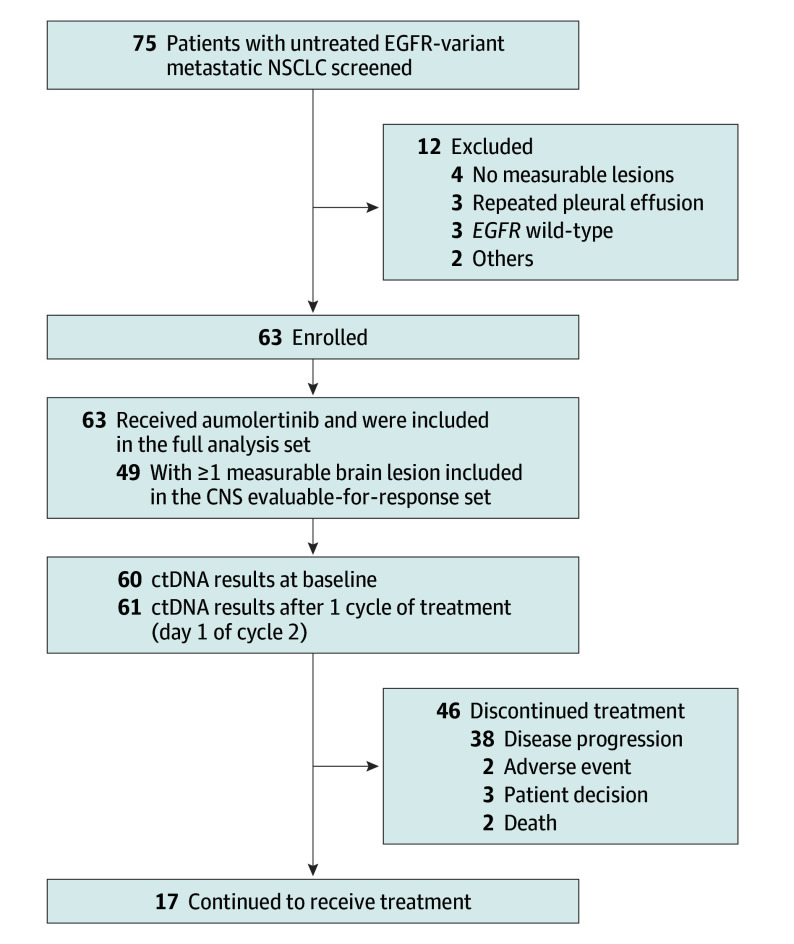
Patient Flowchart CNS indicates central nervous system; ctDNA, circulating tumor DNA; NSCLC, non–small cell lung cancer.

Seventy-five patients were screened, of whom 63 eligible patients were enrolled in the full analysis set and 49 patients in the CNS evaluable-for-response set (Figure 1). Baseline characteristics are summarized in Table 1. Of 63 patients, the median age was 60 years (range, 47-76), and 39 (61.9%) were female. Thirty-three patients (52.4%) had *EGFR* exon 19 deletion, and 30 (47.6%) had exon 21 L858R variant. Twenty-nine patients (46.0%) harbored *TP53* covariants. The distant metastatic lesions were located only in the brain in 21 patients (33.3%), and 42 patients (66.7%) had simultaneous extracranial metastases. Twelve patients (19.0%) had symptomatic brain metastases at baseline.

**Table 1.** Baseline Characteristics

Characteristic	No. (%)	
	Full analysis set (n = 63)	CNS evaluable-for-response set (n = 49)
Age, median (range), y	60 (47-76)	59 (47-76)
Sex		
Female	39 (61.9)	33 (67.3)
Male	24 (38.1)	16 (32.7)
ECOG performance status		
0	17 (27.0)	14 (28.6)
1	46 (73.0)	35 (71.4)
Smoking status		
Never smoker	46 (73.0)	37 (75.5)
Current or former smoker	17 (27.0)	12 (24.5)
Histologic type		
Adenocarcinoma	63 (100)	49 (100)
Clinical stage		
IVA	19 (30.2)	14 (28.6)
IVB	44 (69.8)	35 (71.4)
*EGFR* variant type		
Exon 19 deletion	33 (52.4)	23 (46.9)
Exon 21 L858R	30 (47.6)	26 (53.1)
*TP53* covariant^a^		
Yes	29 (46.0)	22 (44.9)
No	16 (25.4)	11 (22.4)
Unknown	18 (28.6)	16 (32.7)
Symptoms of brain metastases		
No	51 (81.0)	37 (75.5)
Yes	12 (19.0)	12 (24.5)
Extracranial metastases		
No	21 (33.3)	16 (32.7)
Yes	42 (66.7)	33 (67.3)
No. of brain metastases		
1-3	28 (44.4)	19 (38.8)
>3	35 (55.6)	30 (61.2)
Sum of intracranial target lesion diameters, median (range), mm	NA	23.6 (10.0-70.4)

Abbreviations: CNS, central nervous system; ECOG, Eastern Cooperative Oncology Group; NA, not applicable.

^a^ Based on circulating tumor DNA detection results using baseline plasma samples.

### Antitumor Activity

**Figure 2.**
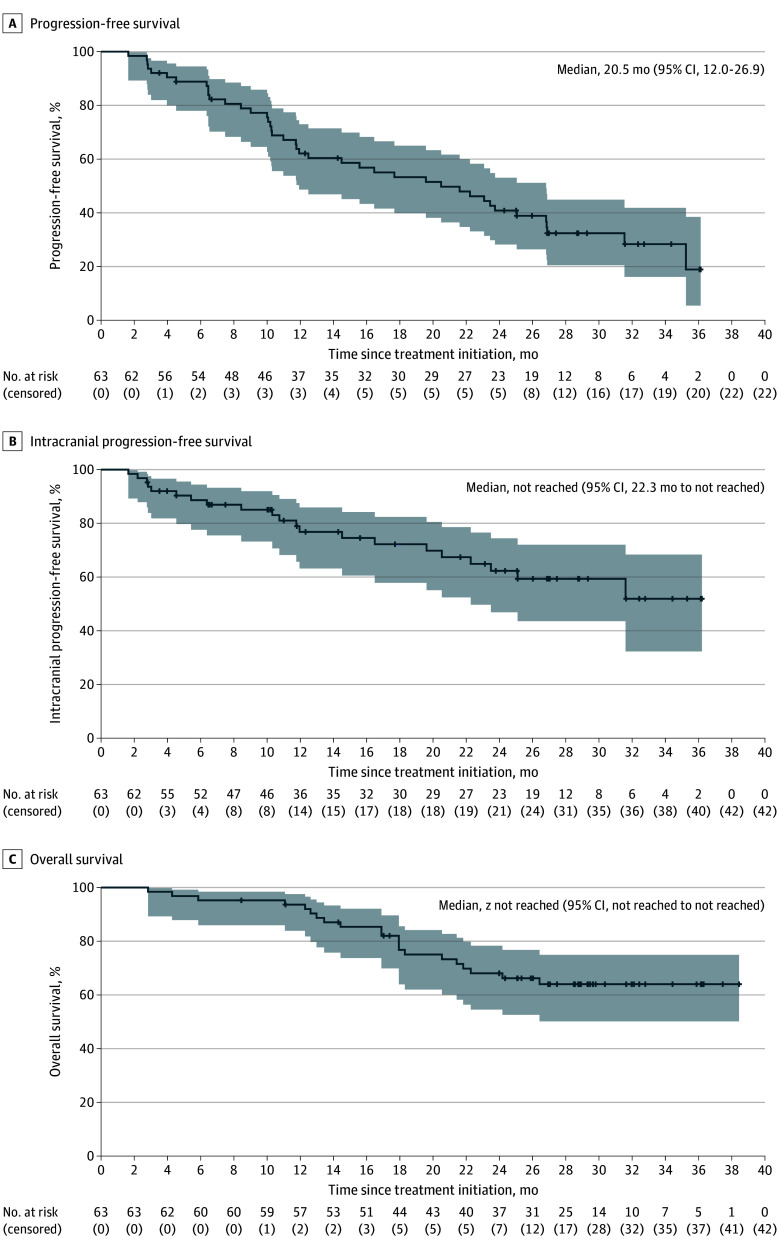
Kaplan-Meier Estimates of Survival in the Full Analysis Set The shaded areas represent 95% CIs.

By the data cutoff date, the median follow-up duration was 28.8 months (95% CI, 27.0-29.8). A total of 41 of 63 patients (65.1%) in the full analysis set experienced PFS events. The 12-month PFS rate was 62.1% (95% CI, 48.7-73.0), the median PFS was 20.5 months (95% CI, 12.0-26.9), and the 24-month PFS rate was 40.8% (95% CI, 28.2-53.1; Figure 2A). Median intracranial PFS was not reached (NR; 95% CI, 22.3 months to NR), with a data maturity of 33.3% (21 events). The intracranial PFS rates at 12 and 24 months were 76.8% (95% CI, 63.2-85.9) and 62.3% (95% CI, 46.9-74.4; Figure 2B), respectively. Of 18 patients with intracranial progression, 4 also had simultaneous extracranial progression; 15 patients had progression at brain parenchymal lesions (including 2 patients with simultaneous new leptomeningeal lesions), and 3 had progression due to leptomeningeal metastases. Twenty patients (31.7%) died, and the median OS was NR (95% CI, NR-NR). The estimated OS rate was 93.6% at 12 months and 68.1% at 24 months (Figure 2C).

**Figure 3.**
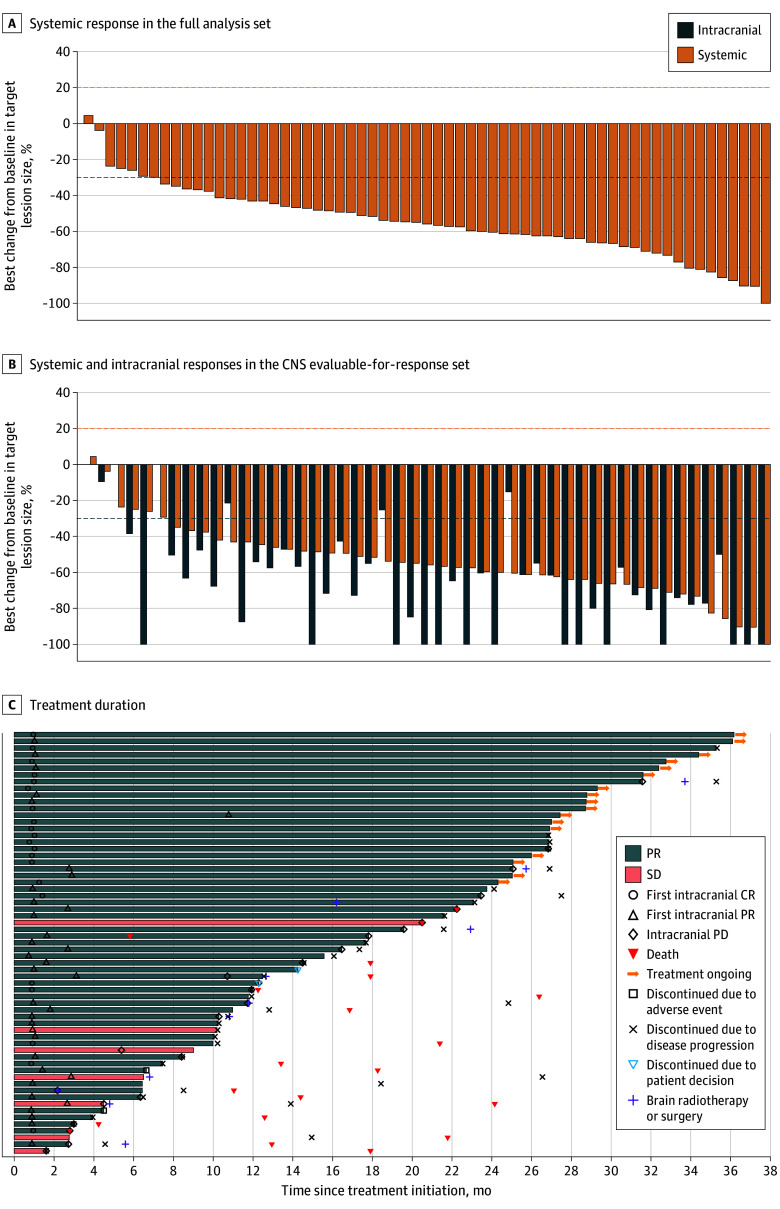
Tumor Response per Response Evaluation Criteria in Solid Tumors (RECIST), Version 1.1, and Treatment Duration A, Waterfall plot for systemic response in the full analysis set. B, Waterfall plot for systemic and intracranial responses in the central nervous system (CNS) evaluable-for-response set. C, Swimmer plot for treatment duration in the full analysis set. Dashed lines at 20% and −30% in panels A and B denote thresholds for progressive disease (PD) and partial response (PR), respectively, according to RECIST 1.1. CR indicates complete response; SD, stable disease.

In the full analysis set, the systemic ORR was 88.9% (95% CI, 78.4-95.4) and the intracranial ORR was 82.5% (95% CI, 70.9-90.9) per RECIST 1.1, with 21 intracranial CR events observed (33.3%). Both the systemic and intracranial disease control rates were 100% (95% CI, 94.3-100; Figure 3A). In the CNS evaluable-for-response set, the systemic ORR was 87.8% (95% CI, 75.2-95.4) and the intracranial ORR was 85.7% (95% CI, 72.8-94.1) per RECIST 1.1, with 11 intracranial CR events (22.4%) observed (Figure 3B). A high consistency in intracranial ORR was observed between RANO-BM criteria and RECIST 1.1 (Table 2). Median best percentage change from baseline in target lesion size was −55.9% (range, −100% to 4.4%) for systemic lesions and −67.7% (range, −100% to 0%) for intracranial lesions in the CNS evaluable-for-response set. Of 49 patients in the CNS evaluable-for-response set, 40 (81.6%) had both systemic and intracranial responses. Median duration of systemic response was 21.4 months (95% CI, 12.9-26.2) in the full analysis set and 20.7 months (95% CI, 9.4-25.9) in the CNS evaluable-for-response set (Figure 3C). Median duration of intracranial response was 26.2 months (95% CI, 16.8 months to NR) per RECIST 1.1 and 30.6 months (95% CI, 18.7 months to NR) per RANO-BM criteria in the CNS evaluable-for-response set (Table 2).

**Table 2.** Systemic and Intracranial Response

Response	Full analysis set (n = 63)			CNS evaluable-for-response set (n = 49)		
	Response by RECIST 1.1		Intracranial response by RANO-BM criteria	Response by RECIST 1.1		Intracranial response by RANO-BM criteria
	Systemic	Intracranial		Systemic	Intracranial	
Best response, No. (%)						
CR	0	21 (33.3)	21 (33.3)	0	11 (22.4)	11 (22.4)
PR	56 (88.9)	31 (49.2)	32 (50.8)	43 (87.8)	31 (63.3)	32 (65.3)
SD^a^	7 (11.1)	11 (17.5)	10 (15.9)	6 (12.2)	7 (14.3)	6 (12.2)
PD	0	0	0	0	0	0
ORR (95% CI), %	88.9 (78.4-95.4)	82.5 (70.9-90.9)	84.1 (72.7-92.1)	87.8 (75.2-95.4)	85.7 (72.8-94.1)	87.8 (75.2-95.4)
DCR (95% CI), %	100 (94.3-100)	100 (94.3-100)	100 (94.3-100)	100 (92.7-100)	100 (92.7-100)	100 (92.7-100)
Median DoR (95% CI), mo	21.4 (12.9-26.2)	NR (22.1-NR)	NR (19.6-NR)	20.7 (19.4-25.9)	26.2 (16.8-NR)	30.6 (18.7-NR)

Abbreviations: CNS, central nervous system; CR, complete response; DCR, disease control rate; DoR, duration of response; NR, not reached; ORR, objective response rate; PD, progressive disease; PR, partial response; RANO-BM, Response Assessment in Neuro-Oncology Brain Metastases; RECIST, Response Evaluation Criteria In Solid Tumors; SD, stable disease.

^a^ For patients with nontarget lesions only, they were categorized as SD if they did not achieve CR or PD.

### Safety

Treatment-related AEs (TRAEs) of any grade were reported in 58 of 63 patients (92.1%), with grade 3 or higher TRAEs occurring in 20 patients (31.7%). The most common TRAEs were increased blood creatine phosphokinase (43 [68.3%]), increased aspartate aminotransferase (31 [49.2%]), increased alanine aminotransferase (24 [38.1%]), and increased blood lactate dehydrogenase (17 [27.0%]). Grade 3 or higher TRAEs included increased blood creatine phosphokinase (17 [27.0%]) and increased alanine aminotransferase (2 [3.2%]; eTable 1 in Supplement 2). TRAEs led to dose reduction of aumolertinib in 7 patients (11.1%). Two patients (3.2%) discontinued aumolertinib treatment owing to TRAEs (grade 2 pneumonia for both). No treatment-related deaths occurred.

### Biomarkers

Sixty plasma samples at baseline and 61 plasma samples at day 1 of cycle 2 were collected to analyze variant profiles of ctDNA as well as the dynamic patterns. Baseline variant profiles are shown in eFigure 1 in Supplement 2. *EGFR* variants in baseline plasma ctDNA were detected in 45 of 60 patients (75.0%). The frequently coaltered genes included *TP53*, *RB1*, *KMT2C*, and *RBM10*. *EGFR* variants in baseline plasma ctDNA were not associated with PFS (eTable 2 in Supplement 2). At day 1 of cycle 2, the *EGFR* variant clearance rate reached 35 of 45 (77.8%). The univariate analysis showed that *EGFR* variant clearance at day 1 of cycle 2 was associated with longer PFS (median, 23.8 months [95% CI, 17.7-35.3] vs 7.5 months [95% CI, 2.8-14.5]; HR, 0.17 [95% CI, 0.07-0.42]; false discovery rate–adjusted *P* = .002; eTable 2 and eFigure 2 in Supplement 2). Results of backward selection and Schoenfeld residual plots are shown in eTable 3, eFigure 3, and eFigure 4 in Supplement 2. The multivariate analysis further demonstrated the association between *EGFR* variant clearance at day 1 of cycle 2 and PFS (HR, 0.14 [95% CI, 0.04-0.47]; *P* = .001; eTable 4 in Supplement 2).

## Discussion

To our knowledge, ACHIEVE was the first prospective study specifically designed to assess the activity and safety of high-dose aumolertinib in patients with untreated *EGFR*-variant metastatic NSCLC and brain metastases. The primary end point, 12-month PFS rate, was 62.1%, and the median PFS was 20.5 months. The safety profile was manageable. The results of this nonrandomized clinical trial suggest the potential of high-dose aumolertinib as first-line treatment for this population.

In our study, the median PFS for high-dose aumolertinib, 165 mg, was longer than that in the brain metastases subgroup with standard-dose osimertinib, 80 mg, over 15.2 months from FLAURA and standard-dose aumolertinib, 110 mg, over 15.3 months from AENEAS.^10,13^ The 18-month intracranial PFS rate (72.2%) was also higher than that in these 2 previous studies (58%-60%).^12,14^ Notably, compared with the brain metastases subpopulation in FLAURA, patients in our study had heavier intracranial tumor burden (≥10 mm target lesions: 36.1% vs 77.8% [full analysis set]; >3 brain lesions: 23.0% vs 55.6% [full analysis set]; target lesion size: 16 mm vs 23.6 mm [CNS evaluable-for-response set]). Our study also allowed for enrollment of patients with neurological symptoms (19%), while only asymptomatic or stable brain metastases were included in FLAURA and AENEAS.^10,13^ Furthermore, the single-agent high-dose aumolertinib showed comparable median PFS to the combination therapy in FLAURA2 (24.9 months for osimertinib plus platinum-based chemotherapy) and MARIPOSA (18.3 months for amivantamab plus lazertinib),^23,24^ and the enrollment criteria and baseline characteristics for the brain metastases subgroup in these 2 studies are similar to those in FLAURA.^10,23,24,25^ Although these indirect comparisons might support the treatment potential of high-dose aumolertinib in patients with untreated *EGFR*-variant NSCLC and brain metastases, the results should be interpreted with caution, considering the single-arm design of our study and different clinical settings across studies. On the other hand, despite the approval of the abovementioned combination regimens by the US Food and Drug Administration for the treatment of untreated *EGFR*-variant advanced NSCLC,^23,24^ monotherapy with third-generation EGFR TKI may still be a mainstay treatment in clinical practice,^26^ especially for patients who are intolerant to or refuse combination therapy.

The systemic ORR was 88.9% in the full analysis set, higher than that with standard-dose osimertinib (80%) and aumolertinib (73.8%).^10,13^ The intracranial ORR was 85.7% per RECIST 1.1 and 87.8% per RANO-BM criteria in the CNS evaluable-for-response set, and 81.6% of patients had both systemic and intracranial responses. Despite that our study had more patients with more than 3 brain lesions and larger lesion size compared to FLAURA, the intracranial CR rate (CNS evaluable-for-response set, 22.4% vs 23%) was comparable between studies.^12^ These findings suggest that high-dose aumolertinib is associated with good intracranial and extracranial disease control.

The AEs of high-dose aumolertinib reported in our study are consistent with the toxic effect profile in AENEAS.^13^ Most TRAEs (including hematological toxic effects and hepatotoxic effects) were grade 1 or 2. The major concern for grade 3 or higher TRAEs was increased blood creatine phosphokinase, which occurred in 17 patients (27.0%) and could be relieved by treatment interruption or dose adjustment. No new safety signals were identified.

Most patients (75%) had detectable *EGFR* variants in plasma ctDNA at baseline, consistent with FLAURA (70.5%) and FLAURA2 (73.2%).^27,28^ We did not find an association between baseline *EGFR* variant status in ctDNA and PFS. Baseline ctDNA positivity indicates a higher tumor burden and is associated with poorer PFS and OS.^17,27^ Both the FLAURA2 and MARIPOSA studies demonstrated that the combination therapy could significantly improve PFS in patients with baseline positive ctDNA, while there was no significant difference in PFS for patients with negative ctDNA.^28,29^ Therefore, combination therapy may reduce the difference in PFS between ctDNA-positive and ctDNA-negative patients compared to monotherapy. Similarly, the use of high-dose aumolertinib treatment indicates the same phenomenon. After treatment, 77.8% of patients with baseline *EGFR* variants in plasma ctDNA achieved clearance at day 1 of cycle 2 in our study, which is similar to the osimertinib group in FLAURA2 (71% at week 3 and 89% at week 6).^28^ Consistently, *EGFR* variant clearance at day 1 of cycle 2 in our study was associated with longer PFS, suggesting the value of liquid biopsy during treatment. This may help us target the population more precisely for those who would benefit from TKI therapy alone. It also deserves consideration whether the change in treatment regimen is necessary for patients without early *EGFR* variant clearance. Two phase 2 trials^30,31^ are ongoing to compare the clinical outcomes with third-generation EGFR TKI plus chemotherapy vs EGFR TKI alone in this population. Given the small sample size, these exploratory findings still need further validation.

### Limitations

This study had limitations. First, this was a single-arm study without a control group, leading to potential selection bias. Second, tumor response was assessed by investigators without independent central review, which might introduce potential for assessment bias. Third, the study only enrolled Chinese patients, and the results need to be validated in other populations. Fourth, the relatively small sample size in subgroups diminishes the statistical power to draw any conclusions. Finally, intracranial PFS and OS are not mature yet, and extended follow-up is ongoing. Long-term survival results will be reported in the future.

## Conclusions

The findings of this nonrandomized clinical trial suggest that high-dose aumolertinib treatment is associated with long survival benefit in patients with untreated *EGFR*-variant metastatic NSCLC and brain metastases, with a manageable safety profile. It deserves further validation in a randomized clinical trial.

## Notes

Supplement 1. Trial Protocol

Supplement 2. eTable 1. Treatment-related adverse events eTable 2. Post-hoc univariate analysis of progression-free survival eTable 3. Procedure of backward selection in multivariate Cox regression model eTable 4. Post-hoc multivariate Cox regression analysis of progression-free survival among patients with *EGFR* variants in baseline plasma ctDNA eFigure 1. Genomic landscape in patients with available baseline plasma samples for circulating tumor DNA sequencing eFigure 2. Association between *EGFR* variant clearance in plasma circulating tumor DNA at day 1 of cycle 2 and progression-free survival among patients with *EGFR* variants in baseline plasma circulating tumor DNA eFigure 3. Schoenfeld residual scaled plots in multiple univariate Cox regression models eFigure 4. Schoenfeld residual scaled plots in multivariate Cox regression model

Supplement 3. Data Sharing Statement
