# Supplementary material for: High-Dose Aumolertinib for Untreated EGFR-Variant Non–Small Cell Lung Cancer With Brain Metastases: The ACHIEVE Phase 2 Nonrandomized Clinical Trial
Source: JAMA Oncol. 2025 Jun 26;11(8):900–8. doi: 10.1001/jamaoncol.2025.1779 (PMC12203398; doi:10.1001/jamaoncol.2025.1779)
Supplement: Supplement 1. — Trial Protocol [file jamaoncol-e251779-s001.pdf]

Clinical Study Protocol No.: YX-L-202107

High-Dose Aumolertinib as First-Line Treatment in Patients with EGFR-Sensitive Mutations in Advanced NSCLC with Brain Metastases: Prospective, Open-Label, Multi-center, Single-Arm Clinical Trial

(ACHIEVE)

Principal investigator: Yun Fan

Sponsor: Cancer Hospital of the University of Chinese Academy of Sciences (Zhejiang Cancer Hospital)

Address: No. 38 Guangji Road, Banshan Bridge, Gongshu District, Hangzhou, Zhejiang, China

Version No.: 02

Version Date: Jul. 28, 2021

1.0 VERSION HISTORY/AMENDMENT HISTORY

Version	Version date	Summary of major changes
01	January 20, 2021	Not applicable
02	July 28, 2021	1. Sample size calculation was performed based on the latest parameters. The number of subjects required to be enrolled was 63.<br>2. The primary endpoint, progression-free survival (PFS), is clearly defined as the 12-month PFS rate.<br>3. The inclusion criterion for subject's baseline intracranial lesion size was updated to ≥5 mm (previously ≥10 mm) following a review of large-scale clinical research literatures.

TABLE OF CONTENTS

1.0 VERSION HISTORY/AMENDMENT HISTORY 2

TABLE OF CONTENTS 3

2.0 STUDY SYNOPSIS 8

3.0 LIST OF ABBREVIATIONS 12

4.0 STUDY BACKGROUND AND SCIENTIFIC RATIONALE 14

4.1 Study Background 14
4.2 Scientific Rationale 14
4.2.1 Mechanism of action and pharmacology 15
4.2.2 Nonclinical pharmacokinetics (PK) and drug metabolism 15
4.2.3 Nonclinical toxicology 15
4.2.4 Clinical studies 16
4.3 Potential Risks and Benefits 17
4.3.1 Potential risks and control plan 17
For the above adverse reactions, the following provisions are made in the protocol to minimize the risk: 1) The safety observation indicators are designed to cover the common adverse reactions of competitors, the changes requiring close attention suggested in the completed preclinical and clinical studies of aumolertinib, as well as the observation of toxicities to the heart, lung, and other vital organs. The termination criteria for subjects and the clinical trial are specified in the protocol. 2) The trial will be conducted in medical institutions with clinical trial qualifications, and experienced investigators will be selected for the clinical trial. 3) During the conduct and implementation of the trial, physicians with rich clinical experience shall regularly pay attention to and examine the health status of the patients and take corresponding treatment measures in time in case of abnormal changes. 4) The safety examinations are completed at the time points defined in the protocol, and AEs occurring in patients outside the hospital are monitored by subject diary, telephone, or in-hospital follow-ups. 5) Additional tests may be performed at any time as deemed necessary by the investigator. In case of any special event after the trial is initiated, if the ethics committee, the clinical physician, or the patient considers it necessary, the study may be immediately halted. 18
4.3.2 Known potential benefits 18

5.0 STUDY OBJECTIVES AND ENDPOINTS 19

5.1 Study Objectives 19
5.1.1 Primary objective 19
5.1.2 Secondary objectives 19
5.1.3 Exploratory objectives 19
5.2 Study Endpoints 19
5.2.1 Efficacy endpoints 19
5.2.2 Safety endpoints 19
5.2.3 Exploratory endpoints 19
5.3 Sample Size 19

WE ASSUMED THAT THE NULL HYPOTHESIS OF THE 12-MONTH PFS RATE WAS 40%, AND THE ALTERNATIVE HYPOTHESIS WAS 60%. WITH A SIGNIFICANCE LEVEL OF 0.1 (TWO-SIDED) AND A POWER OF 80%, 49 PATIENTS WERE REQUIRED. CONSIDERING A DROPOUT RATE OF 20%, THE STUDY NEEDEDS TO ENROLL 63 PATIENTS. 19

6.0 STUDY DESIGN AND DESCRIPTION 19

6.1 Study Design 19

6.2 Rationale for Study Design ... 20
6.3 Rationale for Dose Selection ... 20
6.4 Premature Termination or Suspension of the Study/Study Site ... 21
6.4.1 Criteria for premature termination or suspension of the study ... 21
6.4.2 Criteria for premature termination or suspension of study site ... 21
6.4.3 Procedure for premature termination or suspension of the study/study site ... 21
7.0 SCREENING OF SUBJECTS ... 21
7.1 Inclusion Criteria ... 21
7.2 Exclusion Criteria ... 21
7.3 Recruitment and Screening Number ... 23
7.4 Enrollment Status ... 24
7.5 Procedures for Managing Wrongly Enrolled Subjects ... 24
7.6 Limitations ... 24
7.7 Concomitant Medications and Treatments ... 24
7.7.1 Permitted concomitant treatments ... 24
7.7.2 Other anti-tumor/anti-cancer or investigational medicinal products ... 25
7.7.3 Prohibited medications ... 25
7.7.4 Glucocorticoids ... 25
7.7.5 Surgery (non-antineoplastic purpose) ... 25
7.8 Treatment Discontinuation and Withdrawal from Study ... 25
7.8.1 Treatment discontinuation ... 25
7.8.2 Withdrawal from study ... 26
8.0 MANAGEMENT OF INVESTIGATIONAL MEDICINAL PRODUCTS ... 26
8.1 Investigational Medicinal Products ... 26
8.1.1 Dosing regimen ... 26
8.1.2 Dosage form, manufacturing, packaging, and labeling ... 26
8.1.3 Storage ... 26
8.2 Dispensing Procedures of Investigational Medicinal Products ... 27
8.3 Medication Compliance ... 27
8.4 Management of Investigational Medicinal Products ... 27
9.0 STUDY PLAN AND PROCEDURES ... 27
9.1 Study Procedures ... 27
9.1.1 Screening period (D−28 to D0) ... 28
9.1.2 Treatment period ... 28
9.1.3 Follow-up period ... 29
9.2 Unscheduled Visits/Tests ... 30
10.0 STUDY EVALUATION ... 30
10.1 Study-Specific Procedures ... 30
10.1.1 Informed consent process ... 31
10.1.2 Screening number assignment ... 31
10.1.3 Screening and enrollment ... 31
10.1.4 Randomization number assignment ... 31
10.1.5 Data collection ... 31

10.1.6 Physical examination....31
10.1.7 ECOG PS score....31
10.1.8 Body weight and height measurement....31
10.1.9 Vital signs measurement....31
10.1.10 Laboratory tests....32
10.1.11 ECG....32
10.1.12 Echocardiography....32
10.1.13 Ophthalmological examination....32
10.2 Efficacy Evaluation....32
10.2.1 Response evaluation in solid tumors as per RECIST 1.1....32
10.2.2 EGFR mutation testing at screening....33
10.3 Safety Evaluation....33
10.4 Sampling and Testing Design....33
10.4.1 Exploratory sampling....33
10.4.3 Precautions for sampling....34
10.5 Management of Biological Samples....34
10.5.1 Sample collection volume....34
10.5.2 Destruction of biological samples....34
10.5.3 Labeling and supervision of biological samples....34
11.0 ADVERSE EVENTS AND MEDICAL MANAGEMENT....34
11.1 Adverse Events....34
11.1.1 Definition of adverse event....34
11.1.2 Definition of serious adverse event....35
11.1.3 Documentation of adverse events....35
11.1.4 Causal relationship determination....36
11.1.5 Adverse events based on symptoms and signs....36
11.1.6 Adverse events based on examinations and tests....36
11.1.7 Hy’s law....37
11.1.8 Progressive disease....37
11.1.9 New cancers....37
11.1.10 Handling of deaths....37
11.1.11 Reporting of adverse events....37
11.2 Overdose 37
11.3 Pregnancy Report....38
11.3.1 Pregnancy of female subjects....38
11.3.2 Pregnancy of partners of male subjects....38
11.4 Recommended Toxicity Management....38
11.4.1 Aumolertinib dose reduction....38
11.4.2 Dose modifications associated with adverse events....38
11.4.3 Skin reaction....40
11.4.4 Gastrointestinal toxicity....40
11.4.5 Interstitial lung disease....40
12.0 ENDPOINT EVALUATION AND STATISTICAL ANALYSIS....40
12.1 Anti-Tumor Activity Evaluation....40

12.1.1 Investigator assessment as per RECIST 1.1 40
12.1.2 Progression-free survival (PFS) 41
12.1.3 Objective response rate (ORR) 41
12.1.4 Duration of response (DoR) 41
12.1.5 Disease control rate (DCR) 41
12.1.6 Overall survival (OS) 41
12.2 Safety Evaluation 41
12.4 Statistical Analysis Methods 41
12.4.1 Definitions of statistical analysis sets 41
12.4.2 Efficacy analysis 42
12.4.3 Safety analysis 42
12.4.4 Pharmacokinetic analysis 43
13.0 DATA PROCESSING AND RETENTION 43
13.1 Electronic Case Report Form 43
13.2 Data Retention 43
REFER TO THE CLINICAL STUDY AGREEMENT FOR THE SPONSOR'S REQUIREMENTS FOR RECORD RETENTION. THE INVESTIGATORS SHOULD CONTACT AND RECEIVE WRITTEN APPROVAL FROM THE SPONSOR BEFORE DISPOSING OF ANY SUCH DOCUMENTS. 43
14.0 QUALITY CONTROL AND QUALITY ASSURANCE 43
14.1 Study Monitoring Visits 43
14.2 Protocol Deviations 43
14.3 Quality Assurance Audits and Official Inspections 43
15.0 ETHICS 44
15.1 IRB and/or IEC Approval 44
15.2 Subject Information, Informed Consent, and Subject Authorization 44
15.3 Privacy Protection 44
15.4 Publication, Disclosure, and Clinical Trial Registration 45
15.4.1 Publication and disclosure 45
15.4.2 Clinical study registration 45
15.4.3 Disclosure of clinical study results 45
15.5 Insurance and Compensation 45
16.0 MEDICAL EMERGENCIES AND CONTACT INFORMATION OF THE SPONSOR OR ITS DESIGNATED PERSONNEL 45
17.0 REFERENCES 46
APPENDIX A INVESTIGATORS' INFORMED CONSENT TO USE OF PERSONAL INFORMATION 49
APPENDIX B STUDY SCHEDULE 50
APPENDIX C ACTIONS REQUIRED FOR CONCOMITANT ELEVATIONS OF AMINOTRANSFERASES AND TOTAL BILIRUBIN - HY'S LAW 52
APPENDIX D EVALUATION OF OBJECTIVE TUMOR RESPONSE USING RESPONSE EVALUATION CRITERIA IN SOLID TUMORS GUIDELINES 1.1 (RECIST 1.1) 54
APPENDIX E. LIST OF MEDICATIONS TO BE AVOIDED (INCLUDING BUT NOT LIMITED TO THE FOLLOWING) 60
APPENDIX F. QTC FRIDERICIA'S FORMULA 62

APPENDIX G. CREATININE CLEARANCE FORMULA (COCKCROFT-GAULT FORMULA) 63

APPENDIX H. SURGICAL GRADING IN *ADMINISTRATIVE MEASURES FOR THE APPLICATION OF MEDICAL TECHNOLOGY* (2018 EDITION) 64

APPENDIX I DISTRIBUTION OF PROLIFERATING BONE MARROW IN ADULT CANCER PATIENTS 65

203 2.0 STUDY SYNOPSIS

<table>
<tr><td>Sponsor: Cancer Hospital of the University of Chinese Academy of Sciences (Zhejiang Cancer Hospital)</td><td>Investigational medicinal product: aumolertinib (HS-10296)</td></tr>
<tr><td colspan="2">Protocol Title: High-Dose Aumolertinib as First-Line Treatment in Patients with EGFR-Sensitive Mutations in Advanced NSCLC with Brain Metastases: Prospective, Open-Label, Multi-center, Single-Arm Clinical Trial</td></tr>
<tr><td colspan="2">Study No.: YX-L-202107</td></tr>
<tr><td colspan="2">Study design:
This prospective, open-label, multi-center, single-arm clinical trial is intended to evaluate the efficacy and safety of high-dose aumolertinib mesylate (hereinafter referred to as "aumolertinib") in patients with epidermal growth factor receptor-sensitive mutant (EGFRm^{+}) advanced non-small cell lung cancer (NSCLC) with brain metastases who have not received any systemic treatment. Eligible patients will be enrolled in the high-dose aumolertinib group and receive aumolertinib 165 mg p.o., QD.
Study process:
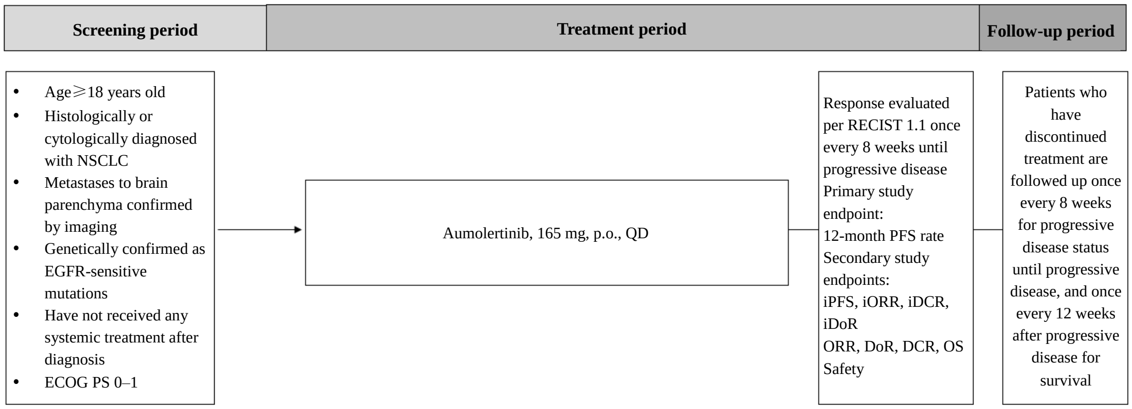
</td></tr>
<tr><td>Duration of treatment:
One cycle of study treatment is defined as 4 consecutive weeks of administration. The treatment will continue until progressive disease (if the investigator believes that the patient can continue to benefit clinically from the treatment, the patient can continue to receive the drug even if the progressive disease is present as per RECIST 1.1) or meeting the termination criteria.</td><td>Study duration:
According to the study design, enrollment is planned to last for 6 months and follow-up 20 months</td></tr>
<tr><td>Number of subjects:
Estimated total: about 63</td><td>Number of sites:
Estimated total: 20</td></tr>
<tr><td colspan="2">Dosage and administration:
Aumolertinib tablets: strength: 55 mg/tablet; dose: 165 mg/day (3 tablets/day), p.o., QD.
Note: It should be taken under fasting or fed condition, and swallowed as a whole with an appropriate amount of warm water without chewing or crushing.</td></tr>
<tr><td colspan="2">Subject population:
Patients age≥18 years old, with histologically or cytologically confirmed NSCLC, which is confirmed by imaging as advanced NSCLC with metastases to brain parenchyma. The patients should not receive any systemic treatment in the advanced stage, and are confirmed with EGFR-sensitive mutations before enrollment (exon 19 deletion or L858R, either existing alone or co-existing with other EGFR site mutations)</td></tr>
<tr><td colspan="2">Primary study objective:
To evaluate the 12-month progression-free survival (PFS) rate of high-dose aumolertinib as first-line treatment of EGFR-sensitive mutant advanced NSCLC with brain metastases</td></tr>
<tr><td colspan="2">Secondary study objectives:
1. To evaluate other anti-tumor efficacy endpoints of high-dose aumolertinib:
Intracranial: intracranial progression-free survival (iPFS), intracranial objective response rate (iORR), duration of intracranial response (iDoR), and intracranial disease control rate (iDCR);
Systemic: objective response rate (ORR), overall survival (OS), duration of response (DoR), and disease control rate (DCR).
2. To evaluate the safety of high-dose aumolertinib.</td></tr>
</table>

Exploratory study objectives:
To explore the dynamic change of blood gene mapping before and after treatment and its correlation with efficacy.

Inclusion criteria:
A subject must meet all the following inclusion criteria in order to be enrolled in this study:

1. Age≥18 years old.
2. Histologically or cytologically confirmed NSCLC, which is confirmed by imaging as advanced NSCLC with metastases to brain parenchyma (including stage IV patients who are newly diagnosed or have relapsed after previous surgical treatment; according to the AJCC 8th edition lung cancer staging criteria).
3. Confirmed as EGFR-sensitive mutations in tumor tissue samples or blood samples by local laboratory (including exon 19 deletion or L858R, either existing alone or co-existing with other EGFR site mutations). If the tumor tissue is not available or the patient does not accept tissue biopsy, a blood sample is acceptable.
4. Have not received any systemic anti-tumor treatment after the diagnosis of advanced NSCLC with metastases to brain parenchyma. For patients who have received local treatment, the lesion within the scope of the local treatment cannot be used as the target lesion unless the lesion has progressed.
5. Patients with at least 1 extracranial target lesion (measurable lesions that have not undergone local treatment such as irradiation or have shown definitive after local treatment, with a baseline longest diameter ≥ 10 mm (or ≥ 15 mm for lymph nodes)) and at least 1 intracranial lesion (with a baseline longest diameter ≥ 5 mm).
6. The brain condition is stable or that were controlled with anticonvulsants or dexamethasone at a maximum dose of 5 mg daily before the study treatment.
7. The Eastern Cooperative Oncology Group (ECOG) performance status score is 0 or 1, and has not deteriorated at least 2 weeks before the study treatment, and the expected survival period is not less than 3 months.
8. Female patients of childbearing potential are willing to take appropriate contraceptive measures and should not breastfeed from signing the informed consent to 6 months after the last dose of study treatment; male patients are willing to take barrier contraception (i.e., condoms) from signing the informed consent to 6 months after the last dose study treatment.
9. Female patients of childbearing potential must have a negative serum or urine HCG test within 7 days before enrollment in the study, and they must be non-lactating.
10. The subject voluntarily participates and signs the informed consent form in writing.

Exclusion criteria:
A subject will not be eligible for inclusion in this study if any of the following criteria apply:

1. Treatment with any of the following:
   a. Previously received EGFR tyrosine kinase inhibitor (EGFR-TKI) treatment (such as erlotinib, gefitinib, icotinib, afatinib, osimertinib, and aumolertinib);
   b. The patient has undergone major surgery (such as craniotomy, thoracotomy, or laparotomy) within 4 weeks before the first dose of the investigational medicinal product, or undergone minor traumatic surgery (biopsy, bronchoscopy, and thoracic drainage) within 7 days before the first dose of the investigational medicinal product. Major surgeries are defined as Grade 3 and Grade 4 surgeries specified in the "Administrative Measures for Clinical Application of Medical Technology" implemented from Nov. 1, 2018 in Appendix H;
   c. Patients who have received local radiotherapy (palliative bone radiotherapy for non-target lesions) within 2 weeks before the first dose of the investigational medicinal product; patients who have received more than 30% of bone marrow irradiation (see Appendix I for the calculated area of bone marrow) or extensive radiotherapy within 4 weeks before the first dose of the investigational medicinal product; patients who have received whole brain radiotherapy for this disease before enrollment;
   d. Recurrence within 6 months after adjuvant or neoadjuvant treatment for early lung cancer; if there is both neoadjuvant therapy and adjuvant therapy, the adjuvant treatment time will be used for calculation;
   e. Within 14 days before the first dose of the investigational medicinal product, traditional Chinese medicines and their preparations with anti-tumor effect or adjuvant anti-tumor effects have been used (see Appendix E for the list of drugs);
   f. Presence of pleural/abdominal effusion requiring clinical intervention (patients who do not require drainage of the effusion or are stable for more than 2 weeks after drainage of the effusion are eligible); presence of pericardial effusion (patients with small amount of pericardial effusion who are stable for 2 weeks or more are eligible). If anti-tumor drugs are used locally (e.g., via intrapleural infusion) during drainage, patients are only eligible with a washout of at least 5 half-lives or 21 days (whichever is shorter) prior to the first dose of study treatment;
   g. Previously treated with drugs that are strong inhibitors or strong inducers of CYP3A4, or treated with

CYP3A4 sensitive substrate drugs with a narrow therapeutic window, within 7 days prior to the first dose of investigational medicinal product; or requiring to continue the treatment with these drugs during the study (see Appendix E for the list of these drugs);

h. Receiving drugs known to prolong the QT interval or that may cause torsade de pointes, or requiring to continue the treatment with these drugs during the study (see Appendix E for the list of drugs and washout times);
i. Having participated in another clinical trial within 4 weeks or been still within 5 half-lives of the investigational medicinal product in another clinical trial before the first dose of this investigational medicinal product, whichever is longer (except for screening failure).

2. Histologically or cytologically confirmed mixed SCLC and mixed NSCLC, large cell neuroendocrine carcinoma and sarcomatoid carcinoma.
3. At the beginning of study treatment, those with residual toxicity from prior anti-tumor treatment greater than CTCAE Grade 1 that cannot be alleviated, with the exception of hair loss and Grade 2 neurotoxicity from prior anti-tumor treatment. History of intracranial hemorrhage not related to tumor.
4. History of other primary malignant tumors, except for the following:
   a. Radically treated malignancies that have been inactive for ≥ 5 years prior to study enrollment and have a very low risk of recurrence;
   b. Adequately treated non-melanoma skin cancer or lentigo maligna without evidence of disease recurrence;
   c. Adequately treated carcinoma *in situ* without evidence of disease recurrence.
5. Diagnosis of meningeal metastasis through clinical symptoms or imaging or cerebrospinal fluid, or brain parenchymal metastasis combined with meningeal metastasis.
6. Patients who are allergic to MRI contrast agent gadolinium or who cannot tolerate MRI examinations (such as patients with pacemakers or metals in the body).
7. Any serious or poorly controlled systemic disease, such as poorly controlled hypertension, active bleeding diathesis, or active infection, as judged by the investigator. There is no need to check for chronic diseases.
8. Clinically serious abnormal gastrointestinal function, which may affect the intake, transport or absorption of drugs, such as inability to take drugs orally, uncontrollable nausea or vomiting, history of extensive gastrointestinal resection, uncured recurrent diarrhea, atrophic gastritis, uncured gastric diseases that require proton pump inhibitors for a long time, Crohn's disease, and ulcerative colitis.
9. Hepatic encephalopathy, hepatorenal syndrome, or cirrhosis.
10. Any of the following cardiac findings:
    a. Mean QT interval corrected by Fridericia (QTcF) > 470 msec from 3 repeated measurements of electrocardiogram (ECG) at rest;
    b. Any clinically significant abnormalities in rhythm, conduction, or ECG morphology determined by the investigator (e.g., complete left bundle branch block, third-degree atrioventricular block, second-degree atrioventricular block, and PR interval > 250 msec), as indicated by ECG at rest;
    c. Presence of any factor that increases the risk of QTc prolongation or arrhythmic events, such as cardiac failure, hypokalemia, congenital long QT syndrome, family history of long QT syndrome, or unexplained sudden death of immediate family members under 40 years of age, or any concomitant medications known to prolong QT interval;
    d. Left ventricular ejection fraction (LVEF) < 50%.
11. Insufficient bone marrow reserve or organ function, meeting any of the following laboratory test limits (no corrective treatment within 1 week before blood collection for laboratory tests):
    a. Absolute neutrophil count < 1.5×10^9/L;
    b. Platelet count < 100×10^9/L;
    c. Hemoglobin < 90 g/L (< 9 g/dL);
    d. Alanine aminotransferase > 2.5 × upper limit of normal (ULN) in the absence of demonstrable liver metastases; alanine aminotransferase > 5 × ULN in the presence of liver metastases;
    e. Aspartate aminotransferase > 2.5 × ULN in the absence of demonstrable liver metastases; aspartate aminotransferase > 5 × ULN in the presence of liver metastases;
    f. Total bilirubin > 1.5 × ULN in the absence of demonstrable liver metastases, or total bilirubin > 3 × ULN in the presence of Gilbert's syndrome (unconjugated hyperbilirubinemia) or liver metastases;
    g. Creatinine > 1.5 × ULN and creatinine clearance < 50 mL/min (calculated by Cockcroft-Gault formula); creatinine clearance should be determined only when creatinine is > 1.5 × ULN;
    h. Serum albumin (ALB) < 28 g/L;
12. Female subjects who are pregnant and lactating or plan to become pregnant during the study period.

13. History of interstitial lung disease, drug-induced interstitial lung disease, radiation pneumonitis requiring steroid therapy, or any evidence of clinically active interstitial lung disease.
14. History of hypersensitivity to any active or inactive ingredients of aumolertinib or drugs of similar chemical structure or the same class as aumolertinib.
15. Patients with eye disorders that may increase the safety risk of patients as judged by the investigator (especially severe dry eye syndrome, dry keratoconjunctivitis, severe exposure keratitis, or other diseases that may increase epithelial damage) and require surgery or eye abnormalities that are expected to require surgery during the study.
16. Patients who may have poor compliance with the procedures and requirements of the study as judged by the investigator, such as patients who have a clear history of neurological or mental disorders (including epilepsy or dementia), or patients who are currently suffering from mental disorders.
17. Patients with any conditions that may jeopardize patient safety or interfere with study assessments as judged by the investigator.

Efficacy evaluation:
To evaluate the efficacy of high-dose aumolertinib as first-line treatment in patients with EGFR-sensitive mutant advanced NSCLC with brain metastases as per Response Evaluation Criteria in Solid Tumors 1.1 (RECIST 1.1).
Efficacy endpoints:
1. Primary endpoint:

12-month PFS rate

2. Secondary endpoints:

Intracranial: iPFS, iORR, iDoR, iDCR
Systemic: ORR, DoR, DCR, OS

Safety evaluation:
To observe and record any adverse events (AEs) occurring in all subjects during the clinical trial, including abnormal clinical symptoms and vital signs and laboratory abnormalities; to record the severity, time of onset, duration, treatment and prognosis, and determine the causality between AEs and the investigational medicinal product.
Safety endpoints: occurrence of AEs; occurrence of SAEs; proportion of patients withdrawn due to AEs; changes in laboratory tests (blood biochemistry, hematology, and urinalysis); changes in vital signs, physical examination, body weight, ECG, LVEF, ECOG PS score, and ophthalmological examination.

Exploratory evaluation:
Venous blood: To explore the dynamic change of blood gene mapping before and after treatment and its correlation with efficacy.

Sample size calculation:
This is an open-label, multi-center, prospective, single-arm clinical study. The primary objective is to evaluate the efficacy and safety of high-dose aumolertinib as first-line treatment in patients with EGFR-sensitive mutant advanced NSCLC with brain metastases. We assumed that the null hypothesis of 12-month PFS rate was 40%, and the alternative hypothesis was 60%. With a significance level of 0.1 (two-sided) and a power of 80%, 49 patients were required. Considering a dropout rate of 20%, the study needed to enroll 63 patients.

204

205 3.0 LIST OF ABBREVIATIONS

AE	Adverse event
ALP	Alkaline phosphatase
ALT	Alanine aminotransferase
ANC	Absolute neutrophil count
AR	Acquired resistance
AST	Aspartate aminotransferase
AUC_{ss}	Area under the plasma concentration-time curve during any dosing interval at steady state [amount·time/volume]
CFDA	China Food and Drug Administration
C1D1	Cycle 1 Day 1
CI	Confidence interval
CLint	Clearance of liver microsomes
C_{max}	Maximum plasma concentration
CR	Complete response
CT	Computed tomography
CTCAE	Common Terminology Criteria for Adverse Events
CYP	Cytochrome P450
DCR	Disease control rate
DLT	Dose-limiting toxicity
DNA	Deoxyribonucleic acid
DoR	Duration of response
ECG	Electrocardiogram
eCRF	Electronic case report form
ECOG	Eastern Cooperative Oncology Group
EGFR	Epidermal growth factor receptor
$EGFRm^{+}$	Epidermal growth factor receptor sensitive mutation positive
FAS	Full analysis set
FDA	Food and Drug Administration
FSH	Follicle-stimulating hormone
GCP	Good Clinical Practice
HAS-719	A major circulating metabolite of aumolertinib
HER	Human epidermal growth factor receptor
hERG	Human ether-à-go-go-related gene
HIV	Human immunodeficiency virus
HL	Hy's law
HNSTD	Highest non-severely toxic dose
HR	Hazard ratio
HRCT	High-resolution computed tomography
iPFS	Intracranial progression-free survival
iDCR	Intracranial disease control rate
iDoR	Intracranial duration of response
iORR	Intracranial objective response rate
IC_{50}	Half-maximal inhibitory concentration
ICH	International Conference on Harmonization of Technical Requirements for Registration of Pharmaceuticals for Human Use
IDMC	Independent Data Monitoring Committee

IEC	Independent ethics committee
INR	International normalized ratio
IRB	Institutional review board
LH	Luteinizing hormone
LPLV	Last patient last visit
IWRS	Interactive web response system
LVEF	Left ventricular ejection fraction
MedDRA	Medical Dictionary for Regulatory Activities
mPFS	Median progression-free survival
MRI	Magnetic resonance imaging
MTD	Maximal tolerated dose
MUGA	Multigated acquisition
NE	Inevaluable
NOAEL	No observed adverse effect level
NSCLC	Non-small cell lung cancer
NTL	Non-target lesion
ORR	Objective response rate
OR	Odds ratio
OS	Overall survival
PD	Disease progression
PFS	Progression-free survival
PFS2	Progression-free survival 2
PHL	Potential Hy's Law
PK	Pharmacokinetics
PPS	Per-protocol set
PR	Partial response
PS	Performance status
PT	Preferred term
QD	Quaque die
QRS	Combination of three connected waves seen on a typical ECG
QT	ECG interval measured from the beginning of the QRS complex to the end of the T wave
QTc	Corrected QT interval
QTcF	Fridericia-corrected QT interval
RECIST 1.1	Response Evaluation Criteria in Solid Tumors 1.1
SAE	Serious adverse event
SS	Safety set
SD	Stable disease
STD_{10}	Severely toxic dose in 10% of animals
SOC	System organ class
T790M	Threonine-to-methionine substitution at amino acid position 790 of EGFR
$T790M^{+}$	T790M mutation-positive
TBiL	Total bilirubin
TL	Target lesion
TKI	Tyrosine kinase inhibitor
ULN	Upper limit of normal
WT	Wild type

206

4.0 STUDY BACKGROUND AND SCIENTIFIC RATIONALE

4.1 Study Background

Lung cancer is one type of malignant tumor with the highest morbidity and mortality in the world. The cancer data published in China in 2018 revealed that lung cancers have the highest morbidity and mortality in China, accounting for more than 20% of all cancers. Non-small cell lung cancer (NSCLC) accounts for 80%–85% of all lung cancers. Approximately 70% of lung cancer cases have reached the advanced stages of locally advanced status or distant metastasis at the time of diagnosis and cannot be treated by surgical resection. In addition, most patients who have undergone surgical resection at early stages have distant metastases at the time of recurrence, which often leads to death[1]. Usually, the median survival of patients with advanced NSCLC who are unable to undergo surgical resection is less than one year[2].

In recent years, targeted drugs based on driver genes have continuously emerged. Epidermal growth factor receptor (EGFR) sensitive mutation (EGFRm^{+}) is one of the main driver genes in NSCLC, accounting for 10%–17% in Western populations and 30%–50% in Asian populations[3]. As first-generation EGFR tyrosine kinase inhibitors (TKIs), gefitinib and erlotinib showed good initial responses in NSCLC patients with EGFR-sensitive mutations. However, most patients develop acquired resistance (AR) after 9–13 months of treatment, leading to disease exacerbation[4]. In addition, these first-generation TKIs often cause skin and gastrointestinal adverse reactions such as rash and diarrhea due to the inhibition of wild-type (WT) EGFR[5].

Several mechanisms of AR have been reported, such as EGFR second-site mutation, alternative activation such as HER2 and MET, or phenotypic transformation. Among them, the most common resistance mechanism is the gatekeeper mutation of threonine to methionine at the amino acid 790 of EGFR (T790M), which has been detected in more than 50% of patients who have progressed after gefitinib or erlotinib treatment[6]. In addition, some patients harbor primary T790M mutations prior to EGFR TKI therapy. Using sensitive detection techniques (time-of-flight mass spectrometry and next-generation sequencing), these mutations can be identified in approximately 30% of patients with EGFR-sensitive mutations, which is higher than the previously reported rate of 2%–3%[7]. Regardless of whether the drug resistance mutation is acquired or primary, the development of new treatment regimens and the early use of third-generation EGFR TKIs that have inhibitory effects on T790M drug resistance mutations and EGFR-sensitive mutations delay the onset of drug resistance mutations, and better clinical benefits are expected for advanced NSCLC.

Third-generation EGFR TKIs can selectively target the EGFR T790M mutation and reduce the toxicities caused by EGFR wild-type inhibition. Osimertinib (AZD9291), the first third-generation EGFR TKI, was approved for marketing by the FDA in Nov. 2015 and in China in Mar. 2017. The current indications include the second-line treatment of NSCLC after failure of first-generation TKIs and the first-line treatment of advanced NSCLC with EGFR-sensitive mutations.

The incidence of brain metastases in patients with EGFR-sensitive mutations is also increasing year by year[8]. Patients with EGFR-sensitive mutations are more likely to develop brain metastases compared with patients with EGFR wild-type NSCLC[9]. The EGFR mutation rate is higher in NSCLC patients with CNS metastases, up to 63% in Asian population[10]. This trend may be caused by the following: 1) This may be related to improvements in imaging techniques and routine screening that enable early detection and diagnosis of CNS metastases[11]; 2) Existing EGFR TKIs effectively control non-CNS lesions and prolong the overall survival of patients with EGFR mutation-positive NSCLC correspondingly[12–15]; 3) The efficacy of existing EGFR TKIs on CNS metastases is limited. The blood-brain barrier (BBB) blocks most drugs from entering the intracranial circulation. Studies have shown that the current approved first- and second-generation EGFR TKIs have limited ability to pass through the BBB, so the therapeutic or preventive effects on CNS-metastatic tumors are minimal[16]. In a study in China, lung cancer patients with brain metastases received gefitinib at a dose of 250 mg per day, and its permeability rate in cerebrospinal fluid was only about 2%[17].

The ability of third-generation EGFR TKIs to pass through the BBB was significantly improved. A retrospective analysis showed that in EGFR-sensitive mutant NSCLC patients with brain metastases, the mOS was 16.9 months and the mPFS was 7.6 months with the third-generation EGFR TKI osimertinib at a high dose of 160 mg as second-line treatment[18, 19]. Similarly, the results of the BLOOM study suggested that osimertinib, a third-generation EGFR TKI, showed meaningful efficacy in the first-line treatment of EGFR-sensitive mutant NSCLC with CNS metastases, but there is still room for optimization.

4.2 Scientific Rationale

Aumolertinib mesilate (HS-10296) is a class 1 innovative drug independently developed by Jiangsu Hansoh Pharmaceutical Group Co., Ltd. It is a third-generation small molecule EGFR TKI, which can irreversibly and selectively inhibit EGFR-sensitive mutations (e.g., exon 19 deletion and L858R mutation) and T790M resistance mutations, with very low activity against wild-type (WT) EGFR. In Mar. 2020, aumolertinib was approved by the

CFDA (now NMPA) for the second-line treatment of advanced NSCLC with EGFR-sensitive mutations.

4.2.1 Mechanism of action and pharmacology

In *in vitro* enzyme and cell proliferation studies, aumolertinib exhibited potent inhibitory effect on EGFR T790M resistance mutations and weaker inhibitory effect on WT EGFR[20]. HAS-719 is a major metabolite of aumolertinib, with similar enzymatic activity profile to that of its parent drug, aumolertinib[20]. Aumolertinib has little-to-no inhibitory effect on 38 closely related kinases, suggesting that aumolertinib is a selective EGFR mutant kinase inhibitor with no potential off-target effect[21].

In a nude mouse tumor model, aumolertinib potently inhibited tumor growth of NSCLC H1975 and LU1868 cell lines harboring EGFR T790M mutations, and also inhibited tumor growth of NSCLC HCC827 cell lines harboring EGFR-sensitive mutations[22]. In the 20 mg/kg high-dose group, aumolertinib led to almost complete regression of tumors harboring EGFR mutations. Aumolertinib had weak inhibitory effect on WT EGFR A431 cell lines, suggesting a better safety profile compared to other EGFR inhibitors. Aumolertinib could potently and specifically inhibit EGFR T790 resistant mutations and had weak inhibitory effects on WT EGFR, fully reflecting the characteristics of the new generation of EGFR inhibitors.

In an *in vitro* study of the potassium channel encoded by hERG (human *ether-à-go-go* gene), the half maximal inhibitory concentration (IC_{50}) of aumolertinib was 2.958 μM, indicating that aumolertinib has a slight inhibitory effect on hERG current[23]. However, in the NCI-H1975 xenograft tumor model, the maximum plasma concentration (C_{max}) of aumolertinib at the effective dose of 5 mg/kg was 190 ng/mL, which was much lower than hERG IC_{50}, and the difference was up to 12.2-fold[22]. In addition, the plasma protein binding of aumolertinib was greater than 99.5% in rats, dogs, and humans, resulting in very low concentrations of unbound aumolertinib[24]. No adverse effects were observed in the central nervous system (rats), cardiovascular system, and respiratory system (dogs) in animal studies[25, 26]. All these results suggest a low likelihood of cardiotoxicity caused by aumolertinib.

Aumolertinib was administered after the intracranial xenograft models in rats were established using EGFR T790M/L858R double-mutant NSCLC NCI-H1975 cells and NSCLC PC-9 cells harboring EGFR Del19, and then the intracranial fluorescence intensity and survival status of mice were measured. The results showed that aumolertinib at 20 mg/kg and 40 mg/kg (equivalent to clinical doses of 110 mg and 220 mg) had significant efficacy on NCI-H1975 and PC-9 intracranial *in situ* xenograft models, with a good dose-response relationship, and the survival of animals was significantly longer than that in the control group. The above results suggest that a high dose of 165 mg or 220 mg aumolertinib has good clinical exploratory value.

4.2.2 Nonclinical pharmacokinetics (PK) and drug metabolism

In the pharmacokinetic studies in rats and dogs, aumolertinib was rapidly absorbed after oral administration[27, 28], and its major metabolite, HAS-719, was produced by N-demethylation. The area under the plasma concentration-time curve (AUC) of aumolertinib and HAS-719 increased in a dose-proportional manner, showing a significant linear relationship. No significant accumulation was observed after repeated doses of aumolertinib.

The plasma protein binding of aumolertinib and HAS-719 was ≥ 99.5% in rats, dogs, and humans[24]. The peak concentration of aumolertinib was reached within 2 h following oral gavage to rats[29]. Aumolertinib and its metabolite HAS-719 were widely distributed in tissues, and the concentration of aumolertinib in most tissues was higher than that in plasma. Aumolertinib was mainly distributed in lung, adrenal gland, spleen, bone marrow, and liver. Aumolertinib could readily penetrate the BBB with a brain-to-plasma exposure ratio > 7. A total of 36 metabolites could be detected in rat plasma following oral gavage of aumolertinib. Cumulative urinary excretion of aumolertinib and HAS-719 was minimal, less than 0.1% of the administered dose[30], while the excretion was 3.4% and 0.2% in feces and bile, respectively, within 0 to 96 h following the oral gavage of aumolertinib to rats.

Based on the clearance of liver microsomes (CL_{int}) in different species, aumolertinib is speculated to be a drug with moderate clearance in humans and dogs, and with high clearance in mice, rats, and monkeys[31]. In a study of inter-species differences in hepatocyte metabolism in mice, rats, dogs, monkeys, and humans, aumolertinib was adequately metabolized, and no specific metabolites were found in humans[32, 33]. *In vitro* studies have shown that aumolertinib had no inhibitory effect on the major human metabolic enzyme, cytochrome P450 (CYP450), and it had no inductive effect on CYP1A2, CYP2B6 and CYP3A4[34]. Therefore, its potential of causing drug-drug interactions via inhibition and induction of these CYP450s is very low[35]. The results of the permeability study in the Caco-2 cell model showed that aumolertinib had low permeability and that efflux transporters were involved in the transport of aumolertinib on Caco-2 cells[36].

4.2.3 Nonclinical toxicology

In an acute toxicity study, no death was found at the highest dose of 900 mg/kg in rats and 200 mg/kg in dogs[37, 38]. Clinical observations revealed fluffy hair, piloerection, prone position, humped back, decreased activity, loose/soft stools, and perianal feces in rats of the high-dose group. Vomiting, loose stool, or bloody feces were observed in dogs.

In a 13-week repeat-dose long-term toxicity study, death was observed in female rats at 120 mg/kg and dogs at 25 mg/kg[39-43]. The toxicities of aumolertinib observed in rats and dogs were mainly manifested in the skin, gastrointestinal tract, and eyes, and the analysis showed that toxicities might be mainly secondary to the potent inhibition of EGFR by the drug or related to significant reductions in body weight and/or food intake. These toxicities were reversible after a 4-week recovery period.

Aumolertinib-related toxicities in rats included soft stools, salivary secretion, skin damage, and eye abnormalities. Histopathological findings were mainly observed in the mammary gland and vagina of rats, as well as the tongue, skin, oral cavity, and thymus of dogs. The skin damages observed in rats and dogs were associated with dermal folliculitis, which is consistent with the literature report that EGFR inhibitors could cause skin damage in rodents and humans[44-46]. The ulcer and inflammatory reactions observed in the tongues of dogs were similar to the adverse reactions caused by tyrosine kinase inhibitors in humans[44, 47]. In addition, changes in the oral mucosa have also been reported in the study with anti-EGFR antibody therapy[48].

Evident ocular toxicities in rats and dogs, such as conjunctival hyperemia, corneal changes, and other eye abnormalities, have been reported in patients treated with anti-EGFR therapy[49, 50]. Mastatrophy and mucification of the vaginal epithelium were considered reactions secondary to treatment-related changes in food intake and body weight[51, 52]. Thymic involution/atrophy was considered to be caused by stress response[53]. The severely toxic dose in 10% of animals (STD_{10}) was 60 mg/kg/day in female rats and 120 mg/kg/day in male rats, and the highest non-severely toxic dose (HNSTD) in dogs was 10 mg/kg/day.

Both *in vitro* and *in vivo* genotoxicity studies showed negative results, suggesting that aumolertinib did not induce mutations or cause chromosome breakage at relevant concentrations[54-56].

The no-observed-adverse-effect level (NOAEL) of aumolertinib on reproduction and early embryonic development was 100 mg/kg/day for male rats and 30 mg/kg/day for female rats[57]. Statistically significant decreases in mean gravid uterus weight, implantation sites, and number of live fetuses were noted in female rats at 100 mg/kg/day. However, no drug-related changes were observed in sperm count, motility, and sperm morphology test in male rats at 100 mg/kg/day. Aumolertinib has no teratogenic effect on rat embryo-fetal development, with a NOAEL of 100 mg/kg/day[58].

4.2.4 Clinical studies

1) Clinical study of aumolertinib in NSCLC

The IND application for aumolertinib has been approved by the US FDA, TFDA in Taiwan, China, and NMPA in mainland China, respectively. The international multicenter phase I/II clinical studies of second-line treatment in advanced NSCLC patients with EGFR T790M mutations began in 2017, and the multicenter phase III clinical studies of first-line treatment in advanced NSCLC patients with EGFR-sensitive mutations began in 2018. In Mar. 2020, aumolertinib was approved by the CFDA (now NMPA) for the second-line treatment of advanced NSCLC with EGFR-sensitive mutations.

A total of 26 subjects were included in the phase I escalation study of aumolertinib. No dose-limiting toxicity (DLT) was observed in the low-dose group (55/110 mg), and only 1 DLT (Grade 3 anemia) was observed in 6 subjects of the 220 mg dose group, and only 1 DLT (Grade 3 blood creatine phosphokinase increased) was observed in 6 subjects of the 260 mg dose group. No maximal tolerated dose (MTD) was reached in the dose-escalation study.

The results of PK study showed that, in patients with advanced NSCLC who had undergone disease progression after previous EGFR TKI therapy, a single oral dose of aumolertinib administered under fasted conditions was rapidly absorbed, the peak plasma concentration was reached at 4.0 h, and the mean elimination half-life of the drug ranged from 31.29 to 35.19 h. The PK parameters of C_{max} and AUC_{0-t} of aumolertinib were generally linearly related to dose within the dose range of 55 to 260 mg. The exposure of the active metabolite HAS-719 was about 1/3 of that of the parent drug, and the PK parameters also showed a linear relationship. The time to reach peak concentration of HAS-719 was about 6 h later than that of the parent drug, and the mean elimination half-life ranged from 49.75 to 63.75 h. The data of multi-dose PK studies showed that following aumolertinib was administered once daily (QD) for 7 consecutive days, its plasma concentrations reached steady state, with little accumulation at steady state compared with single-dose administration. Within the dose range from 55 mg to 260 mg, when the steady state was reached following continuous dosing, the exposure of aumolertinib showed a basically linear relationship with the plasma concentration at steady state and AUC.

In terms of treatment response, the overall objective response rate (ORR) was 42.3% and the disease control rate (DCR) was 80.8% in 26 subjects. For subjects with partial response, there were 3 (3/6, 50.0%) and 4 (4/6, 66.7%) in the 55 mg and 110 mg groups, respectively, with a disease control rate of 100%; the ORR was 37.5% and 16.7%, and the DCR was 75.0% and 50.0% in the 220 mg and 260 mg groups, respectively.

The enrollment of T790M mutation-positive population for the phase I extension study of aumolertinib has been completed, with a total of 94 subjects enrolled in 55 mg (30), 110 mg (33), and 220 mg (31) dose groups. In terms of

safety, common AEs included creatine phosphokinase increased (24.47%), rash (18.09%), cough (18.09%), aspartate aminotransferase increased (13.83%), alanine aminotransferase increased (11.7%), white blood cell count decreased (11.7%), pruritus (11.7%), pain in extremity (14.89%), upper respiratory tract infection (11.7%), urinary tract infection (11.7%), diarrhea (15.96%), back pain (10.64%), and insomnia (10.64%). The overall incidence of AEs was higher in the 220 mg group than that in the 55 mg and 110 mg groups. In terms of treatment response, the overall ORR was 56.4% and the DCR was 91.5% in 94 subjects. Of these, the ORR was 60.0%, 54.5%, and 54.8%, and the DCR was 83.3%, 97.0%, and 93.5%, respectively, in the 55 mg, 110 mg, and 220 mg groups.

In addition, the study showed that food had no significant effect on the PK parameters of aumolertinib and its metabolite HAS-719. For aumolertinib, the time to peak concentration and peak concentration after high-fat meals were not significantly different from those after fasting, but the AUC increased by about 20%; for metabolite HAS-719, the time to peak concentration was prolonged, C_{max} decreased by about 18%, and the AUC remained the same. Based on the above study results, dosing under fasting conditions or after a meal is recommended in clinical studies.

Based on the safety and efficacy data from the phase I dose escalation and dose expansion studies, in combination with the PK data, the dosing regimen of 110 mg once daily in the phase II dose extension study was determined. A total of 244 subjects were included in the phase II dose extension study of aumolertinib. In terms of efficacy, the ORR was 68.9% (95% CI: 62.6, 74.6), the DCR was 93.4% (95% CI: 89.6, 96.2), and the mPFS was 12.3 months (95% CI: 9.6, 13.8) in 244 subjects. In terms of safety, the 110 mg dose showed good safety and tolerability, and 93.9% of subjects experienced at least one AE. Common AEs (incidence ≥ 5%) in the phase II dose extension included blood creatine phosphokinase increased (48, 19.7%), rash (31, 12.7%), aspartate aminotransferase increased (30, 12.3%), alanine aminotransferase increased (28, 11.4%), white blood cell count decreased (27, 11.1%), pruritus (26, 10.7%), anemia (22, 9.0%), diarrhea (18, 7.4%), leukopenia (17, 7%), proteinuria (17, 7%), platelet count decreased (16, 6.5%), and neutrophil count decreased (15, 6.1%). A total of 49 (20.1%) subjects experienced SAEs, of which 13 (5.3%) were related to the investigational medicinal product.

Aumolertinib has a good control effect on brain metastases. The central nervous system (CNS) response assessed by IRC using RECIST v1.1 was summarized as follows: the CNS ORR was 60.9% (95% CI: 38.5%, 80.3%), with one (4.3%) patient in complete response (CR), the CNS DCR was 91.3% (95% CI: 72.0%, 98.9%), the CNS mPFS was 10.8 months, and the CNS mDoR was 11.3 months.

At present, the clinical trials of the third-generation EGFR TKI of the same kind in NSCLC patients with brain metastases are also ongoing. In studies related to osimertinib, the AURA series of clinical trials suggested that, as a second-line treatment, osimertinib had a median PFS of about 7–9 months in subjects with CNS metastases, while the overall PFS was relatively long, about 10–12 months (AURA extension, AURA2, and AURA3). Compared to the traditional first- and second-generation EGFR TKIs, which have a PFS of 9.6 months in the population with CNS metastases, the osimertinib group demonstrated a significant advantage in first-line treatment of brain metastases, with a PFS of 15.2 months (FLAURA).

Based on the above preclinical and clinical data, this study aims to evaluate the efficacy and safety of high-dose aumolertinib as first-line treatment in subjects with EGFR-sensitive mutant advanced NSCLC with brain metastases who have not received any systemic treatment.

4.3 Potential Risks and Benefits

4.3.1 Potential risks and control plan

Previous clinical studies of aumolertinib suggested that the changes in gastrointestinal tract and skin reactions as well as laboratory test results should be closely monitored. The following table shows the common and possible adverse reactions in previous clinical trials of aumolertinib.

Table 1. Common and possible adverse reactions of aumolertinib

Most common adverse reactions	• Rash: including rash, rash pruritic, macule, rash maculo-papular, papule, rash papular, folliculitis, erythema, erythema nodosum, dermatitis, dermatitis acneiform, blister, urticaria, drug eruption, palmar-plantar erythrodysaesthesia syndrome, and photosensitivity reaction<br>• Investigation abnormal: blood creatine phosphokinase increased, aspartate aminotransferase increased, and alanine aminotransferase increased<br>• Pruritus: including pruritis and pruritus generalised

Serious adverse reactions	• Possible interstitial lung disease, often manifested as pulmonary interstitial changes, dyspnoea, tachypnoea, cough, or pyrexia<br>• Possible heart abnormalities, often manifested as rapid heartbeat, tachypnoea, swelling of ankles and soles, and feeling weak and dizzy
Other possible adverse reactions	• Diarrhoea, anaemia, cough, and proteinuria<br>• Investigation abnormal: white blood cell count decreased, and neutrophil count decreased<br>• Stomatitis: including mouth ulceration, oral mucositis, aphthous ulcer, dry mouth, oral pain, tongue ulceration, and glossodynia<br>• Eye and accessory organ disorders: including retinopathy, maculopathy, corneal exfoliation, dry eye, cataract, xerophthalmia, asthenopia, eyelid oedema, blepharochalasis, trichiasis, lacrimation increased, ocular discomfort, and foreign body sensation in eyes
Note: see the Investigator's Brochure for other adverse reactions.	

For the above adverse reactions, the following provisions are made in the protocol to minimize the risk: 1) The safety observation indicators are designed to cover the common adverse reactions of competitors, the changes requiring close attention suggested in the completed preclinical and clinical studies of aumolertinib, as well as the observation of toxicities to the heart, lung, and other vital organs. The termination criteria for subjects and the clinical trial are specified in the protocol. 2) The trial will be conducted in medical institutions with clinical trial qualifications, and experienced investigators will be selected for the clinical trial. 3) During the conduct and implementation of the trial, physicians with rich clinical experience shall regularly pay attention to and examine the health status of the patients and take corresponding treatment measures in time in case of abnormal changes. 4) The safety examinations are completed at the time points defined in the protocol, and AEs occurring in patients outside the hospital are monitored by subject diary, telephone, or in-hospital follow-ups. 5) Additional tests may be performed at any time as deemed necessary by the investigator. In case of any special event after the trial is initiated, if the ethics committee, the clinical physician, or the patient considers it necessary, the study may be immediately halted.

4.3.2 Known potential benefits

Presence of sensitive mutations in EGFR exons 18 to 24 (including exon 19 deletion and L858R mutation) renders NSCLC patients sensitive to EGFR TKI therapy. However, patients treated with the first-generation EGFR TKI will inevitably develop drug resistance in subsequent therapies, with T790M mutation being the leading cause. Aumolertinib, a novel irreversible small molecule inhibitor of EGFR, is able to selectively inhibit EGFR-sensitive mutations and T790M-resistant mutations, while having only minimal inhibitory activity against WT EGFR. Therefore, aumolertinib as first-line treatment for patients with EGFR-sensitive mutant advanced NSCLC with brain metastases may offer the following clinical benefits:

The incidence of brain metastases is approximately 20% to 30% in patients initially diagnosed with NSCLC[59]. As NSCLC progresses, approximately 50% of patients develop brain metastases. First-generation EGFR TKIs have limited efficacy against brain metastases[60]. Aumolertinib could readily penetrate the BBB with a brain-to-plasma exposure ratio > 7. At the same time, aumolertinib not only inhibits EGFR-sensitive mutations, but also inhibits T790M primary mutations and drug resistance mutations. Brain metastases are the most common cause of initial disease progression following first-generation TKI therapy. First-line treatment with aumolertinib has the potential to control, prevent, or delay the occurrence of brain metastases due to its ability to penetrate the blood-brain barrier and inhibit T790M mutations.

Available clinical data have shown that aumolertinib 110 mg as second-line treatment has a good control effect on brain metastases. The CNS response assessed by IRC using RECIST v1.1 was summarized as follows: the CNS ORR was 60.9% (95% CI: 38.5%, 80.3%), the CNS DCR was 91.3% (95% CI: 72.0%, 98.9%), the CNS mPFS was 10.8 months, and the CNS mDoR was 11.3 months. In addition, pharmacokinetic data showed that there was a linear relationship between *in vivo* drug exposure and drug dose within the dose range of 55–220 mg, and the drug was well tolerated in subjects at the highest dose of 220 mg. Therefore, increasing the dose from 110 mg to 165 mg could potentially increase systemic and cerebrospinal fluid exposure, thereby improving the treatment efficacy for brain metastases while remaining safe and tolerable.

Aumolertinib can block the formation of non-selective metabolites through structural optimization, which, coupled with its high selectivity and weak effect on wild-type EGFR, result in less toxic side effects. Current clinical studies have shown that aumolertinib is well tolerated and safe, and its main adverse reactions are changes in gastrointestinal tract, skin, and laboratory tests. The first-line use of aumolertinib can reduce the likelihood and duration of patients being exposed to other TKIs that have relatively significant side effects.

In summary, the available preclinical and efficacy and safety data of aumolertinib in patients with advanced NSCLC indicate that aumolertinib as first-line treatment is expected to have good efficacy and safety in subjects with EGFR-sensitive mutant advanced NSCLC with brain metastases who have not received any systemic treatment at the advanced stage, and high doses may offer potential benefits.

5.0 STUDY OBJECTIVES AND ENDPOINTS

5.1 Study Objectives

5.1.1 Primary objective

To evaluate the 12-month progression-free survival (PFS) rate of high-dose aumolertinib as first-line treatment of EGFR-sensitive mutant advanced NSCLC with brain metastases.

5.1.2 Secondary objectives

Secondary study objectives:

1. To evaluate other anti-tumor efficacy endpoints of high-dose aumolertinib:

Intracranial: intracranial progression-free survival (iPFS), intracranial objective response rate (iORR), intracranial duration of response (iDoR), and intracranial disease control rate (iDCR);

Systemic: objective response rate (ORR), overall survival (OS), duration of response (DoR), and disease control rate (DCR).

2. To evaluate the safety of high-dose aumolertinib.

5.1.3 Exploratory objectives

To explore the dynamic change of blood gene mapping before and after treatment and its correlation with efficacy.

5.2 Study Endpoints

5.2.1 Efficacy endpoints

The endpoints of this study will be analyzed based on the tumor response assessment by investigator per RECIST 1.1.

1. Primary endpoint:

12-month PFS rate

2. Secondary endpoints:

Intracranial: iPFS, iORR, iDoR, iDCR

Systemic: ORR, DoR, DCR, OS

5.2.2 Safety endpoints

Occurrence of AEs; occurrence of SAEs; proportion of subjects withdrawn due to AEs; changes in laboratory tests (blood biochemistry, hematology, and urinalysis); changes in vital signs, physical examination, body weight, 12-lead electrocardiogram (ECG), left ventricular ejection fraction (LVEF), ECOG performance status (PS) score, and ophthalmological examination.

5.2.3 Exploratory endpoints

Venous blood: Dynamic change of blood gene mapping before and after treatment and its correlation with efficacy.

5.3 Sample Size

This is an open-label, multi-center, prospective, single-arm clinical study. The objective is to evaluate the efficacy and safety of high-dose aumolertinib as first-line treatment in patients with EGFR-sensitive mutant advanced NSCLC with brain metastases.

We assumed that the null hypothesis of the 12-month PFS rate was 40%, and the alternative hypothesis was 60%. With a significance level of 0.1 (two-sided) and a power of 80%, 49 patients were required. Considering a dropout rate of 20%, the study neededs to enroll 63 patients.

6.0 STUDY DESIGN AND DESCRIPTION

6.1 Study Design

Study design:

This open-label, multi-center, prospective, single-arm clinical trial is intended to evaluate the efficacy and safety of high-dose aumolertinib mesylate (hereinafter referred to as "aumolertinib") in patients with epidermal growth factor receptor-sensitive mutant ($EGFRm^{+}$) advanced non-small cell lung cancer (NSCLC) with brain metastases who have not received any systemic treatment. Eligible patients will be enrolled in the high-dose aumolertinib group and

receive aumolertinib 165 mg p.o., QD. The schematic overview of the study is shown in Figure 1.

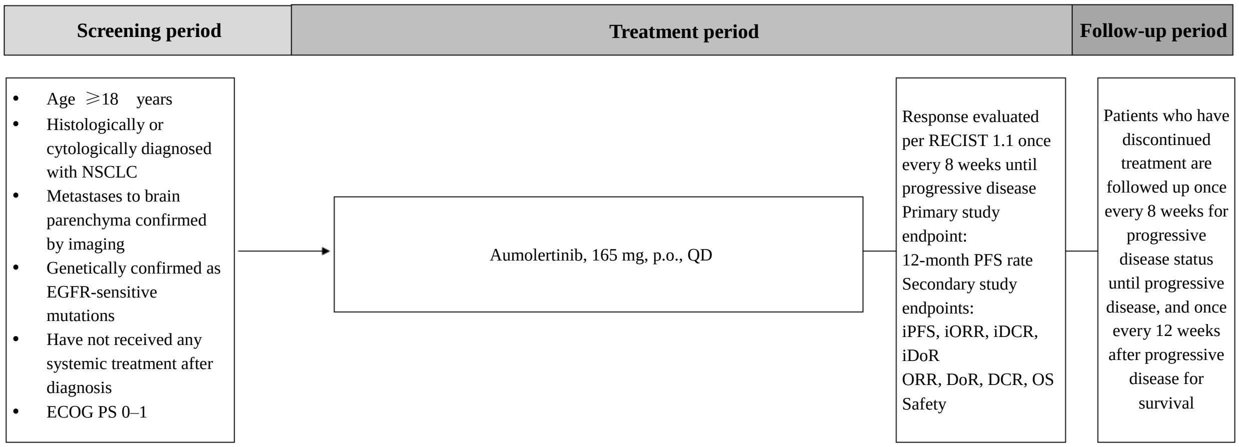

Figure 1. Schematic overview of the study

- **Screening period (D−28 to D−1)**

Inpatients or outpatients will be screened after signing the informed consent form and undergo relevant laboratory tests and assessments. Subjects who meet all the inclusion criteria and do not meet any of the exclusion criteria may be enrolled.

- **Treatment period (C1+, D1+)**

High-dose aumolertinib group: Oral administration of 165 mg aumolertinib tablets once daily (55 mg/tablet, 3 tablets/day).

Each treatment cycle consists of 28 days of continuous administration. After treatment is started, efficacy will be evaluated once every 8 weeks as per RECIST 1.1. Patients will continue treatment until progressive disease assessed by investigator per RECIST 1.1 or until the withdrawal or discontinuation criteria are met. Patients may continue to receive the treatment even if progressive disease per RECIST 1.1 occurs as long as they still benefit from the treatment as judged by investigator (see Section 12.1.3 or 9.1.3.4 for details); if it is decided to continue the treatment, the baseline and follow-up plan will not be affected by the additional courses of treatment. Once the treatment is discontinued due to lack of clinical benefit upon judgment, it cannot be started again.

- **Follow-up period**

Subjects who discontinue the treatment due to meeting the discontinuation criteria should complete the treatment discontinuation follow-up, the 28-day safety follow-up after treatment discontinuation, and survival follow-up once every 12 weeks as specified in the study plan.

Subjects without progressive disease at treatment discontinuation will be followed up once every 8 weeks until progressive disease assessed by investigator per RECIST 1.1 (intracranial, extracranial, and systemic), even if the subject has received other anti-tumor treatments; after progressive disease, the subjects will be followed up for survival once every 12 weeks as specified in the study plan.

6.2 Rationale for Study Design

This study is designed as a single-arm study of high-dose aumolertinib. The rationale is as follows: 1) There are data from other TKIs at conventional/high doses as second-line/first-line treatment in patients with EGRF-sensitive mutant NSCLC with brain metastases, and aumolertinib 110 mg as second-line treatment has a good control effect on brain metastases, but the effects of higher doses need further exploration; 2) Aumolertinib as first-line treatment not only inhibits EGFR-sensitive mutations but may also suppress potentially existing T790M resistance mutations, which could delay the onset of T790M resistance mutations and provide significant clinical benefits for patients; 3) Pharmacokinetic data showed that there was a linear relationship between *in vivo* drug exposure and drug dose within the dose range of 55–220 mg, and the drug was well tolerated in subjects at the highest dose of 220 mg. Increasing the dose from 110 mg to 165 mg could potentially increase systemic and cerebrospinal fluid exposure, thereby improving the treatment efficacy for brain metastases while remaining safe and tolerable. 4) Aumolertinib exhibits high selectivity and has a weak effect on WT EGFR, resulting in low potential toxicity. As first-line treatment, it can reduce the risk of subjects being exposed to the potential toxicity of other EGFR TKIs that have relatively higher toxicity.

6.3 Rationale for Dose Selection

A phase I dose escalation and expansion study of aumolertinib in patients with advanced NSCLC showed that oral

administration of aumolertinib once daily within the dose range of 55–260 mg was safe and well tolerated and showed preliminary anti-tumor efficacy. Previous clinical trials showed that in second-line treatment, the mDoR of subjects in the aumolertinib 220 mg group was 15.2 months, compared to 9.0 months in the 55 mg group and 12.5 months in the 110 mg group. The results suggested that 220 mg could lead to a longer duration of response. At the same time, pharmacokinetic data showed that there was a linear relationship between *in vivo* drug exposure and drug dose within the dose range of 55–220 mg. High-dose aumolertinib may improve the efficacy for brain metastases while remaining safe and tolerable. Considering the currently available efficacy, safety, and PK data of aumolertinib in clinical studies, the high dose of aumolertinib in this single-arm study is determined at 165 mg (QD).

6.4 Premature Termination or Suspension of the Study/Study Site

6.4.1 Criteria for premature termination or suspension of the study

This study will be completed as scheduled and will not be suspended or prematurely terminated unless one or more of the following criteria for suspension or premature termination is/are met:

1) Information updates or other evaluations concerning the safety or efficacy of the investigational medicinal product suggest a change in the risk-to-benefit ratio, and the changed ratio is no longer acceptable to patients participating in this study.

2) Good Clinical Practice (GCP) is seriously violated, causing the failure to achieve the primary study objectives or imperiling patient safety.

6.4.2 Criteria for premature termination or suspension of study site

If a study site (including the investigator) is in serious breach of GCP, study protocol, or contractual agreement and is unable to ensure adequate conduct of this study, the site will be prematurely terminated or suspended.

6.4.3 Procedure for premature termination or suspension of the study/study site

If the sponsor, Institutional Review Board (IRB)/Independent Ethics Committee (IEC), or regulatory authorities choose to terminate or suspend the study/study site, the sponsor will provide a specific procedure for premature termination or suspension. Such procedure will be executed at the corresponding study site during termination or suspension of the study/study site.

7.0 SCREENING OF SUBJECTS

7.1 Inclusion Criteria

A subject must meet all the following inclusion criteria in order to be enrolled in this study:

1. Age ≥18 years old.
2. Histologically or cytologically confirmed NSCLC, which is confirmed by imaging as advanced NSCLC with brain metastases (including stage IV patients who are newly diagnosed or have relapsed after previous surgical treatment; according to the AJCC 8th edition lung cancer staging criteria).
3. Confirmed as EGFR-sensitive mutations in tumor tissue samples or blood samples by local laboratory (including exon 19 deletion or L858R, either existing alone or co-existing with other EGFR site mutations). If tumor tissues are available, the tumor tissue is recommended for testing; if tumor tissues are not available or the patient cannot undergo tissue biopsy, blood samples may be tested.
4. Have not received any systemic treatment after the diagnosis of NSCLC with metastases to brain parenchyma. For patients who have received local treatment, the lesion within the scope of the local treatment cannot be used as the target lesion unless the lesion has progressed.
5. Patients with at least 1 extracranial target lesion (measurable lesions that have not undergone local treatment such as irradiation or have shown definitive after local treatment, with a baseline longest diameter ≥ 10 mm (or ≥ 15 mm for lymph nodes)) and at least 1 intracranial lesion (with a baseline longest diameter ≥ 5 mm).
6. The brain condition is stable or that were controlled with anticonvulsants or dexamethasone at a maximum dose of 5 mg daily before the study treatment.
7. An Eastern Cooperative Oncology Group (ECOG) performance status score of 0 or 1, with no worsening over at least 2 weeks before study treatment, and a minimum expected survival of 3 months.
8. Female patients of childbearing potential are willing to take appropriate contraceptive measures and should not breastfeed from signing the informed consent to 6 months after the last dose of study treatment; male patients are willing to take barrier contraception (i.e., condoms) from signing the informed consent to 6 months after the last dose study treatment.
9. Female patients of childbearing potential must have a negative serum or urine HCG test within 7 days before enrollment in the study, and they must be non-lactating.
10. The subject voluntarily participates and signs the informed consent form in writing.

7.2 Exclusion Criteria

A subject will not be eligible for inclusion in this study if any of the following criteria apply:

1. Treatment with any of the following:
   a. Previously received treatment with tyrosine kinase inhibitors against EGFR (such as erlotinib, gefitinib, icotinib, afatinib, osimertinib, and aumolertinib);
   b. The patient has undergone major surgery (such as craniotomy, thoracotomy, or laparotomy) within 4 weeks before the first dose of the investigational medicinal product, or undergone minor traumatic surgery (biopsy, bronchoscopy, and thoracic drainage) within 7 days before the first dose of the investigational medicinal product. Major surgeries are defined as Grade 3 and Grade 4 surgeries specified in the "Administrative Measures for Clinical Application of Medical Technology" implemented from Nov. 1, 2018 in Appendix H;
   c. Patients who have received local radiotherapy within 2 weeks before the first dose of the investigational medicinal product; patients who have received more than 30% of bone marrow irradiation (see Appendix I for the calculated area of bone marrow) or extensive radiotherapy within 4 weeks before the first dose of the investigational medicinal product; patients who have received whole brain radiotherapy for this disease before enrollment;
   d. Recurrence within 6 months after adjuvant or neoadjuvant treatment for early lung cancer; if there is both neoadjuvant therapy and adjuvant therapy, the adjuvant treatment time will be used for calculation;
   e. Within 14 days before the first dose of the investigational medicinal product, traditional Chinese medicines and their preparations with anti-tumor effect or adjuvant anti-tumor effect have been used (see Appendix E for the list of drugs);
   f. Presence of pleural/abdominal effusion requiring clinical intervention (patients who do not require drainage of the effusion or are stable for more than 2 weeks after drainage of the effusion are eligible); presence of pericardial effusion (patients with small amount of pericardial effusion who are stable for 2 weeks or more are eligible). If anti-tumor drugs are used locally (e.g., via intrapleural infusion) during drainage, patients are only eligible with a washout of at least 5 half-lives or 21 days (whichever is shorter) prior to the first dose of study treatment;
   g. Previously treated with drugs that are strong inhibitors or strong inducers of CYP3A4, or treated with CYP3A4 sensitive substrate drugs with a narrow therapeutic window, within 7 days prior to the first dose of investigational medicinal product; or requiring to continue the treatment with these drugs during the study (see Appendix E for the list of these drugs);
   h. Receiving drugs known to prolong the QT interval or that may cause torsade de pointes, or requiring to continue the treatment with these drugs during the study (see Appendix E for the list of drugs and washout times);
   i. Having participated in another clinical trial within 4 weeks or been still within 5 half-lives of the investigational medicinal product in another clinical trial before the first dose of this investigational medicinal product, whichever is longer (except for screening failure).
2. Histologically or cytologically confirmed mixed SCLC and mixed NSCLC, large cell neuroendocrine carcinoma and sarcomatoid carcinoma.
3. At the beginning of study treatment, those with residual toxicity from prior anti-tumor treatment greater than CTCAE Grade 1 that cannot be alleviated, with the exception of hair loss and Grade 2 neurotoxicity from prior anti-tumor treatment. History of intracranial hemorrhage not related to tumor.
4. History of other primary malignant tumors, except for the following:
   a. Radically treated malignancies that have been inactive for ≥ 5 years prior to study enrollment and have a very low risk of recurrence;
   b. Adequately treated non-melanoma skin cancer or lentigo maligna without evidence of disease recurrence;
   c. Adequately treated carcinoma *in situ* without evidence of disease recurrence.
5. Diagnosis of meningeal metastasis through clinical symptoms or imaging or cerebrospinal fluid, or brain parenchymal metastasis combined with meningeal metastasis.
6. Patients who are allergic to MRI contrast agent gadolinium or who cannot tolerate MRI examinations (such as patients with pacemakers or metals in the body).
7. Any serious or poorly controlled systemic disease, such as poorly controlled hypertension, active bleeding diathesis, or active infection, as judged by the investigator. There is no need to check for chronic diseases.
8. Serious gastrointestinal dysfunction that may affect drug intake, transport, or absorption, such as inability to take oral drugs, uncontrollable nausea or vomiting, extensive gastrointestinal resection history, uncured recurrent diarrhea, atrophic gastritis, uncured gastric diseases requiring long-term use of acid-reducing proton pump inhibitors, Crohn's disease, ulcerative colitis, etc.
9. Hepatic encephalopathy, hepatorenal syndrome, or cirrhosis.

10. Any of the following cardiac findings:
    a. Mean QT interval corrected by Fridericia (QTcF) > 470 msec from 3 repeated measurements of electrocardiogram (ECG) at rest;
    b. Any clinically significant abnormalities in rhythm, conduction, or ECG morphology determined by the investigator (e.g., complete left bundle branch block, third-degree atrioventricular block, second-degree atrioventricular block, and PR interval > 250 msec), as indicated by ECG at rest;
    c. Presence of any factor that increases the risk of QTc prolongation or arrhythmic events, such as cardiac failure, hypokalemia, congenital long QT syndrome, family history of long QT syndrome, or unexplained sudden death of immediate family members under 40 years of age, or any concomitant medications known to prolong QT interval;
    d. Left ventricular ejection fraction (LVEF) < 50%.
11. Insufficient bone marrow reserve or organ function, meeting any of the following laboratory test limits (no corrective treatment within 1 week before blood collection for laboratory tests):
    a. Absolute neutrophil count < 1.5×10^9/L;
    b. Platelet count < 100×10^9/L;
    c. Hemoglobin < 90 g/L (< 9 g/dL);
    d. Alanine aminotransferase > 2.5 × upper limit of normal (ULN) in the absence of demonstrable liver metastases; alanine aminotransferase > 5 × ULN in the presence of liver metastases;
    e. Aspartate aminotransferase > 2.5 × ULN in the absence of demonstrable liver metastases; aspartate aminotransferase > 5 × ULN in the presence of liver metastases;
    f. Total bilirubin > 1.5 × ULN in the absence of demonstrable liver metastases, or total bilirubin > 3 × ULN in the presence of Gilbert's syndrome (unconjugated hyperbilirubinemia) or liver metastases;
    g. Creatinine > 1.5 × ULN and creatinine clearance < 50 mL/min (calculated by Cockcroft-Gault formula); creatinine clearance should be determined only when creatinine is > 1.5 × ULN;
    h. Serum albumin (ALB) < 28 g/L;
12. Female subjects who are pregnant and lactating or plan to become pregnant during the study period.
13. History of interstitial lung disease, drug-induced interstitial lung disease, radiation pneumonitis requiring steroid therapy, or any evidence of clinically active interstitial lung disease.
14. History of hypersensitivity to any active or inactive ingredients of aumolertinib or drugs of similar chemical structure or the same class as aumolertinib.
15. Any severe or uncontrolled ocular diseases (particularly severe dry eye syndrome, keratoconjunctivitis sicca, severe exposure keratitis, or other conditions that may increase the risk of epithelial damage) that may increase the safety risks of patients as judged by the physician; or patients who have any eye disorders that require surgical treatment or are expected to require surgical treatment during the study period.
16. Patients who may have poor compliance with the procedures and requirements of the study as judged by the investigator, such as patients who have a clear history of neurological or mental disorders (including epilepsy or dementia), or patients who are currently suffering from mental disorders.
17. Patients with any conditions that may jeopardize patient safety or interfere with study assessments as judged by the investigator.

7.3 Recruitment and Screening Number

Prior to screening, each subject recruited will be assigned a screening number in the order of time signing the informed consent form. Once assigned to a subject, the screening number can no longer be reused. If a subject is screened again, a new screening number will be assigned.

Patients, who are not enrolled because they do not meet the inclusion criteria or they meet any of the exclusion criteria after they sign the informed consent form, are considered "screen failures". For patients who fail screening, the active collection of patient information will end when the screen failure status is confirmed. For subjects for whom "screen failure" is confirmed, the screening process will not be initiated anew, unless any of the following conditions are met:

Condition 1: Due to a revision to the study protocol, the patient meets the eligibility criteria for participating in the clinical study.

Condition 2: Due to a change in the status of the patient, the eligibility criteria, which the patient previously failed to meet, will no longer cause the patient to fail screening once again.

When being re-screened, the patient must sign a new informed consent form and will be assigned a new screening number. However, the patient will be re-screened once only. If a previous test has been conducted beyond the protocol-defined time window, the test needs to be repeated. Echocardiogram and per-protocol tumor imaging examinations performed within 28 days prior to randomization need not be conducted anew.

7.4 Enrollment Status

This is a prospective, single-arm, open-label, multi-center clinical trial. It will not involve randomization, and study sites will compete to enroll subjects in the treatment group (aumolertinib 165 mg).

7.5 Procedures for Managing Wrongly Enrolled Subjects

Subjects not meeting the eligibility criteria of the study should not participate in this study. If subjects not meeting the eligibility criteria are enrolled, study treatment should not be provided. If treatment has been started, the investigator should immediately perform a comprehensive assessment and decide whether to discontinue the treatment based on the assessment results. If treatment has been started for a subject not meeting the eligibility criteria, the subject should be withdrawn from the study. This process must be fully documented in the medical record.

7.6 Limitations

1) Women of childbearing potential should use reliable contraceptive methods from the signing of the informed consent form until 6 months after the end of the study treatment. Acceptable contraceptive methods include abstinence, tubal ligation, oral or transdermal contraceptives, copper intrauterine device, and vasectomized partner. All methods of hormonal contraception should be used in conjunction with the use of a condom by male partners of patients.

2) Male patients should use barrier contraception (i.e., use of a condom) from signing the informed consent form until 6 months after the end of study treatment. Male patients should avoid sperm donation from the start of administration until 6 months after the end of study treatment. A male patient who wishes to become a father are advised to freeze their sperm samples prior to the start of study treatment.

3) When patients who wear contact lenses receive aumolertinib: In case of the initial occurrence of CTCAE Grade ≥ 3 ocular events, the patients must discontinue wearing contact lenses until at least 1 week after permanent discontinuation of aumolertinib; in case of the initial occurrence of mild to moderate ocular events (CTCAE Grade ≤ 2), the patients must discontinue wearing contact lenses until at least 1 week after symptom disappearance; if the patients develop ocular events (any grade) once again, they must discontinue wearing contact lenses until at least 1 week after permanent discontinuation of aumolertinib. Patients must not, at their own discretion, use any eye drops or ointments for ocular symptoms during the study period and until 1 week after permanent discontinuation of aumolertinib, unless otherwise permitted by the investigator. In case of any concerns, patients should consult the study site in a timely manner.

5) It is advised to monitor low-density lipoprotein cholesterol, blood creatine phosphokinase, and muscle symptoms when statins are co-administered with aumolertinib. If subjects experience any potentially related AEs that suggest myotoxicity, including unexplained muscle pain, tenderness, or weakness, particularly accompanied by discomfort or pyrexia, statins should be discontinued, blood creatine phosphokinase levels should be measured, and further examinations and management should be performed as appropriate.

6) Prothrombin time or international normalized ratio (INR) should be monitored in subjects receiving warfarin.

7) Patients who develop corneal ulcer will not be permitted to restart receiving the study treatment.

8) If new or worsening pulmonary symptoms (e.g., dyspnea) are observed or an imaging abnormality suggests the presence of interstitial lung disease, suspension of study treatment is recommended and the sponsor and funder should be notified. The investigator is strongly advised to perform a thorough diagnostic workup on patients to rule out other causes such as lymphangitic carcinomatosis, infection, allergy, edema due to cardiac disease, or pulmonary hemorrhage. The diagnosis of interstitial lung disease should be considered if confirmed by high-resolution CT (HRCT) scans and other causes of respiratory symptoms have been ruled out, and study treatment should be permanently discontinued. In the absence of a diagnosis of interstitial lung disease, treatment may be resumed after a comprehensive safety assessment by the investigator.

7.7 Concomitant Medications and Treatments

All medications and indications used from 28 days before the start of study treatment to 28 days after treatment discontinuation will be recorded in the electronic case report form (eCRF). Only information on anti-tumor therapies will be recorded in the eCRF after the 28-day follow-up.

Concomitant medications/treatments are other medications/treatments administered at the investigator's discretion and based on the subject's interest.

7.7.1 Permitted concomitant treatments

Palliative and supportive care for the management of underlying diseases and symptoms are permitted during the course of the study for the subject's health. Patients who routinely receive medications other than antineoplastic agents should continue receiving the medications during the study period if medically feasible.

For concomitant treatments and medications used within 28 days before enrollment and during the study, the dose, frequency, route of administration, and date should be recorded in the CRF. In case of any AE, the subject should be

closely observed, and if necessary, active symptomatic treatment should be given, and the drug used should be recorded and described.

7.7.2 Other anti-tumor/anti-cancer or investigational medicinal products

Other anti-tumor treatments, such as chemotherapy, molecular targeted therapy, immunotherapy, biological therapy, radiotherapy, and traditional Chinese medicine for anti-tumor indications (see Appendix D for the list of traditional Chinese medicines for anti-tumor indications), are not allowed before any progressive disease (systemic, intracranial, or extracranial) unless the patient receives radiotherapy due to pain from bone metastases or discontinues treatment before study treatment completion due to reasons other than progressive disease, in which case other anti-tumor treatments are allowed. Patients may receive corticosteroids and/or bisphosphonates or denosumab for the treatment of bone metastases. After any progressive disease (systemic, intracranial, or extracranial), subsequent anti-tumor treatment should be given with reference to Section 12.1.3 or 9.1.3.4.

During the subsequent treatment with aumolertinib, concomitant treatment with other systemic anti-tumor therapies is not permitted. However, concomitant local therapy may be considered if a thorough assessment indicates that it would be beneficial to the patient.

7.7.3 Prohibited medications

Prophylactic treatments for diarrhea, nausea, and vomiting must not be provided prior to the first dose of aumolertinib, but may be provided after the first dose.

After enrollment and during the oral administration of aumolertinib, the use of potent CYP3A4 inhibitors, inducers, or drugs with a narrow therapeutic window as sensitive CYP3A4 substrates is prohibited (see Appendix D for the list of drugs) until 28 days after treatment discontinuation. Other medications that are substrates, inhibitors, or inducers of CYP3A4, traditional Chinese herbal supplements, and foods (e.g., pitaya, citrus fruits such as grapefruit, tangerine, and orange, mango, and juice containing the above fruits) should be used with caution.

Patients will be permitted to receive blood transfusion therapy at any time during the study period.

Patients are prohibited from receiving drugs that are known to prolong the QTc interval or may cause torsade de pointes (see Appendix D for the list of drugs) after enrollment and during study treatment until 28 days after the treatment discontinuation. Other drugs that may prolong the cardiac QT interval should be used with caution.

If AEs should be treated when clinically needed, patients should receive non-prohibited medications when possible, and the safety of patients should be closely monitored if prohibited medications are necessarily used and such use is approved.

7.7.4 Glucocorticoids

Long-term systemic use of glucocorticoids is not recommended for supportive care purposes. Short-term use of glucocorticoids may be permitted for the treatment of non-autoimmune diseases (e.g., delayed allergic reactions caused by contact allergens) in individual subjects following discussion with the sponsor.

Glucocorticoids are permitted for emergency use, topical application, inhalation via spray, eye drops, or topical injection, and can be used by subjects requiring long-term inhalation of glucocorticoids at doses that do not induce systemic dose-response effects for treatment of asthma or chronic obstructive pulmonary disease. For patients with baseline symptoms of brain metastases, the use of dexamethasone at a maximum dose of 5 mg daily for symptom control is permitted. Use of systemic glucocorticoids ($\leq$ 10 mg/day prednisone or its equivalent) at physiologic replacement doses (e.g., adrenal replacement steroid dose) for treatment of diseases such as adrenal or pituitary insufficiency is permitted.

Prophylactic use of glucocorticoids according to the package insert is permitted to prevent allergic reactions (e.g., pretreatment prior to intravenous contrast agent administration).

7.7.5 Surgery (non-antineoplastic purpose)

The rationale and necessity for any surgery performed during the study period should be documented. For patients who must undergo a surgery during study treatment, the investigator should determine the necessity to interrupt the investigational medicinal product and the time of interruption, depending on the clinical assessments of wound healing and postoperative rehabilitation.

7.8 Treatment Discontinuation and Withdrawal from Study

7.8.1 Treatment discontinuation

Treatment discontinuation means that the subject no longer receives the study treatment due to the following reasons after the start of study treatment, but is not withdrawn from the study and will complete subsequent follow-ups as scheduled.

1. Progressive disease (refer to 2 in case of free medication)
2. Disease progression after free medication
3. Adverse event
4. Poor compliance

5. Major protocol violation
6. Patients are no longer eligible to continue with the study treatment as believed by the investigator or funder (e.g., psychiatric disorders or social status that may interfere with subject's rights, safety, cooperation, and participation in this study)
7. Patients request for treatment discontinuation but agree to continue to be followed up
8. Patients withdraw the informed consent
9. Loss to follow-up
10. Pregnancy
11. Study termination or site closure
12. Patients who develop corneal ulcer or interstitial pneumonia should permanently discontinue the treatment

Every effort should be made to obtain information of patients who discontinue study treatment, including information concerning EOT follow-up and the safety follow-up thereafter. The primary reasons for study treatment discontinuation should be documented on the corresponding eCRF. A patient's request for withdrawal from the study at the same time must be documented in the source documents, with the investigator's signature affixed.

In addition, patients who discontinue study treatment due to reasons other than PD should undergo tumor assessments as scheduled until documented PD, unless the patient withdraws from the study due to any of the reasons described in 7.8.2.

7.8.2 Withdrawal from study

Patients may voluntarily withdraw from the study at any time. Reasons for withdrawal from the study may include but are not limited to:

- Withdrawal of informed consent
- Study termination or site closure
- Loss to follow-up
- Death

Every effort should be made to obtain information of patients who withdraw from the study. The primary reasons for withdrawal from the study should be documented on the corresponding medical record. A patient's request for withdrawal from the study must be documented in the source documents, with the investigator's signature affixed.

If a patient withdraws from the study, the study personnel may obtain their survival status-related information via public information sources.

8.0 MANAGEMENT OF INVESTIGATIONAL MEDICINAL PRODUCTS

8.1 Investigational Medicinal Products

8.1.1 Dosing regimen

The investigational medicinal product will be administered orally once daily according to the dosing regimen in Table 2.

Table 2. Dosing regimen

	**High-dose aumolertinib group**
Dose	Aumolertinib, 165 mg/day
Drug	Aumolertinib active drug, 55 mg/tablet, 3 tablets
Dosage and route of administration	p.o., QD

Subjects are advised to take the investigational medicinal product as whole tablets with a sufficient quantity of room temperature water and avoid eating from 1 h before dosing to 2 h after dosing.

The administration time should be consistent every day to maintain a 24-hour interval. If a subject fails to take a dose at a scheduled time point and the time window is exceeded by ≤ 12 h, the subject may make up the missed dose; if the time window is exceeded by > 12 h, the subject should not make up the missed dose and should take the next dose at the next scheduled time point.

If a subject vomits after a dose, the subject should not make up the dose and should receive the next dose at the next scheduled time point. Any changes related to dosing should be recorded.

8.1.2 Dosage form, manufacturing, packaging, and labeling

In this protocol, the investigational medicinal products will be uniformly packaged, numbered, and provided to each study site. The drug label will contain information including but not limited to protocol number and clinical trial medication warning.

8.1.3 Storage

All investigational medicinal products must be stored in an appropriate and secure place until they are used or returned to the funder or its designee for destruction. The investigational medicinal products should be dispensed to patients participating in the study only. All investigational medicinal products must be stored in sealed containers below 30 °C.

8.2 Dispensing Procedures of Investigational Medicinal Products

Subjects are assigned screening numbers in the order of their visits. After relevant examinations, eligible subjects are allocated to the high-dose aumolertinib group, and the corresponding investigational medicinal products are then dispensed. The dispensing and recovery of each drug should be timely documented on a dedicated record sheet. The drug manager should accurately fill out the drug dispensing/recovery registration form and properly keep all investigational medicinal products.

8.3 Medication Compliance

Subjects should take the investigational medicinal product orally once daily according to the trial requirements. Unused drugs should be documented in detail, with reasons explained. The investigator should check the medication record against the remaining investigational medicinal products to verify compliance. If any compliance deviation occurs, appropriate explanations should be provided.

8.4 Management of Investigational Medicinal Products

All investigational medicinal products should be stored in a safe and qualified place, and there should be a designee responsible for managing the registration, dispensing, and recovery records of investigational medicinal products.

1) Drug handover sheet: Upon receipt of investigational medicinal products, the investigator or designee must check the investigational medicinal products against the packing list. The investigator or designee should ensure that the quantity of the investigational medicinal products is correct and packaging is in good condition, and should confirm the receipt of the investigational medicinal products under labeled storage conditions. If the quantity and conditions are acceptable, the investigator or designee and the CRA should acknowledge receipt of the investigational medicinal products. In case of any discrepancy between the information in the packing list and the information of investigational medicinal products actually received, the funder must be contacted to resolve such discrepancy. The packing list should be archived in the investigator site file.

2) Drug dispensing/recovery registration form: The initials of subjects, subject number, date of dispensing, quantity dispensed, date of recovery, quantity recovered, and signatures of the persons who dispense and recover drugs should be documented. The sponsor and funder must be immediately notified of any dispensing errors or discrepancies.

3) Drug temperature record form: Unused drugs should be stored as per storage requirements since drug handover, and the drug storage temperature should be recorded daily.

4) Drug recovery record form: Upon completion of the study, all remaining drugs will be uniformly recovered and disposed of by the sponsor and funder, with the signatures of the investigator and CRA affixed for confirmation.

5) Drug destruction record form: The funder should destroy and document all remaining investigational medicinal products recovered.

To ensure the proper use of investigational medicinal products, the investigator should retain the records of transportation, inventory, dispensing to and use of each patient, and recovery of all of the above investigational medicinal products, and the records of returning the remaining IMPs to the sponsor and funder or the designee. The copies of the source documents may be sent to the sponsor and funder or the designee at request.

The investigator will be responsible for the receipt, storage, and dispensing of investigational medicinal products during the study period, and his/her responsibilities include but are not limited to:

- Frequently confirm whether the actual inventory matches the documented one.
- Confirm and complete the log of drug batches.
- Confirm the accurate records of all used and unused drugs.
- Confirm that mandatory fields are completed in an accurate and legible manner.

The sponsor and funder or the designee will notify the investigator of the expiry date or re-test date of the study materials during the study period. Upon receipt of the notification concerning the expiry date of study materials, the study site must complete all procedures described in the notification, such as sorting out and returning expired study materials to the sponsor and funder or the designee for destruction.

9.0 STUDY PLAN AND PROCEDURES

9.1 Study Procedures

Below are study procedures and data to be collected. Each patient will be assessed by the same investigator or study site personnel when possible at each step. See Appendix B for the overall follow-up schedule.

The subject must read and voluntarily sign the ICF approved by the site's ethics committee prior to the initiation of

any study procedures. All study procedures should be performed within the time windows indicated in the schedule of activities.

9.1.1 Screening period (D−28 to D0)

After the ICF is signed, the screening assessment will begin, lasting for a maximum of 28 days.

- Subjects must provide full informed consent and sign the ICF prior to the start of any study procedures.
- Collection of lung cancer diagnosis data (including but not limited to): time of first diagnosis of NSCLC, pathological diagnosis (if any), tumor stage at that time, time of first diagnosis of metastatic NSCLC, pathological diagnosis, and current tumor stage
- Collection of previous history of anti-tumor therapy for NSCLC: date of surgery (if any), surgical method (if any), start and end time of anti-tumor drugs, treatment regimen, dose, treatment outcome, and remaining toxicities
- Review of existing positive EGFR mutation test results (test reports issued within 3 months before screening are deemed valid); for patients without acceptable EGFR mutation test results, tumor tissue sample and/or blood sample should be collected for EGFR mutation test
- Collection of demographic data (including date of birth or age, sex, ethnicity, and smoking status)
- Collection of comprehensive medical history information (including past medical history, current medical history/concomitant disease, treatment history, history of surgery, and history of allergy)
- Body height measurement
- Body weight measurement
- Comprehensive physical examination
- Vital signs measurement (blood pressure, pulse, respiratory rate, and body temperature)
- ECOG PS score
- Hematology test
- Blood biochemistry test
- Urinalysis
- 12-lead ECG examination
- Echocardiography (results obtained within 28 days prior to enrollment are acceptable and are not subject to the timing of providing informed consent)
- Ophthalmological examination: Ophthalmological examination should be performed as clinically indicated. The investigator will decide whether to perform an ophthalmological examination based on the patient's condition.
- Blood pregnancy test for women of childbearing potential (within 3 days before enrollment)
- Baseline tumor assessment: Contrast-enhanced CT of the chest, contrast-enhanced CT of the abdomen (including liver and adrenal glands), contrast-enhanced MRI (preferred)/contrast-enhanced CT of the head, bone scan, and imaging examination of other sites with indications for metastasis. The slice thickness in a CT scan must be ≤ 5 mm. The results of contrast-enhanced CT of the chest, contrast-enhanced CT of the abdomen (including the liver and adrenal glands), and contrast-enhanced MRI (preferred)/contrast-enhanced CT of the head obtained within 28 days prior to the enrollment are acceptable. Bone scan results obtained within 3 months prior to the enrollment are acceptable and are not subject to the time of providing informed consent. Patients who are allergic to contrast agents should undergo a plain chest CT and should have MRI scans of the abdomen and head. If suspected metastases are found on a bone scan, bone metastases must be confirmed by CT, MRI, or X-ray. If a PET-CT scan (slice thickness must be ≤ 5 mm) is conducted within 3 months prior to enrollment, a bone scan will be unnecessary.
- Evaluation of inclusion and exclusion criteria
- Recording of AEs and concomitant medications (concomitant medications started from 4 weeks before enrollment)

It is recommended that the screening process should start with the confirmation of EGFR mutation status.

If a laboratory test value is abnormal in the screening period and no corrective treatment is needed as judged by the investigator, a re-test may be performed within 1 week; if a re-test is performed, the re-test results should be used as the basis for judgment against the inclusion/exclusion criteria. If the corrective treatment is needed as judged by the investigator, the eligible test results obtained after corrective treatment should remain stable for at least 1 week and meet the requirements for the time window in the screening period and the sponsor's requirements.

9.1.2 Treatment period

9.1.2.1 Treatment regimen

One cycle of treatment is defined as 28 days of oral aumolertinib 165 mg once daily. It is recommended to start the

study treatment as soon as possible after enrollment. Patients will continue the study treatment until progressive disease assessed by investigator per RECIST 1.1. Or, patients may continue the study treatment despite progressive disease if they can still obtain clinical benefits as judged by the investigator (see Section 12.1.3 or 9.1.3.4 for details).

9.1.2.2 Treatment visits

Treatment visits (C1, C2)

On the first day of the first 2 treatment cycles, treatment period visits will be conducted. The specific visit procedures are as follows:

- Drug dispensing, comprehensive physical examination, body weight measurement, ECOG PS scoring, vital signs measurement, laboratory tests (clinical biochemistry, hematology, and urinalysis), and 12-lead ECG are conducted on C1D1 and C2D1 (Drugs of 1 treatment cycle or 2 treatment cycles are dispensed on C1D1 or C2D1).
- Venous blood sampling: Blood samples should be collected within 7 days pre-administration and post-administration on C1D1 and C2D1.
- Ophthalmological examinations will be performed as clinically indicated throughout the treatment period, and the examination items will be determined by the investigator based on the subject's condition
- Echocardiography will be performed when clinically indicated
- Response evaluation in solid tumors will be performed as per RECIST 1.1 on C2D1: Tumor assessment will include enhanced CT (preferred) or enhanced MRI of the chest and abdomen (including liver and adrenal glands), enhanced MRI (preferred)/enhanced CT of the head, and the sites of positive lesions (enhanced CT/enhanced MRI) confirmed at baseline. The imaging method should be consistent with that at baseline whenever feasible (unless there are contraindications or force majeure factors)
- AEs and concomitant medications will be recorded

Treatment visits (C4+, D113+)

The treatment visits starting from the 4th treatment cycle should be carried out according to the following procedures:

- Drug dispensing (drug for 2 treatment cycles), comprehensive physical examination, body weight measurement, ECOG PS scoring, vital signs measurement, laboratory tests (clinical biochemistry, hematology, and urinalysis), and 12-lead ECG will be performed once every 8 weeks
- Ophthalmological examinations will be performed as clinically indicated throughout the treatment period, and the examination items will be determined by the investigator based on the subject's condition
- Echocardiography will be performed when clinically indicated
- Response evaluation in solid tumors will be performed as per RECIST 1.1 once every 8 weeks
- AEs and concomitant medications will be recorded

9.1.3 Follow-up period

9.1.3.1 EOT follow-up

The end-of-treatment (EOT) follow-up refers to the visit procedures to be completed when a subject completely discontinues the study treatment, which is required to be completed within 7 days after the last dose. The procedures include the following:

- Comprehensive physical examination, body weight measurement, ECOG PS scoring, vital signs measurement, laboratory tests (clinical biochemistry, hematology, and urinalysis), 12-lead ECG, echocardiography, and ophthalmological examination (when clinically indicated)
- Response evaluation in solid tumors as per RECIST 1.1
- AEs and concomitant medications will be recorded

At the EOT visit, if there are safety examination results (comprehensive physical examination, body weight measurement, ECOG PS scoring, vital signs measurement, laboratory tests, and 12-lead ECG examination) within 7 days before the visit, repeated examination will not be required; if there are RECIST response evaluation results and echocardiography results within 4 weeks before the visit, repeated evaluation/measurement will not be required.

9.1.3.2 Safety follow-up (28-day follow-up)

Safety follow-up refers to visit procedures to be completed during the 28 days after treatment discontinuation (discontinuation of investigational medicinal product administration) to follow up on the ongoing and new AEs. AEs, for which outcomes are not available, should be followed up until outcomes are available or AEs are stable, which is not subject to the 28-day follow-up period. Only investigational medicinal product/procedure-related SAEs will be collected thereafter. The procedures include the following:

- Documentation of AEs (new AEs or follow-up information of AEs ongoing at treatment discontinuation)
- Documentation of concomitant medications (all concomitant medications including anti-tumor treatment)

9.1.3.3 Progression follow-up

Progression follow-up refers to the visit procedures to be completed for subjects who discontinue treatment for reasons other than progressive disease. The subjects will undergo assessment per RECIST 1.1 once every 8 weeks (relative to the date of enrollment). The procedures include the following:

- Response evaluation in solid tumors once every 8 weeks until progressive disease assessed by investigator per RECIST 1.1 (all systemic, intracranial, and extracranial)
- Documentation of concomitant medications: Only details of subsequent anti-tumor treatments will be documented until progressive disease (all systemic, intracranial, and extracranial)
- Subjects who experience progressive disease before completing the 28-day safety follow-up should continue undergoing the safety follow-up until 28 days after treatment discontinuation

9.1.3.4 Follow-up of patients after any disease progression

At the time of disease progression assessment, 3 sets of assessments will be performed in this study: systematic assessment, intracranial assessment, and extracranial assessment. During the overall assessment, extracranial and intracranial target lesions (no more than 5 target lesions in the whole body and at least one target lesion in the skull) will be selected according to RECIST 1.1, and then the selected intracranial and extracranial lesions will be subjected to separate intracranial and extracranial response evaluation according to the RECIST 1.1. There is no need to select additional intracranial or extracranial target lesions. See Table 3 for systemic, intracranial, and extracranial disease progression, subsequent anti-tumor therapies, and recommendations for data collection.

Table 3. Determination of disease progression, subsequent anti-tumor therapies, and data collection

<table>
<tr><th>Systematic review</th><th>Intracranial lesions</th><th>Extracranial lesions</th><th>Subsequent treatment</th><th>Subsequent anti-tumor evaluation</th></tr>
<tr><td>PD</td><td>PD</td><td>PD</td><td>Treatment discontinuation</td><td rowspan="12">Continue evaluation until progressive disease as per RECIST 1.1 (all intracranial, extracranial, and systemic)</td></tr>
<tr><td rowspan="2">PD</td><td rowspan="2">PD</td><td rowspan="2">Non-PD</td><td>Treatment discontinuation</td></tr>
<tr><td>Continue aumolertinib + brain local treatment</td></tr>
<tr><td rowspan="2">PD</td><td rowspan="2">Non-PD</td><td rowspan="2">PD</td><td>Treatment discontinuation</td></tr>
<tr><td>Continue aumolertinib + extracranial local treatment (oligometastasis)</td></tr>
<tr><td>Non-PD</td><td>PD</td><td>PD</td><td>Treatment discontinuation</td></tr>
<tr><td rowspan="2">Non-PD</td><td rowspan="2">PD</td><td rowspan="2">Non-PD</td><td>Treatment discontinuation</td></tr>
<tr><td>Continue aumolertinib + brain local treatment</td></tr>
<tr><td rowspan="2">Non-PD</td><td rowspan="2">Non-PD</td><td rowspan="2">PD</td><td>Treatment discontinuation</td></tr>
<tr><td>Continue aumolertinib + extracranial local treatment (oligometastasis)</td></tr>
</table>

9.1.3.5 Survival follow-up

For patients who have discontinued the study treatment, the patient or their family must be contacted once every 12 weeks from the time of treatment discontinuation to obtain survival information until their withdrawal from the study (withdrawal of informed consent, study termination or site closure, loss to follow-up, or death). In addition, details of subsequent anti-tumor treatment regimens and unresolved AEs must be collected, regardless of the date of last contact. The procedures include the following:

- Follow-up once every 12 weeks to document the details of subsequent anti-tumor treatments
- Follow-up once every 12 weeks to record subsequent response/progression data
- Follow-up once every 12 weeks to document the survival status

9.2 Unscheduled Visits/Tests

The investigator may perform unscheduled visits or examinations depending on the subject's condition, especially if the subject experiences an AE. An unscheduled visit and corresponding unscheduled examinations will be recorded in the eCRF, along with the reason for the unscheduled visit (or examination).

10.0 STUDY EVALUATION

10.1 Study-Specific Procedures

The requirements and instructions for each procedure and test/assessment indicator in the study schedule are detailed below.

10.1.1 Informed consent process

Informed consent must be obtained before subject enrollment and initiation of study procedures. Informed consent for participation in the study and informed consent for the sample collection should be included in the informed consent form at the time of enrollment.

10.1.2 Screening number assignment

Each patient will be assigned a unique screening number when informed consent is provided. The screening numbers of subjects who fail screening or withdraw from this study should not be reused.

10.1.3 Screening and enrollment

Each recruited subject must obtain the informed consent form before any study procedures are carried out, and then the subject will start screening.

Tumor assessments and other clinical data obtained prior to informed consent may not be repeated if they are completed within the time frame specified in the protocol. It is recommended that the screening process should start with the confirmation of EGFR mutation status.

The collection time of tumor tissues for EGFR mutation testing during the screening period may not be subject to the 28-day window, but the reported results should be no earlier than 3 months before the start of screening.

10.1.4 Randomization number assignment

Non-randomized design, with no need to assign a randomization number.

10.1.5 Data collection

The following data will be collected in the screening period according to the study plan (Appendix B):

Demographic data and other characteristics, including date of birth or age, sex, ethnicity, and smoking status.

Complete medical history: including past and current history of kidney, liver, blood system, gastrointestinal tract, endocrine, immune, metabolic, cardiovascular, respiratory, neurological, psychiatric, infectious, and inflammatory diseases. Treatment history: drug therapies (chemotherapy, prescription drugs, over-the-counter drugs, and other drugs) and non-drug therapies (e.g., surgery, radiotherapy).

Allergy history: Subjects' history of allergy to drugs or other substances.

10.1.6 Physical examination

Physical examinations will be performed once during the screening period, on C1D1, every 8 weeks in C2 and thereafter, and at the EOT visit according to the study plan (Appendix B).

Physical examination at baseline (defined as assessment performed before the start of study treatment) will cover the following systems: (1) eyes; (2) ears, nose, and throat; (3) heart and vascular system; (4) chest and respiratory system; (5) abdomen; (6) mucocutaneous system; (7) spine and extremities; (8) musculoskeletal system; (9) neurological system; (10) lymph nodes; (11) others. For subsequent routine physical examinations in each treatment cycle, significant changes from baseline should be evaluated for clinical relevance except for the neurological examination.

10.1.7 ECOG PS score

ECOG PS scoring will be performed once during the screening period, on C1D1, every 8 weeks in C2 and thereafter, and at the EOT visit according to the study plan (Appendix B).

0 = Fully active, able to carry on all predisease performance without restriction.

1 = Restricted in physically strenuous activity, but ambulatory and able to carry out work of a light or sedentary nature (e.g. light house work, office work).

2 = Ambulatory and capable of all self-care but unable to carry out any work activities. Up and about more than 50% of waking hours.

3 = Capable of only limited self-care, confined to bed or chair more than 50% of waking hours.

4 = Completely disabled. Cannot carry on any self-care. Totally confined to bed or chair.

5 = Dead.

10.1.8 Body weight and height measurement

Subjects will undergo body height and weight measurement according to the study plan (Appendix B).

10.1.9 Vital signs measurement

Vital signs measurement will be performed once during the screening period, on C1D1, every 8 weeks in C2 and thereafter, and at the EOT visit according to the study plan (Appendix B).

Vital sign measurements, including body temperature (oral or ear or axillary temperature, in °C, forehead temperature will not be acceptable), blood pressure (systolic and diastolic blood pressures), pulse, and respiratory rate, will be collected. Blood pressure and pulse are recommended to be measured after subjects sit for at least 10 min. Additional tests may be performed as deemed necessary by the investigator based on actual conditions.

10.1.10 Laboratory tests

Laboratory tests will be performed once during the screening period, on C1D1, every 8 weeks in C2 and thereafter, and at the EOT visit according to the study plan (Appendix B).

Additional tests may be performed as deemed necessary by the investigator based on actual conditions. The values, units, and reference ranges for test results should be documented on the eCRFs.

The laboratory test items are shown in Table 4.

Table 4. Laboratory test items

Clinical biochemistry	Hematology	Urinalysis (qualitative)
Serum (S)/Plasma (P) — albumin	Blood (B) — hemoglobin	U — urine specific gravity
S/P-ALT	B — leukocytes	U — pH
S/P-AST	B — hematocrit	
S/P — alkaline phosphatase	B — red blood cell count (RBC)	
S/P — bilirubin, total bilirubin		U — urine protein
S/P — Ca, total blood calcium	B — neutrophil count and/or proportion	U — occult blood
S/P — creatinine	B — lymphocyte count and/or percentage	U — nitrite
S/P — creatine phosphokinase	B — monocyte count and/or proportion	
	B — basophil count or percentage	
S/P-LDH	B — eosinophil count or percentage	
	B — platelet count	
S/P — magnesium		
S/P — potassium		
S/P — sodium		
S/P — either urea nitrogen or urea		

If the patient shows AST or ALT ≥ 3 × ULN and total bilirubin ≥ 2 × ULN, refer to Appendix C (Hy's Law) for actions required in case of a combined increase in aminotransferases and total bilirubin for further guidance.

10.1.11 ECG

12-lead ECG will be performed once during the screening period, on C1D1, every 8 weeks in C2 and thereafter, and at the EOT visit according to the study plan (Appendix B).

Subjects are recommended to rest in a semi-supine position for at least 10 min before the given time point for 12-lead ECG. Subjects will undergo 3 separate ECG measurements at an interval of ≤ 10 min during each ECG measurement.

In this study, abnormal QT interval changes will be judged based on the mean of 3 QTcF intervals.

10.1.12 Echocardiography

Echocardiography will be performed as clinically indicated throughout the treatment period according to the study plan to assess the left ventricular ejection fraction (LVEF).

When possible, one subject should undergo echocardiography performed by the same operator using the same machine throughout the study.

10.1.13 Ophthalmological examination

During the screening period and throughout the study, ophthalmological examinations will be performed according to the study plan (Appendix B) and when clinically indicated, and it will be determined by the investigator as to which ophthalmological examination items are to be performed. Ophthalmological examination results should be documented on the eCRFs. Subjects who develop corneal ulcer will no longer be permitted to restart receiving the investigational medicinal product.

10.2 Efficacy Evaluation

10.2.1 Response evaluation in solid tumors as per RECIST 1.1

During the oncological assessments by investigator, RECIST 1.1 will be used to assess the response to the anti-tumor treatment in each subject and calculate PFS, ORR, DoR, DCR, and DepOR. Appendix D provides the criteria in RECIST 1.1 for measurable lesions, non-measurable lesions, TLs, NTLs, complete response (CR), partial

response (PR), stable disease (SD), and progressive disease (PD). The analysis of the primary endpoint of this study will be based on the tumor response assessment made by the investigator as per RECIST 1.1, and the investigator will also manage the subjects based solely on the assessment results, unless medically contraindicated or faced with force majeure factors (such as update of the examination instrument by the study site).

Baseline CT or MRI assessments of the chest, abdomen, and brain tissues must be performed within 28 days before the start of study treatment (not subject to the time of signing the ICF), and should be performed as close as possible to the start of study treatment. The investigator should perform additional imaging examinations based on subjects' signs and/or symptoms. The subsequent follow-up assessments should be performed using the method (CT/MRI) that is the same as that for baseline assessment.

All enrolled subjects must have brain metastases as target lesions, and baseline imaging assessment of the brain must be performed, with MRI (enhanced) as the preferred examination method.

Bone scans should be performed if there are indications for bone metastases (results within 3 months are acceptable), and positive sites found by bone scans should be confirmed by CT/MRI/X-ray. Only confirmed lesions can be recorded as non-target lesions. Subsequently, the bone metastases should be followed up by the same examination method as at baseline. During the study, bone scans may be added as needed.

During the treatment period, tumor response assessment will be performed once every 8 weeks (± 1 week) until progressive disease assessed by investigator per RECIST 1.1 (see Section 12.1.3 or 9.1.3.4). Tumor assessments should be performed according to the study plan whenever possible. If subjects are assessed at a time point beyond the scheduled time window (± 1 week) and no progressive disease occurs, they should undergo subsequent assessments at the pre-specified time points whenever possible while continuing study treatment. If tumors are suspected at any other sites, the corresponding imaging examinations should be performed at the relevant sites.

To achieve progression on the basis of the non-target lesion(s), there must be an overall level of substantial worsening in non-target lesion(s) such that, even in the presence of SD or PR in the target lesion(s), the overall tumor burden has increased sufficiently to merit discontinuation of treatment. A modest increase in the size of one or more non-target lesions is generally not sufficient to qualify for progression; therefore, it is uncommon to define overall tumor progression only with changes in non-target lesions in the face of SD or PR of target lesions.

At each visit, objective tumor response will be assessed as per RECIST 1.1, and classified into CR, PR, SD, and PD.

For the assessment of target lesion progression, the smallest lesion size in the entire course of treatment should be taken as reference, and an increase meeting the criteria will qualify for PD. If no progressive disease occurs, tumor response (CR and PR) and stable disease (SD) should be evaluated taking baseline lesion(s) as reference.

10.2.2 EGFR mutation testing at screening

The tumor tissue samples used for testing can be derived from primary tumors or metastatic tumors.

The tumor biopsy samples used for screening cannot be derived from tumor lesions that have received local treatment such as irradiation. The biopsy samples may not be subject to the 28-day screening window (test reports issued within 3 months before screening are deemed valid). If the biopsy sample is from Stage IV tumor tissue, a repeated biopsy is not required.

Patients who fail screening due to the first tumor tissue testing may be re-screened if the investigator believes that the failure may be related to sample collection and another biopsy sampling is required. Patients must sign the informed consent form again before re-screening. Upon re-screening, the laboratory tests and imaging examinations are not required to be repeated if the previous tests/examinations are within the time window, and re-testing/re-examination is required if the previous ones are beyond the time window.

In case the tumor tissue is not accessible by biopsy and the sample submitted is a blood sample, if the test result is negative for EGFR mutation, it is recommended to use a tumor tissue sample for a second testing. If a tumor tissue sample is not available, the subject will not be enrolled.

10.3 Safety Evaluation

Occurrence of AEs; occurrence of SAEs; proportion of patients withdrawn due to AEs; changes in laboratory tests (blood biochemistry, hematology, and urinalysis); changes in vital signs, physical examination, body weight, ECG, LVEF, ECOG score, and ophthalmological examination.

10.4 Sampling and Testing Design

10.4.1 Exploratory sampling

Venous blood samples: Samples need to be collected pre-administration on C1D1, within ± 7 days of administration on C2D1, and within 1 week after PD. Blood collection must be completed on the same day. See Table 5 for specific blood sampling time points.

Table 5. Sampling plan

Time	Cycle 1	Cycle 2	PD
	D1	D1	D1-7
Within 7 days before administration	×	×	×

10.4.3 Precautions for sampling

During the PK study, patients are prohibited from using any drugs or foods with a narrow therapeutic window that are strong inhibitors or inducers of CYP3A4 or sensitive substrates of CYP3A4 (e.g., pitaya, grapefruit, mango, and juice containing the above fruits). In addition, during this period, you should eat a light diet, avoid smoking and drinking, avoid tea, caffeinated and carbonated beverages, and avoid strenuous exercise.

10.5 Management of Biological Samples

10.5.1 Sample collection volume

According to the study plan, for subjects undergoing the blood collection volume is 45 mL (Table 6) (C1D1, C2D1, within one week after PD).

Table 6. Volume of blood collected

Visit	Safety (mL)[a]	Gene mapping (mL)[b]
C1D1	15	8
C2D1	15	
D1–7 after PD	15	8

Note: a, 6 mL for hematology and 9 mL for blood biochemistry; b, 8 mL for gene mapping sampling.

The total sample size required may be adjusted according to the protocol-specific procedures, changes, as well as methods established by the sample testing unit.

Laboratory safety assessments will be assessed using the respective established method in the laboratory of each study site. Therefore, the sample size required will vary from site to site.

10.5.2 Destruction of biological samples

Biological samples will be retained for at most 3 years from the date of the last patient last visit (LPLV) and will be destroyed thereafter.

10.5.3 Labeling and supervision of biological samples

Samples other than clinical test samples will be identified using the study number and subject screening number to ensure that the data of the samples can be linked to the clinical data. Samples may be destroyed in the event of patient's withdrawal of consent and reviewed by the regulatory authorities.

11.0 ADVERSE EVENTS AND MEDICAL MANAGEMENT

11.1 Adverse Events

11.1.1 Definition of adverse event

An adverse event (AE) is an unexpected medical condition or worsening of a pre-existing medical condition after or during exposure to the investigational medicinal product, regardless of whether or not it has a causal relationship with the investigational medicinal product. An unexpected medical condition can be a symptom (e.g., nausea or chest pain), a sign (e.g., tachycardia or hepatomegaly), or an abnormality on examination (e.g., laboratory test or ECG).

AEs include but are not limited to the following conditions: 1) Worsening of pre-existing (before entering the clinical trial) medical conditions or diseases (including worsening of symptoms, signs, and laboratory abnormalities). 2) Any new AEs: any new untoward medical conditions (including symptoms, signs, and newly diagnosed diseases). 3) Clinically significant abnormal laboratory findings.

Exacerbation of the disease under study and its associated symptoms or signs are not considered AEs.

Any untoward clinical occurrence in a subject from signing the ICF to the first dose will be recorded as a medical history/concomitant disease, rather than an AE, unless any of the following conditions is met: injury/damage caused by any procedure related to the clinical laboratory tests; untoward occurrence caused by drug discontinuation related to the study protocol; untoward occurrence caused by drugs other than the investigational medicinal product which are taken as part of the treatment regimen. When a past medical history worsens during the study and is judged as an AE by the investigator, "Name of AE" should be filled in with "Aggravation of XX (name of past medical history)". The start date of the AE is the date of past medical history aggravation, and the end date of the AE is the date of recovery to past medical history.

11.1.2 Definition of serious adverse event

A serious adverse event (SAE) is any AE that occurs during the study period and meets one or more of the following criteria:

- Results in death
- Is life-threatening
- Requires or prolongs hospitalization
- Causes persistent or significant disability/incapacity or substantial disruption of the ability to conduct normal life functions
- Result in congenital anomalies or birth defects
- Other important medical events that require medical intervention to prevent one of the above outcomes

* "Hospitalization", as is required or prolonged due to diagnosis of an existing condition or elective surgery, due to efficacy evaluation for the study, or due to a specified course of treatment for the study target disease, will not be recorded and reported as an SAE.

Examples include but are not limited to the following, at the investigator's discretion:

- Rehabilitation facility
- Nursing home
- Routine emergency room admission
- Day surgery (e.g., outpatient/day/ambulatory surgery)
- Hospitalization for a pre-existing condition with no new AE or exacerbation of the pre-existing condition
- Hospitalization for administrative reasons (e.g., routine annual physical examination)
- Protocol-specified hospitalization during the clinical trial
- Elective hospitalization not related to worsening of AEs (e.g., elective cosmetic surgery)
- Hospital admission for blood product use only
- Social reasons (health care reimbursement)
- Hospitalizations and/or surgical procedures planned before or during the study for diseases existing before enrollment in the study are not considered SAEs as long as there is no unexpected worsening of the diseases (i.e., natural disease course) during the study

11.1.3 Documentation of adverse events

11.1.3.1 Time period for collecting AEs and SAEs

In this study, AEs will be collected from the signing of the informed consent form until 28 days after treatment discontinuation. Any new or unresolved AEs observed during the 28-day follow-up should be followed up until recovery, stabilization, or until the subject is lost to follow-up. After the end of the safety follow-up, only treatment- or procedure-related SAEs will be collected.

11.1.3.2 Follow-up of unresolved AEs

Any unresolved AE that is medically indicated at the patient's last study visit will be followed up by the investigator, but will not be further documented. The investigator reserves the right to collect information about ongoing AEs after the end of the study, if necessary. If the investigator learns of any new SAE at any time after a patient has completed the study and considers the event possibly related to aumolertinib, the marketing authorization holder (Jiangsu Hansoh Pharmaceutical Group Co., Ltd.) should be notified within 24 h of learning of the SAE.

11.1.3.3 Key information on adverse events to be collected

Important information collected for each AE will include, but is not limited to, the following:

- Diagnosis/Description of the AE
- Start and end dates of the AE
- CTCAE grade
- Whether the AE is a serious adverse event
- Measures taken with the investigational medicinal product due to the AE
- Causal relationship determined by the investigator
- Treatment of the event (drug and/or non-drug therapies)
- Outcome

For SAEs, the following information will be collected:

- Event name (diagnosis)
- Description of the event
- Severity of the event
- Outcome of the event
- Start and end dates of the event
- Any relevant medical history, concomitant medications/treatments, and laboratory test results

- Treatment of the event (drug and non-drug therapies)
- Causal relationship determined by the investigator
- Cause of death (for a fatal event)

All AEs will be graded according to the grading criteria in the Common Terminology Criteria for Adverse Events (CTCAE) Version 5. For AEs not graded as per CTCAE, the recommendations in the CTCAE guidelines should be followed to convert mild, moderate, and severe events into CTCAE grades.

11.1.4 Causal relationship determination

The investigator will assess the causality between the investigational medicinal product and each AE. The criteria for causality between AEs and the investigational medicinal product are shown in Table 7.

Table 7. Criteria for causal relationship between adverse events and the investigational medicinal product

Causality	Criteria
Not related	The AE is not related to the use of the investigational medicinal product. For example, no investigational medicinal product is used.
Unlikely related	There is no evidence to suggest a causal relationship between the occurrence of the event and the investigational medicinal product. The occurrence of the AE is more likely related to other factors, such as concomitant medications or diseases. However, a correlation between the two cannot be ruled out.
Possibly related	The AE follows a reasonable temporal sequence from the administration of the investigational medicinal product, and may be caused by the investigational medicinal product, but can readily have been produced by other factors such as concomitant medications or diseases. No drug withdrawal is performed or the information is unclear.
Definitely related	The AE has been identified to follow a known response pattern to the drug and cannot be explained by other factors (such as concomitant medications and diseases). The time of onset strongly suggests a causality (e.g., dechallenge and rechallenge results).
Indeterminable	There is insufficient information to make a judgment on the causality between the event and the investigational medicinal product. The investigator may change her/his causality assessment and modify the corresponding AE/SAE report based on subsequent follow-up information.

11.1.5 Adverse events based on symptoms and signs

All AEs spontaneously reported by the patient or health care provider (open-ended question from the study personnel "Have you had any health problem since your last visit/last inquiry") or identified by observation will be collected and recorded.

11.1.6 Adverse events based on examinations and tests

The results of protocol-specified laboratory tests and examinations, namely vital signs, ECG, and other safety assessments, will be summarized in the clinical study report. If these parameters suggest abnormalities compared with baseline, they will be reported as AEs only if they meet the criteria for SAEs, are judged by the investigator as clinically significant, or lead to discontinuation of study treatment (other than due to progressive disease).

When an abnormality in a laboratory value, vital sign, ECG, or other safety assessment is reported as an AE, the investigator should use the clinical term rather than the laboratory term whenever possible (e.g., anemia rather than decreased hemoglobin value).

For AEs of abnormalities in hematology, they should be reported specifically as "white blood cell count decreased", "neutrophil count decreased", "anemia", "platelet count decreased", etc., rather than using the AE name "myelosuppression". Reporting of percentage abnormality AEs (such as "neutrophil percentage decreased") is not required.

In case of abnormal liver enzymes, it should be reported by the name "XX enzyme increased/decreased", and ambiguous names such as "transaminases abnormal/increased/decreased" and "liver enzyme abnormal/increased/decreased" should not be used in reporting.

When changes occur in bilirubin-related indicators, "total bilirubin increased/decreased" and/or "direct bilirubin increased/decreased" and/or "indirect bilirubin increased/decreased" should be used in reporting. Ambiguous names such as "bilirubin abnormal/increased/decreased" and "blood bilirubin abnormal/increased/decreased" are not allowed for reporting.

Laboratory abnormalities due to unequivocal progressive disease should not be reported as AEs. Any new or

worsening clinically relevant abnormality in physical examination as compared to the baseline will be reported as an AE.

11.1.7 Hy's law

If subjects show AST or ALT ≥ 3 × ULN and total bilirubin ≥ 2 × ULN, it may need to be reported as an SAE. Cases meeting Hy's law are subject to expedited reporting as per regulatory guidelines. The investigator is responsible for determining whether a subject meets Hy's law immediately. A detailed description of Hy's law and its handling measures are provided in Appendix C.

11.1.8 Progressive disease

Progressive disease and its associated signs and symptoms are considered an exacerbation of the patient's condition, which may be an increase in the severity of the disease under study and/or an increase in the symptoms of the disease.

New lesions of the primary tumor under study or progression of existing metastases are considered progressive disease rather than an AE. Unequivocal progressive disease during the study will not be reported as an AE.

11.1.9 New cancers

A new cancer should be considered an SAE and it usually meets the criteria of "severe". New cancers are those that are not the primary reason for receiving the study treatment and are discovered after a patient is enrolled in this study. New cancers do not include metastases of the primary cancer.

11.1.10 Handling of deaths

All deaths that occur during the study or during the follow-up period after treatment discontinuation should be reported as follows:

- Deaths that are clearly related to progressive disease should be reported to the CRA at monitoring visits and recorded, but should not be reported as SAEs during the study.
- If the death is not clearly caused by the progression of the study disease, the AE resulting in the death should be reported to the CRA as an SAE within 24 h of awareness. The report should contain the evaluation of progressive disease, primary cause of death, and other possible causes of death.
- Deaths of unknown causes should be reported as SAEs within 24 h of the study site's awareness, but every effort should be made to identify the cause of death. An autopsy may be helpful in assessing the cause of death. If an autopsy is performed, a copy of the autopsy results should be reported to the drug marketing authorization holder (Jiangsu Hansoh Pharmaceutical Group Co., Ltd.) within 24 h after the results are available.

11.1.11 Reporting of adverse events

All SAEs, regardless of whether considered causally related to the IMP or study procedures, should be reported. All SAEs will be recorded. The investigator or other site personnel will immediately notify the drug marketing authorization holder (Jiangsu Hansoh Pharmaceutical Group Co., Ltd.) of any SAE that occurs throughout the study (from the time of informed consent to the end of the follow-up period), and no later than 24 h after awareness of the SAE.

For fatal or life-threatening AEs with significant or relevant information missed, subsequent active actions will be taken immediately. The investigator or other personnel at the study site should immediately notify the sponsor and funder of any subsequent information about the SAE in the previous report, no later than 24 h after awareness of the subsequent information about the SAE. The contact information is shown in Table 8.

Table 8. Contact information for AE reporting

Name	Responsibility in study	Address and phone number
Yun Fan	Sponsor's study team member AE/SAE<br><br>24-hour emergency contact	No. 38 Guangji Road, Banshan Bridge, Gongshu District, Hangzhou, Zhejiang, China<br>Mobile: +86-13858182310<br>E-mail: fanyun@zjcc.org.cn
Kun Liu	Project management team<br><br>24-hour emergency contact	Building 16, No. 989 Dongfang Road, Pudong New Area, Shanghai, China<br>Mobile: +86-18652100216<br>Email: liuk6@hspharm.com

11.2 Overdose

Any subject receiving a higher-than-expected dose of the drug should be closely monitored by the investigator and should receive appropriate supportive care and close follow-up. Overdoses should be documented as follows:

- AEs or symptoms associated with an overdose should be recorded in both the AE section and the overdose section of the medical record.
- An overdose without associated symptoms should only be recorded in the overdose section of the medical

record, i.e., an overdose without discomfort will not be considered an AE and does not need to be recorded in the AE section of the medical record.

If an overdose occurs during the study, the investigator or other personnel at the study site should inform the sponsor and the funder immediately or within 24 h of knowing of the event. Designated representative(s) of the funder will work with the investigators to ensure that all necessary information is recorded in the sponsor's and funder's secure data entry systems.

SAEs related to overdose should be reported according to the SAE reporting procedure. Other conditions associated with overdose must be reported within 30 days of occurrence.

11.3 Pregnancy Report

The collection period of pregnancy reports lasts from the first dose to the end of the safety follow-up period.

All pregnancy reports and related outcomes during the clinical trial should be reported to the sponsor and the funder.

The investigator should complete the "Pregnancy Report Form" and submit it to the sponsor and the funder within 24 h of awareness of any pregnancy event.

11.3.1 Pregnancy of female subjects

If a female subject becomes pregnant, the subject should immediately discontinue treatment, and the investigator should report to the sponsor and the funder within 24 h of awareness of the pregnancy. The investigator should follow up the pregnancy outcome until 28 days after delivery and report the outcome to the sponsor and the funder. Pregnancy itself is not considered an AE; only the "Pregnancy Report Form" needs to be completed and submitted to the sponsor and the funder within 24 h. Unless there is any negative pregnancy outcome, such as stillbirth, spontaneous abortion, and fetal malformation, it will be considered an SAE and needs to be reported according to the time requirements for SAEs. An uncomplicated selective abortion will not be considered an AE.

11.3.2 Pregnancy of partners of male subjects

If the partner of a male subject becomes pregnant during the clinical trial, the subject may continue the clinical trial. The investigator should report to the sponsor and the funder within 24 h of awareness of the partner's pregnancy. Pregnancy of the partner of a male subject will not be considered an AE. The investigator should follow up the pregnancy outcome of the partner until 28 days after delivery and report the outcome to the sponsor and the funder. Any negative pregnancy outcome, such as stillbirth, spontaneous abortion, and fetal malformation, is considered an SAE and needs to be reported according to the time requirements for SAEs.

Information regarding pregnancy should not be collected from male subjects, but should be collected from their partners themselves with the informed consent of the partners.

11.4 Recommended Toxicity Management

11.4.1 Aumolertinib dose reduction

During toxicity management, aumolertinib dose can be reduced in accordance with the dosages specified in Table 9 below.

Table 9. Guidelines for dose reduction levels of the investigational medicinal product

**Aumolertinib**			
	**Initial therapy**	**Dose reduction therapy 1**	**Dose reduction therapy 2**
Dose	Aumolertinib, 165 mg/day	Aumolertinib, 110 mg/day	Aumolertinib, 55 mg/day
Drug	Aumolertinib active drug, 55 mg/tablet, 3 tablet	Aumolertinib active drug, 55 mg/tablet, 2 tablet	Aumolertinib active drug, 55 mg/tablet, 1 tablet
Dosage and route of administration	p.o., QD	p.o., QD	p.o., QD

11.4.2 Dose modifications associated with adverse events

If a subject experiences any toxicity of CTCAE Grade ≥ 3 and/or any toxicity (any grade) deemed unacceptable by the investigator, which is judged by the investigator to be treatment-related, the study treatment should be interrupted, and supportive care should be provided as needed in accordance with local practices/guidelines. If the toxicity is relieved or returns to ≤ CTCAE Grade 1–2 within 3 weeks after onset, the IMP at the original dose or dose reduction therapy 1 in Table 7 may be given again. If the original dose is resumed, the subject should be closely monitored for 3 days; if the same toxic effect occurs within 3 days, dose reduction therapy 1 should be considered. Decisions should be made in agreement between the investigator and the sponsor.

If the toxicity does not recover to ≤ CTCAE Grade 1–2 within 3 weeks after onset, dose reduction therapy 2 should be considered. If the toxicity still does not recover to ≤ CTCAE Grade 1–2 within 3 weeks or the same toxic effect

occurs within 3 days, the subject should be withdrawn from the study and should be followed up until the toxicity is recovered.

"Treatment-related" herein refers to definitely related, possibly related, and indeterminable, not including unlikely related and not related. Specific dose modification principles are presented in Table 10.

Table 10. Dose modification principles for aumolertinib in case of treatment-related AEs

	**Treatment-related AEs**	**Dose modification**
Heart	Corrected QT (QTcF) interval > 500 ms observed in at least 2 separate ECG assessments	Interrupt the study treatment for a maximum of 3 weeks[*].<br>• If the baseline QTcF interval is > 480 ms and the abnormal value improves to the baseline level within 3 weeks, resume the study treatment at 55 mg;<br>• If the baseline QTcF interval is ≤ 480 ms and the abnormal value improves to the baseline level within 3 weeks:<br>- Resume the study treatment at the original dose for the first occurrence;<br>- Resume the study treatment at one dose level lower if it reoccurs.
	QTcF interval prolonged in combination with any of the following: symptoms or signs of torsade de pointes, polymorphic ventricular tachycardia, or serious arrhythmia	Permanently discontinue the study treatment.
	Absolute asymptomatic left ventricular ejection fraction (LVEF) < 50% with a decrease of > 10% relative to baseline	Interrupt the study treatment for a maximum of 3 weeks[*]. If the LVEF improves to the baseline level within 3 weeks:<br>• Resume the study treatment at the original dose for the first occurrence;<br>• Resume the study treatment at one dose level lower if it reoccurs.
	Symptomatic congestive cardiac failure	Permanently discontinue the study treatment.
Blood creatine phosphokinase (CK)	ULN < CK value ≤ 5 × ULN (Grade ≤ 2)	If no significant muscular symptoms[#] are observed (Grade ≤ 2), the dose may not be modified.
	5 × ULN < CK value ≤ 10 × ULN (Grade 3), regardless of muscular symptoms[#]	Interrupt the study treatment for a maximum of 3 weeks[*]. If the value improves to Grade ≤ 2 (5 × ULN) within 3 weeks:<br>• Resume the study treatment at the original dose for the first occurrence;<br>• Resume the study treatment at one dose level lower if it reoccurs.
	CK value > 10 × ULN (Grade 4), regardless of muscular symptoms[#]	Interrupt the study treatment for a maximum of 3 weeks[*]. If the value improves to Grade ≤ 2 (5 × ULN) within 3 weeks:<br>• Resume the study treatment at one dose level lower for the first occurrence;<br>• Permanently discontinue the study treatment for the second occurrence.
	Muscular symptoms[#] (Grade ≥ 3), with or without CK increased	Interrupt the study treatment for a maximum of 3 weeks[*]. If the muscular symptoms improve to Grade ≤ 2 within 3 weeks:<br>• Resume the study treatment at the original dose for the first occurrence;<br>• Resume the study treatment at one dose level lower if it reoccurs.
Others	Grade ≥ 3 adverse reactions	Interrupt the study treatment for a maximum of 3 weeks[*]. If the adverse reaction improves to Grade ≤ 2 within 3 weeks:<br>• Resume the study treatment at the original dose for the

		first occurrence;<br>• Resume the study treatment at one dose level lower if it reoccurs.
At any time, if 55 mg is still intolerable (occurrence of Grade ≥ 3 adverse reactions), permanent discontinuation of study treatment is recommended.		

Note: The severity mentioned above is graded according to the National Cancer Institute - Common Terminology Criteria for Adverse Events Version 5.0 (NCI-CTCAE 5.0). * If the study treatment is interrupted for more than 3 weeks and the resumption criteria are not met, the study treatment should be permanently discontinued. # Muscular symptoms (including but not limited to): muscle pain, muscle tenderness, muscle twitching, or muscle weakness.

When several AEs occur simultaneously, the dose should be modified according to the most severe AE; once the dose is reduced, it cannot be returned to the previous level. Subjects who discontinue treatment should be observed until the resolution of toxicity; dose interruption does not affect scheduled tumor evaluation. The above decisions should be made in agreement between the investigator and the funder.

For certain patients, if the investigator decides to adopt measures such as continuing treatment without interruption, interrupting treatment, modifying the dose, or permanently discontinuing the study treatment, not in accordance with the dose modification provisions of this section, but based on the benefit/risk considerations, agreement should be reached with the funder.

11.4.3 Skin reaction

In order to reduce the probability or severity of skin reactions in subjects, it is recommended that subjects be protected against light from the start of study treatment to 3–4 weeks after the last dose.

If a subject experiences any skin reaction, the following formulations may be used based on the investigator's evaluation: steroid cream, topical or systemic anti-inflammatory drugs, topical antihistamines, or retinoid cream. If necessary, symptomatic treatments can be provided.

Skin reactions should be recorded in the AE section of the medical record, and the following information should be included:

- Changing characteristics of the skin reaction.
- CTCAE grade of the skin reaction.
- When necessary, images should be taken to document the skin reactions, and these images should be reviewed by external experts.
- When necessary, skin biopsy may be performed.

11.4.4 Gastrointestinal toxicity

When a subject experiences nausea and/or vomiting, antiemetic therapy can be used for control. In case of a CTCAE Grade ≥ 3, clinically significant, or intolerable diarrhea that the investigator believes is related to the study medication, necessary management or dose modification can be made. This should be recorded in the AE section of the medical record.

11.4.5 Interstitial lung disease

If new or worsening pulmonary symptoms (e.g., dyspnea) are observed or an imaging abnormality suggests the presence of interstitial lung disease, study treatment should be interrupted and the funder should be notified.

All diagnosis results (including HRCT, blood and sputum cultures, and hematology parameters) of the patients will be investigated by the study personnel. The investigator is strongly advised to perform a thorough diagnostic workup on patients to rule out other causes such as lymphangitic carcinomatosis, infection, allergy, edema due to cardiac disease, or pulmonary hemorrhage.

The diagnosis of interstitial lung disease should be considered if confirmed by HRCT scans and other causes of respiratory symptoms have been ruled out, and study treatment should be permanently discontinued. In the absence of a diagnosis of interstitial lung disease, treatment may be resumed after comprehensive safety assessment by the investigator.

A subject who is determined to have developed interstitial lung disease should permanently discontinue the study treatment.

12.0 ENDPOINT EVALUATION AND STATISTICAL ANALYSIS

12.1 Anti-Tumor Activity Evaluation

12.1.1 Investigator assessment as per RECIST 1.1

At each visit, the investigator will assess the patient's tumor response to treatment as CR, PR, SD, or PD as per RECIST 1.1 by comparing with baseline and previous assessments, depending on patient's status. The progression of TLs will be calculated by comparing with tumor burden at its nadir (i.e., the smallest sum of previously recorded diameters). In the absence of progression, tumor response (CR and PR) and stable disease (SD) will be calculated by

comparing with baseline tumor measurements obtained prior to the start of the treatment. For patients with indeterminable tumor status, if there is evidence of progression, they will be considered to have PD; if there is no evidence of progression, they will be considered inevaluable (NE). The endpoints of this study will be analyzed based on the tumor response assessment by the investigator per RECIST 1.1, and the investigator will also manage subjects only based on the assessment results.

The investigator will evaluate patient's response to treatment using RECIST 1.1 (see Appendix D) by determining the objective response rate (ORR), disease control rate (DCR), duration of response (DOR), and progression-free survival (PFS). In addition to the overall tumor imaging evaluation performed by the investigator as per RECIST 1.1, for systemic and/or intracranial lesions (no more than 5 target lesions in the whole body) selected for overall evaluation, the respective intracranial and systemic response evaluation (intracranial: iPFS, iORR, iDCR, and iDoR; systemic: PFS, ORR, DCR, and DoR) will be performed according to the response evaluation criteria in RECIST 1.1, and the investigator does not need to select additional lesions.

12.1.2 Progression-free survival (PFS)

According to RECIST 1.1, PFS is defined as the time from the treatment initiation to the first occurrence of investigator-assessed objective tumor progression or death. In the absence of PD, even if the patient has discontinued aumolertinib treatment or received another anti-cancer treatment before PD, his/her imaging tumor evaluation should still be collected until RECIST-defined PD. Symptomatic deterioration is not considered a progression event.

Subjects who have not progressed or died at the time of analysis will be censored at the last date of their last RECIST assessment. If a subject has PD or dies after 2 or more missing visits, he/she will be censored at the last date of his/her last RECIST assessment. PFS-derived data are based on assessment date rather than visit date.

Systemic PFS and iPFS will be assessed separately:

Systemic progression-free survival (PFS):

Systemic PFS is defined as the time from the treatment initiation to the first documentation of systemic PD or death due to any cause, with other principles as above.

Intracranial progression-free survival (iPFS):

iPFS is defined as the time from the treatment initiation to the first documentation of intracranial PD or death due to any cause, with the same principles as above.

12.1.3 Objective response rate (ORR)

As per RECIST 1.1, systemic ORR is defined as the percentage of patients with at least 1 CR or PR before PD. Data until PD or last evaluable data in the absence of PD will be recorded in the assessment of ORR. However, any CR or PR that occurs after discontinuation of the study treatment and further anti-tumor treatment will not be included in the calculation of ORR.

Objective response rate will be assessed separately for intracranial lesions (iORR) and systemic lesions (ORR).

12.1.4 Duration of response (DoR)

DoR is defined as the time from the date of first compliance with response to the date of PD or death due to any cause. The end date of the response should be consistent with the date of the endpoint PFS. The start of DoR is defined as the latest date of the visit at which PR or CR criteria are first met. If a patient does not progress after a response, the censored time for PFS will be used for DoR.

Intracranial duration of response (iDoR) and systemic duration of response (DoR) will be assessed separately.

12.1.5 Disease control rate (DCR)

DCR is defined as the proportion of patients with the best overall response, which includes CR, PR, or SD.

Intracranial disease control rate (iDCR) and systemic disease control rate (DCR) will be assessed separately.

12.1.6 Overall survival (OS)

OS is defined as the time from the date of the first dose to the date of the patient's death due to any cause. A patient who has not died at the time of statistical analysis will be censored at the time point when the patient is last known to be alive.

12.2 Safety Evaluation

All safety data will be summarized at the end of the study. All data from the initiation of treatment until 28 days after the last dose will be summarized together in the safety evaluation. Data prior to initiation of treatment will be summarized but not included in the summary table of AEs.

12.4 Statistical Analysis Methods

12.4.1 Definitions of statistical analysis sets

- **Full analysis set (FAS)**

The FAS is defined as all patients with at least one dose of the study drug.

- **CNS evaluable-for-response set (cEFR)**

The cEFR is defined as a subset of the FAS and includes patients who have at least one measurable brain lesion. Baseline and demographic characteristics will be analyzed in the FAS and cEFR, and the efficacy evaluation will be performed in both the FAS and the cEFR. The results will be mainly based on the FAS.

- **Safety set (SS)**

The SS is defined as all patients who have received at least one dose of the study treatment. Safety evaluation will be based on the SS.

12.4.2 Efficacy analysis

Efficacy endpoints will be analyzed using results evaluated by the investigator as per RECIST 1.1.

12.4.2.1 Analysis of primary efficacy endpoint (12-month PFS rate)

The Kaplan-Meier plot will be generated, and the 12-month PFS rate, median PFS and its 95% CI will be calculated. In addition, the PFS rate at 6, 12, 18, and 24 months will be summarized.

12.3.2.2 Analysis of secondary efficacy endpoints

- **iPFS**

The Kaplan-Meier plot will be generated, and the median iPFS and its 95% CI will be calculated. In addition, the iPFS rate at 6, 12, 18, and 24 months will be summarized.

- **OS**

The Kaplan-Meier plot will be generated, and the median OS and its 95% CI will be calculated. In addition, the OS rate at 6, 12, 18, and 24 months will be summarized.

- **ORR (including systemic and intracranial ORR)**

ORR will be summarized as percentage, and its 95% CI will be calculated.

- **DoR (including systemic and intracranial DoR)**

The Kaplan-Meier plots will be generated, and the median DoR and 95% CI will be calculated.

- **Analysis of DCR (including systemic and intracranial DCR)**

DCR will be summarized as percentage, and its 95% CI will be calculated.

12.4.3 Safety analysis

12.4.3.1 Adverse events

The AEs that occur from the initiation of treatment until 28 days after the last dose will be summarized in the safety evaluation. The AEs that occur prior to initiation of treatment and AEs that occur after 28 days following the last dose will be tabulated separately and not included in the summary table of AEs.

Treatment-emergent AEs will be summarized by treatment group, mainly including:

1) Number and proportion of patients with at least one AE;
2) Number and proportion of patients with at least one treatment-related AE;
3) Number and proportion of patients with at least one severe (CTCAE Grade $\geq$ 3) AE;
4) Number and proportion of patients with at least one severe (CTCAE Grade $\geq$ 3) treatment-related AE;
5) Number and proportion of patients with at least one SAE;
6) Number and proportion of patients with AEs resulting in dose modification;
7) Number and proportion of patients with AEs resulting in withdrawal;
8) Number and proportion of patients with AEs resulting in death.

All AEs will be summarized by SOC and PT. All AEs will be tabulated.

12.3.3.2 Laboratory tests

For the laboratory test values which are measurement data (such as body weight, vital signs, hematology, urinalysis, and blood biochemistry), the baseline, post-dose, and post-dose change data will be summarized by each follow-up visit and treatment group; for qualitative data (such as normal/abnormal and clinically significant/insignificant test results), the changes from baseline to each post-dose follow-up will be summarized in a list.

12.3.3.3 ECG analysis

The mean ± standard deviation, maximum, minimum, and median will be used to describe the measured values and changes in heart rate, PR interval, QT interval, and QTcF interval before and after treatment. According to the normal and abnormal findings judged by the investigator, the normal and abnormal changes before and after treatment will be described.

Abnormal QTcF interval at baseline and after treatment will be described by the proportion of QTcF interval change from baseline of $\leq$ 30 ms, 30–60 ms, and > 60 ms after treatment (maximum), and the proportion of QTcF interval change from baseline of $\leq$ 30 ms, 30–60 ms, and > 60 ms after treatment.

12.3.3.4 Ophthalmological examination

For qualitative data for the ophthalmological examination (such as normal/abnormal and clinically significant/insignificant test results), changes from baseline to each follow-up post-dose will be summarized in a list.

12.3.3.5 ECOG PS score

Changes in the ECOG PS score from baseline to each follow-up post-dose will be summarized in a cross-tabulation.

12.4.4 Pharmacokinetic analysis

The pharmacokinetics of aumolertinib and its metabolite HAS-719 will be characterized, including the cerebrospinal fluid concentrations of aumolertinib and its metabolite HAS-719.

13.0 DATA PROCESSING AND RETENTION

Full details of data processing procedures will be documented in a separate data management plan.

13.1 Electronic Case Report Form

Each patient who signed the informed consent form will be required to complete the eCRF.

The sponsor or designated personnel will provide the study sites with access to the eCRFs. Training will be provided by the funder to the personnel using the eCRFs. Information to be collected in this study using eCRFs will be transmitted to the sponsor, the funder, and regulatory authorities. The data will be entered directly into the eCRFs. All amendments should be recorded, including the old information, the new information, the person making the amendment, the date of the amendment, and the reason for the change. In addition, the reasons for major amendments should be included.

The principal investigator must review the eCRFs for integrity and accuracy and must sign and date the eCRFs. Besides, the investigators must retain full responsibility for the accuracy and authenticity of all data entered into the eCRFs.

The eCRFs will be reviewed for integrity and accuracy during periodic visits by the authorized CRA. The sponsor or designated personnel will be permitted to review patients' medical and hospital records pertinent to the study to ensure the accuracy of the eCRFs. All eCRF data are owned by the sponsor and the funder. Unless written permission is obtained from the sponsor and the funder, the data shall not be provided to any third party in any form, except to authorized representatives of the relevant government regulatory authorities.

13.2 Data Retention

Records to be retained by the investigators include (but are not limited to): study-specific documents, identification logs for all subjects, medical records, temporary media documents (thermal sensitive paper should be copied and certified), raw data, all original signed and dated informed consent forms, and copies of responses to queries (including audit records and detailed records of drug disposition to enable assessments or audits from regulatory authorities, the sponsor, the funder, or designated personnel).

In addition, according to Article 80, Chapter VIII of the 2020 GCP issued by the National Medical Products Administration, for clinical trials for drug registration application, the essential documents shall be retained until at least 5 years after the investigational medicinal product is approved for marketing; for clinical trials not for drug registration application, the essential documents shall be retained until at least 5 years after the termination of the clinical trial.

Refer to the clinical study agreement for the sponsor's requirements for record retention. The investigators should contact and receive written approval from the sponsor before disposing of any such documents.

14.0 QUALITY CONTROL AND QUALITY ASSURANCE

14.1 Study Monitoring Visits

Monitoring visits to the site will be made periodically during the study to ensure that the protocol is strictly followed. Raw data will be reviewed to verify the data entered into the eCRFs. Raw data are defined as source documents, data, and records. The investigators and study sites will ensure access to raw data by the sponsor, the funder, or designated personnel and IRB or IEC.

The sponsor or designated personnel will review all aspects of the study including, but not limited to, the investigator's binder, the investigational medicinal products, patient medical records, informed consent documents, eCRFs, and associated source data. It is important that the investigator and other study personnel will be on site during monitoring visits and have sufficient time allocated for the process.

14.2 Protocol Deviations

The investigator should not deviate from the protocol unless it is necessary to eliminate an immediate hazard to study patients. Should other unforeseen circumstances arise that require deviation from protocol-specified procedures, the investigators should consult with the medical monitor (and IRB or IEC, if needed) to determine the appropriate course of action. The inclusion or exclusion criteria will not be subject to exemptions.

14.3 Quality Assurance Audits and Official Inspections

The study site may also be subject to quality assurance audits by the sponsor or designated personnel. In this case, the sponsor- or funder-designated auditor will contact the site in advance to arrange an audit visit. The auditor may ask to visit the facilities where laboratory samples are collected and where the drugs are stored and prepared, as well

as any other facilities used during the study. In addition, other regulatory authorities including the NMPA may inspect this study. If the study site is contacted for an inspection by a regulatory authority, it should notify the sponsor and the funder immediately. The investigators and study sites should ensure that the quality assurance auditors have access to all study documents.

15.0 ETHICS

This study will be conducted with the highest respect for the subjects participating in the study according to the agreement, the ethical principles specified in the "Declaration of Helsinki", and the ICH GCP. Each investigator will conduct the study according to applicable local or regional regulatory requirements.

15.1 IRB and/or IEC Approval

The sponsor or designated personnel should submit the relevant documents to the appropriate IRB or IEC for review and approval of the study protocol. The study protocol, Investigator's Brochure, copy of informed consent form, patient recruitment materials and/or advertisements, and other documents required by all applicable laws and regulations must be submitted to the IRB or IEC for approval. Written approval of the protocol and patient informed consent from the IRB or IEC must be obtained and submitted to the sponsor and the funder prior to study initiation. The IRB or IEC approval must indicate the exact title, number, and version/date of the protocol, the version identification numbers of other documents (e.g., informed consent form), as well as the approval date. The study sites must comply with all requirements stipulated by the appropriate IRB or IEC. This may include notification to the IRB or IEC of amendments to the protocol, updates to the informed consent form, recruitment materials intended for viewing by patients, local safety reporting requirements, reports and updates of study reviews conducted at intervals designated by the appropriate IRB or IEC, and submission of final status reports to the IRB or IEC. All IRB and IEC approvals and relevant documentation for the above items must be provided to the sponsor and the funder.

15.2 Subject Information, Informed Consent, and Subject Authorization

The informed consent form will embody the elements of informed consent as described in the "Declaration of Helsinki" and "ICH GCP" and will comply with all relevant laws and regulations. Records describing the permitted use, transfer, and disclosure of the patient's personal information and health information in the study will be provided in the informed consent form. The informed consent form will explain the nature, objectives, and potential risks and benefits of the study, as well as the date on which informed consent is given. The informed consent form will detail the requirements for subjects' participation in the study and the fact that the subjects are free to withdraw at any time without giving a reason and without prejudice to their further medical care.

The informed consent form must be approved by both the IRB or IEC and the sponsor before use. The informed consent form must be written in a language fully comprehensible to patients. It is the responsibility of the investigator to explain the detailed elements of the informed consent form to patients. Information should be given orally and in writing whenever possible and in a manner deemed appropriate by the IRB or IEC. If a patient is unable to provide sufficient written informed consent, a legally acceptable representative of the patient may provide such consent on behalf of the patient, in accordance with applicable laws and regulations. The investigators must sign and date the informed consent form; however, the sponsor may allow the designated personnel of the investigators to sign the informed consent form to the extent permitted by applicable laws.

Once signed, the original informed consent form will be retained in the investigator's binder. The investigators must record in the patient's medical record the date when the patient signs the informed consent form. A copy of the signed informed consent form should be provided to the patient.

All revised informed consent forms must be reviewed and signed in the same manner as the original informed consent form, by the patient or a legally acceptable representative of the patient involved. The date the revised consent form is obtained should be recorded in the patient's medical record, and the patient should receive a copy of the revised informed consent form.

15.3 Privacy Protection

The sponsor and designated personnel acknowledge and uphold the principle of protection of patient privacy. Throughout the study, the patient's source data will be linked to the sponsor and the funder's clinical study database or documentation by a unique identification number only. Limited patient information (e.g., sex, age, or date of birth) and patient initials are allowed to be used to confirm the accuracy of the patient's identity and unique identification number, as permitted by all applicable laws and regulations.

To comply with ICH GCP and to verify compliance with this protocol, as required by the sponsor, the investigator should permit the sponsor's monitor or designated monitor, representative from any regulatory authority, the sponsor-designated auditor, and the appropriate IRB and IEC to review patients' original medical records (source data or documents), including, but not limited to, laboratory test reports, ECG reports, records of admission and

discharge that occur during patients' participation in the study, autopsy reports, etc. As part of the informed consent process, obtaining patients' original medical records requires specific authorization from the patients.

Copies of source documents for any patient provided to the sponsor must have certain personal identity information removed (i.e., patient name, address, and other identification fields not collected on the patient's eCRF).

15.4 Publication, Disclosure, and Clinical Trial Registration

15.4.1 Publication and disclosure

All publications and presentations must be prepared in accordance with the terms in this section and the sponsor's agreement.

15.4.2 Clinical study registration

The sponsor or its designated personnel will register the clinical trial on a publicly accessible website prior to trial initiation to ensure timely accessibility of clinical trial information to the public and compliance with applicable laws, regulations, and guidelines.

15.4.3 Disclosure of clinical study results

The sponsor or its designated personnel will post the results of the clinical trial on a publicly accessible website as required by applicable laws and/or regulations.

15.5 Insurance and Compensation

Each patient in the study can obtain clinical study observation insurance provided by the sponsor. Please refer to the insurance contract for the policy regarding patient compensation and treatment of injury. If investigators have any questions regarding this policy, they should contact the sponsor or the study operation team.

16.0 MEDICAL EMERGENCIES AND CONTACT INFORMATION OF THE SPONSOR OR ITS DESIGNATED PERSONNEL

The principal investigator is responsible for ensuring that procedures and expertise are in place during the study to manage medical emergencies. Medical emergencies usually constitute SAEs and should be reported.

In medical emergencies, the investigators may contact the study team's safety physician. If the study team's physician is not available, please contact the sponsor's safety physician. The contact information of the sponsor and its designated personnel is noted in the table below.

Name	Role in the study	Address and phone number
Yun Fan	Sponsor's study team member AE/SAE<br><br>24-hour emergency contact	No. 38 Guangji Road, Banshan Bridge, Gongshu District, Hangzhou, Zhejiang, China<br>Mobile: +86-13858182310<br>E-mail: fanyun@zjcc.org.cn
Kun Liu	Project management team<br><br>24-hour emergency contact	Building 16, No. 989 Dongfang Road, Pudong New Area, Shanghai, China<br>Mobile: +86-18652100216<br>Email: liuk6@hspharm.com

17.0 REFERENCES

1. Pisters KM, Le Chevalier T. Adjuvant chemotherapy in completely resected non-small-cell lung cancer. J Clin Oncol. 2005, 23(14):3270-8.
2. Bonomi PD. Implications of key trials in advanced nonsmall cell lung cancer. Cancer. 2010, 116(5):1155-64.
3. Gahr S, Stoehr R, Geissinger E, et al. EGFR mutational status in a large series of Caucasian European NSCLC patients: data from daily practice. Br J Cancer 2013;109:1821-8.
4. Maemondo M, Inoue A, Kobayashi K, et al. Gefitinib or chemotherapy for non-small-cell lung cancer with mutated EGFR. N Engl J Med 2010;362:2380-8.
5. Burtness B, Anadkat M, Basti S, et al. NCCN Task Force Report: Management of dermatologic and other toxicities associated with EGFR inhibition in patients with cancer. J Natl Compr Canc Netw 2009;7(suppl 1):S5- 24.
6. Kobayashi S, Boggon TJ, Dayaram T, et al. EGFR mutation and resistance of non-small-cell lung cancer to gefitinib. N Engl J Med 2005;3:786-92.
7. Su KY, Chen HY, Li KC, et al. Pretreatment epidermal growth factor receptor (EGFR) T790M mutation predicts shorter EGFR tyrosine kinase inhibitor response duration in patients with non-small-cell lung cancer. J Clin Oncol. 2012;30(4):433-40.
8. Rangachari D, Yamaguchi N, VanderLaan PA, et al. Brain metastases in patients with EGFR-mutated or ALK-rearranged non-small-cell lung cancers. Lung Cancer. 2015;88(1):108-111.
9. Hsu F, De Caluwe A, Anderson D, Nichol A, Toriumi T, Ho C. EGFR mutation status on brain metastases from non-small cell lung cancer. Lung Cancer. 2016; 96:101-107.
10. Matsumoto S, Takahashi K, Iwakawa R, et al. Frequent EGFR mutations in brain metastases of lung adenocarcinoma. Int J Cancer. 2006;119(6):1491-1494.
11. Barajas RF Jr, Cha S. Imaging diagnosis of brain metastasis. Prog Neurol Surg. 2012; 25:55-73.
12. Lynch TJ, Bell DW, Sordella R, et al.: Activating mutations in the epidermal growth factor receptor underlying responsiveness of non small cell lung cancer to Gefitinib. N Engl J Med 2004; 350:2129-2139.
13. Tanaka T, Matsuoka M, Sutani A, et al.: Frequency of and variables associated with the EGFR mutation and its subtypes. Int J Cancer 2010; 126:651-655.
14. Paez JG, Janne PA, Lee JC, et al.: EGFR mutations in lung cancer: correlation with clinical response to Gefitinib therapy. Science 2004; 304:1497-1500.
15. Sakurada A, Shepherd FA, Tsao MS: Epidermal growth factor receptor tyrosine kinase inhibitors in lung cancer: impact of primary or secondary mutations. Clin Lung Cancer 2006;7(suppl 4): S138–S144
16. Grommes C, Oxnard GR, Kris MG, Miller VA, Pao W, Holodny AI, Clarke JL, Lassman AB. "Pulsatile" high-dose weekly SoC EGFR-TKI Erlotinib or Gefitinib for CNS metastases from EGFR mutant non-small cell lung cancer. Neuro Oncol 2011; 13(12):1364-9.
17. Zhao J, Chen M, Zhong W, et al. Cerebrospinal fluid concentrations of gefitinib in patients with lung adenocarcinoma. Clin Lung Cancer. 2013;14(2):188-193.
18. Umemura S, Tsubouchi K, Yoshioka H, et al. Clinical outcome in patients with leptomeningeal metastasis from non-small cell lung cancer: Okayama Lung Cancer Study Group. Lung Cancer. 2012;77(1):134-139.
19. Mok TS, Wu Y-L, Ahn M-J, et al. Osimertinib or Platinum-Pemetrexed in EGFR T790M-Positive Lung Cancer. N Engl J Med. 2017;376(7):629-640.
20. Bao R. HS-10296 (in vitro). Preclinical Pharmacology of HS-10296. Jiangsu Hansoh Pharmaceutical Group; 02 February 2016.
21. Gao W. CPB-P15-5267. Compound Screening by Caliper Assay. Shanghai ChemPartner Co., Ltd.; 20 August 2015.
22. Bao R. HS-10296 (in vivo). Preclinical Pharmacology of HS-10296. Jiangsu Hansoh Pharmaceutical Co., Ltd.; 02 February 2016.
23. Zhang H. O14-S145-SP. Effect of HS-10296 on the hERG currents. JOINN Laboratories (Suzhou); 01 September 2015.
24. Niu X. RP-HS 10296-ADME-1_PPB. Determination of the in vitro binding of HS-10296 and its metabolite HAS-719 to plasma proteins in the rat, dog, and human species. HQ Bioscience Co., Ltd.; 20 January 2016.
25. Zhang T. R14-S145-SP. Effect of HS-10296 on central nervous system functions in Sprague-Dawley rats after a single oral gavage administration. JOINN Laboratories (Suzhou); 17 December 2015.
26. Zhang T. D14-S145-SP. Effects of HS-10296 on cardiovascular and respiratory functions in conscious beagle dogs following oral gavage administration. JOINN Laboratories (Suzhou); 10 December 2015.
27. Niu X. RP-HS 10296-ADME-1_RPK. Pharmacokinetics of HS-10296 in Sprague-Dawley rats following oral and intravenous administration at different dose levels. HQ Bioscience Co., Ltd.; 20 January 2016.

28. Niu X. RP-HS 10296-ADME-1_DPK. Pharmacokinetics of HS-10296 in beagle dogs following oral and intravenous administration at different dose levels. HQ Bioscience Co., Ltd.; 20 January 2016.
29. Niu X. RP-HS 10296-ADME-1_RTD. Tissue distribution evaluation of HS-10296 in Sprague-Dawley rats following oral administration. HQ Bioscience Co., Ltd.; 20 January 2016.
30. Li L. RP-HS 10296-ADME-1_ME. Metabolism and excretion of HS-10296 in Sprague-Dawley rats and dogs following oral administration. Shanghai Institute of Materia Medica, Chinese Academy of Sciences; 20 January 2016
31. Zhou J. RP-HS 10296-ADME-1_MS. Metabolic stability evaluation of HS-10296 in liver microsomes. Shanghai Institute of Materia Medica, Chinese Academy of Sciences; 20 January 2016.
32. Li L. RP-HS10296-ADME-1_Species Differences. Metabolic profiles of HS-10296 in hepatocyte_species differences in HS-10296 metabolism. Shanghai Institute of Materia Medica, Chinese Academy of Sciences; 20 January 2016.
33. Li L. RP-HS10296-ADME-1_phenotyping, In vitro metabolic profiles in human liver microsomes and CYP450 phenotyping of HS-10296. Shanghai Institute of Materia Medica, Chinese Academy of Sciences, 20 January 2016.
34. Zhou J. RP-HS10296-ADME-1_inhib. Evaluation of HS-10296 for P450 in vitro inhibition potential. Shanghai Institute of Materia Medica, Chinese Academy of Sciences, 20 January 2016.
35. Guo Z. RP-HS10296-ADME-1_DDI. Evaluation of HS-10296 for in vitro induction potential. Shanghai Institute of Materia Medica, Chinese Academy of Sciences; 20 January 2016.
36. Guo Z. RP-HS10296-ADME-1_PB. Permeability evaluation of HS-10296 in Caco-2 cells. HQ Bioscience Co., Ltd., 20 January 2016.
37. Cao J. R14-S145-SD. Single dose toxicity study of HS-10296 in Sprague-Dawley rats by oral gavage. JOINN Laboratories (Suzhou); 10 December 2015.
38. Cao J. D14-S145-SD. Single dose toxicity study of HS-10296 in Beagle dogs by oral gavage. JOINN Laboratories (Suzhou); 13 November 2015.
39. Cao J. R14-S145-RD. A 13-week toxicity study of HS-10296 by oral gavage to Sprague-Dawley rats with a 4-week recovery period. JOINN Laboratories (Suzhou); 31 December 2015.
40. Cao J. R14-S145-RD. A 13-week toxicity study of HS-10296 by oral gavage to Sprague-Dawley rats with a 4-week recovery period (Amended Version). JOINN Laboratories (Suzhou); 20 February 2017.
41. Cao J. R14-S145-RD. A 13-week toxicity study of HS-10296 by oral gavage to Sprague-Dawley rats with a 4-week recovery period; Report Amendment 2. JOINN Laboratories (Suzhou); 28 February 2017.
42. Cao J. D14-S145-RD. A 13-week toxicity study of HS-10296 by oral gavage to Beagle dogs with a 4-week recovery period. JOINN Laboratories (Suzhou); 31 December 2015.
43. Cao J. D14-S145-RD. A 13-week toxicity study of HS-10296 by oral gavage to Beagle dogs with a 4-week recovery period (Amended Version). JOINN Laboratories (Suzhou); 20 February 2017.
44. Brown AP, Dunstan RW, Courtney CL, et al. Cutaneous lesions in the rat following administration of an irreversible inhibitor of erbB receptors, including the epidermal growth factor receptor. Toxicol Pathol. 2008;36:410-19.
45. Gopinath C, Mowat V. The musculoskeletal system and skin. In: Atlas of Toxicological Pathology. New York: Humana Press. 2014; 229-52.
46. Chanprapaph K, Vachiramon V, Rattanakaemakorn P. Epidermal growth factor receptor inhibitors: a review of cutaneous adverse events and management. Dermatol Res Pract. 2014, Article ID 734249, 8 pages. http://ds.doi.org/: 10.1155/2014/734249. Epub 2014 Mar 2.
47. Greaves P. Digestive system. In: Histopathology of Preclinical Toxicity Studies: Interpretation and Relevance in Drug Safety Evaluation. London: Academic Press. 2012;325-431.
48. Gopinath C, Mowat V. The alimentary system and pancreas. In: Atlas of Toxicological Pathology. New York: Humana Press. 2014;77-107.
49. Gokulgandhi M, Vadlapudi AD, Mitra AK. Ocular toxicity from systemically administered xenobiotics. Expert Opin Drug Metab Toxicol. 2012;8(10):1277-91.
50. Hager T, Seitz B. Ocular side effects of biological agents in oncology: what should the clinician be aware of? Onco Targets Ther. 2014;7:69-77.
51. Rudman DG, Foley GL. Female reproductive system. In: Handbook of Toxicologic Pathology, Vol. 3. Hashcheck WM, Rousseaux CG, Wallig MA, eds. New York: Elsevier Inc. 2013;2599-2663.
52. Rudmann DG, Cardiff R, Chouinard L, et al. Proliferative and nonproliferative lesions of the rat and mouse mammary, Zymbal's, preputial, and clitoral glands. Toxicol Pathol. 2012;40:7S-39S.

53. Everds NE, Snyder PW, Bailey KL, et al. Interpreting stress responses during routine toxicity studies: a review of the biology, impact, and assessment. Toxicol Pathol. 2013;41:560-614.
54. Hao Z. O14-S145-AM. A mutagenicity test of HS-10296 in Salmonella Typhimurium. JOINN Laboratories (Suzhou); 23 October 2015.
55. Hao Z. O14-S145-CA. Chromosomal aberration study of HS-10296 in Chinese hamster lung fibroblast. JOINN Laboratories (Suzhou); 30 October 2015.
56. Hao Z. M14-S145-MN. Micronucleus study of HS-10296 using bone marrow in ICR mice with single dose by oral gavage. JOINN Laboratories (Suzhou); 23 October 2015.
57. Ge H. R14-S145-IRP. Male and female fertility and early embryonic developmental toxicity study of HS-10296 administered by oral gavage to Sprague-Dawley rats. . JOINN Laboratories (Suzhou); 15 December 2015.
58. Ge H. R14-S145-2RP. Embryo-fetal developmental toxicity study of HS-10296 administered by oral gavage to Sprague-Dawley rats. JOINN Laboratories (Suzhou); 15 December 2015.
59. Porta R, Sánchez-Torres JM, Paz-Ares L, et al. Brain metastases from lung cancer responding to erlotinib: the importance of EGFR mutation. Eur Respir J. 2011, 37(3):624-31.
60. Bai H, Han B. The effectiveness of erlotinib against brain metastases in non-small cell lung cancer patients. Am J Clin Oncol. 2013, 36(2): 110-5.

Appendix A Investigators' Informed Consent to Use of Personal Information

The sponsor will collect and retain personal information on the investigators, including names, addresses, and other personal identity information. In addition, the investigators' personal information may be obtained by the following entities or institutions:

- The sponsor, its affiliates, and licensed partners
- Business partners supporting the sponsor, its affiliates, and licensed partners
- Regulatory authorities and other health authorities
- IRBs and IECs

Investigators' personal information may be retained and processed by the sponsor and other parties as appropriate for research purposes, including:

- Assessment of the suitability of the investigators for the study and/or other clinical studies
- Management, supervision, inspection, and audit of studies
- Analysis, review, and validation of study results
- Study-related safety reports and pharmacovigilance
- Preparation and submission of study-related submission dossiers to regulatory authorities, and related correspondence and communications
- Preparation and submission of submission dossiers related to other drugs and their clinical studies to regulatory authorities, and related correspondence and communications
- Regulatory inspections and investigations of the study
- Self-inspection and internal review by the sponsor, its affiliates, and licensed partners
- Filing and auditing of study records
- Publication of site contact information, study details, and results on publicly accessible clinical trial registries, databases, and websites

The investigators' personal information may be transferred to other countries/regions without data protection laws. In such cases, data protection measures equivalent to those in the investigators' countries/regions will be provided. The investigators acknowledge and consent to the use of their personal information by the sponsor and other parties for the purposes described above, whether they are individuals or entities.

Appendix B Study Schedule

Visit[a]	Screening period	Treatment period			EOT follow-up	Follow-up period		
	1	2	3	4+		28-Day follow-up[b]	Progression follow-up Once every 8 weeks	Survival follow-up Once every 12 weeks
**Treatment cycle[c]/Day**		**C1 D1**	**C2 D1**	**Every 8 weeks in C4 and thereafter D1**	**NA**	**NA**	**NA**	**NA**
**Day**	**-28**	**1**	**29**	**85+**	**NA**	**NA**	**NA**	**NA**
**Window period (days)**	**NA**	**0**	**±7**	**±7**	**+7**	**+7**	**±7**	**±7**
Informed consent	X							
Demographic and other baseline characteristics	X							
Medical/surgical history	X							
Inclusion/exclusion criteria	X							
EGFR mutation test	X	X	X					
Physical examination (including weight)[d]	X	X	X	X	X			
Height	X							
ECOG PS score	X	X	X	X	X			
Pregnancy test	X							
Ophthalmological examination	When clinically indicated							
Vital signs[d]	X	X	X	X	X			
Clinical biochemistry/hematology/urinalysis[d]	X	X	X	X	X			
Blood sampling[f]		X	X	Once within one week after PD				
Histopathological examination[g]		X		Once within one week after PD				
ECG[h]	X	X	X	X	X			
Echocardiography (LVEF)	X	When clinically indicated			X			
Tumor assessment (RECIST v1.1)[i]	X	X	X	Every 8 weeks ± 7 days				
Drug dispensing		X	X	X				

Administration		Daily administration						
Concomitant medications	←→	←→	←→	←→	←→	←→	X Occurring before completion of the 28-day follow-up	
Adverse event	←→	←→	←→	←→	←→	←→		
Anti-tumor treatment	X						X	X
Subsequent response/progression data								X
Survival status								X
Comments	a. Subjects will be followed up at Week 4, Week 12, and every 8 weeks thereafter, and the efficacy should be confirmed as soon as possible for patients with PR/CR at Week 4.<br>b. Subjects should complete the visit procedures within 28 days (+7 days) after discontinuation of IMP, and ongoing and new AEs should be followed up. All AEs should be followed up until recovery or returning to the baseline level, or loss to follow-up or death of the subject. After the end of the safety follow-up period, only SAEs considered related to aumolertinib or study procedures will be collected.<br>c. A cycle is defined as 28 consecutive days of treatment.<br>d. At baseline, this evaluation should be completed before administration on the visit day. If the screening examinations are completed within 7 days before the first dose of the IMP, they can be exempted on C1D1.<br>e. Venous blood sampling: Blood samples will be collected at the following points: within ± 7 days before C1D1 and C2D1 administration, and within 1 week after PD (24 h after the previous dose).<br>f. Tumor tissue slice collection: 10–15 histopathological slices need to be collected before C1D1 administration and within one week after PD (24 h after the previous dose)<br>g. An ECG will be performed at the time of any cardiac-related AE.<br>h. Baseline examinations should be performed within 28 days prior to the first dose. After the start of treatment, subjects will be evaluated once every 8 weeks until PD is confirmed as per RECIST 1.1, even if the subjects discontinue the treatment or receive other anti-tumor treatments before PD. Tumor assessment data will be obtained from CT or MRI (contrast-enhanced CT or MRI is recommended as the preferred method).							

Appendix C Actions Required for Concomitant Elevations of Aminotransferases and Total Bilirubin - Hy's Law

1. Introduction

The investigators should remain vigilant for elevations in liver chemistry values during the study. The investigators are responsible for determining whether a patient meets potential Hy's Law (PHL) criteria at any point during the study. The investigators, together with the sponsor's clinical project representative, will review and assess if a potential case meets the Hy's Law (HL) criteria. Hy's law (HL) criteria are met if there is no alternative explanation for the liver biochemistry elevations other than drug-induced liver injury (DILI) caused by the investigational medicinal product (IMP).

The investigators are responsible for recording data related to PHL/HL cases and reporting adverse events (AEs) and serious adverse events (SAEs) according to standard safety reporting procedures.

2. Definition

2.1 PHL

Aspartate aminotransferase (AST) or alanine aminotransferase (ALT) ≥ 3 × upper limit of normal (ULN) and total bilirubin (TBiL) ≥ 2 × ULN at any time point during the study, regardless of increases in alkaline phosphatase (ALP). The elevations do not have to occur at the same time or within a specified time frame.

2.2 HL

AST or ALT ≥ 3 × ULN and TBiL ≥ 2 × ULN with no alternative explanation for the concomitant elevations other than IMP, such as elevated ALP indicating cholestasis, viral hepatitis, and another drug. The elevations do not have to occur at the same time or within a specified time frame.

3. Identification of PHL Cases

In order to identify PHL cases, it is important to perform a comprehensive review of laboratory data for patients who meet any of the following individual or combination identification criteria:

a. ALT ≥ 3 × ULN
b. AST ≥ 3 × ULN
c. TBiL ≥ 2 × ULN

The investigators will immediately review each new laboratory report and, if it meets the criteria, will:

a. Notify the sponsor's representative;
b. Determine if a patient meets PHL criteria (see Section 2 for definition) by reviewing all previously accessed laboratory reports;
c. Enter the laboratory data into the laboratory CRF immediately.

4. Follow-Up

4.1 PHL criteria not met

If a patient does not meet PHL criteria, the investigator will:

a. Notify the sponsor's representative that the patient does not meet PHL criteria.
b. Follow up subsequent laboratory results according to the guidance provided in the clinical study protocol.

4.2 PHL criteria met

If a patient meets PHL criteria, the investigator will:

a. Determine if PHL criteria are met prior to starting study treatment in the presence of liver metastases (see Section 6).
b. Notify the sponsor's representative, who will inform the central study team.

The study doctor will contact the investigator for guidance and to discuss and agree on a method for follow-up of study patients and continuous review of data. Following this contact, the investigator will:

a. Monitor the patient until liver biochemistry parameters and appropriate clinical symptoms and signs return to normal or baseline levels, or as medically indicated.
b. Investigate the etiology of the event and discuss with the study doctor to perform a diagnostic investigation.
c. Complete the three liver CRF modules as information becomes available.

If at any time (in consultation with the study doctor), a PHL case meets the criteria for seriousness, report it as an SAE following standard procedures.

5. Review and Assessment of HL Cases

For all cases where PHL criteria are met, the instructions in this section should be followed. No more than 3 weeks

after the biochemical abnormalities are initially detected, the study doctor will contact the investigator to review the available data and agree on whether there is an alternative explanation for DILI other than that caused by the IMP. The sponsor's medical science director and safety physician, as well as other subject matter experts, will also be involved in this review, as appropriate.

Based on the results of the review and assessment, the investigator will follow the instructions below. If there is an agreed alternative explanation for the ALT or AST and TBiL elevations, determine if the alternative explanation is an AE and then determine if the AE meets the criteria for an SAE:

a. If the alternative explanation is not an AE, please record the alternative explanation on the appropriate CRF.

b. If the alternative explanation is an AE/SAE, record the AE/SAE in the CRF accordingly and follow the sponsor's standard procedures.

If there is no alternative explanation for the ALT or AST and TBiL elevations other than the IMP, report the SAE according to the sponsor's standard procedures (report term "Hy's Law"):

a. If no other seriousness criteria apply, the "medically important" seriousness criteria should be used.

b. As there is no alternative explanation for the HL case, a causality assessment of "related" should be assigned.

If a delay of more than 3 weeks is unavoidable before obtaining the information necessary to assess whether a case meets the HL criteria, then it is assumed that there is no alternative explanation before making an informed decision:

a. Report SAEs (report term "potential Hy's Law") using strict criteria and causality assessment.

b. Continue follow-up and review according to the agreed plan. Once the necessary supplementary information is obtained, the review and assessment will be repeated to determine whether the HL criteria are met. The SAE report will be updated based on the review results.

6. Actions Required When PHL Criteria Are Met Before and After Starting Study Treatment

This section applies to patients who meet PHL criteria at the time of study treatment or have already met PHL criteria at a study visit prior to starting study treatment. The first occurrence of PHL at the time of study treatment, even if there is no significant change in the patient's condition from the pre-study treatment visit, the investigator will:

a. Notify the sponsor's representative, who will inform the central study team.

b. Follow the subsequent process as described in Section 4.2.

A "significant" change in a patient's condition refers to a clinically relevant change in any liver biochemical parameter (ALT, AST, or total bilirubin), either individually or in combination, or any clinically relevant change in associated symptoms. Determination of significant changes rests with the investigator. If there is any uncertainty, consult the study doctor.

7. Actions Required for PHL Recurrence

This section applies to patients who meet PHL criteria at the time of study treatment and have already met PHL criteria at a previous study treatment visit. Follow-up, review, and assessment of repeated PHL will be performed based on other causes of previous PHL. The investigator should determine why PHL criteria were previously met and answer the following questions:

a. Is the alternate cause of the prior PHL consistent with chronic or progressive malignant disease or did the patient meet PHL criteria prior to starting study treatment and at the first study treatment visit as described in Section 6?

b. **If No**: Follow the process described in Section 4.2.

c. **If Yes**:

- Determine if there has been a significant change in the patient's condition compared to when PHL criteria were previously met.
- If there is no significant change, no action is required.
- If there is a significant change, follow the process described in Section 4.2.

A "significant" change in a patient's condition refers to a clinically relevant change in any individual liver biochemical parameter (ALT, AST, or total bilirubin), either individually or in combination, or any clinically relevant change in associated symptoms. Determination of significant changes rests with the investigator. If there is any uncertainty, consult the study doctor.

8. Reference

Food and Drug Administration (FDA): Guidance for industry. Drug-induced liver injury: premarketing clinical evaluation. Silver Spring, MD: July 2009.

Appendix D Evaluation of Objective Tumor Response Using Response Evaluation Criteria in Solid Tumors Guidelines 1.1 (RECIST 1.1)

1. Introduction

This appendix details how tumor burden (including specific assessments in this study protocol) will be assessed using Response Evaluation Criteria in Solid Tumors Guidelines 1.1 (RECIST 1.1) (Eisenhauer et al., 2009) in the trial.

2. Definitions of Measurable, Non-Measurable, Target, and Non-Target Lesions

Patients with at least 1 measurable lesion will be accurately assessed at baseline by computed tomography (CT), magnetic resonance imaging (MRI), or general X-ray.

2.1 Measurable lesions

At least 1 non-irradiated lesion with the longest diameter of ≥ 10 mm (except for lymph nodes, which must have the short axis of ≥ 15 mm) that can be accurately measured at baseline and can be accurately remeasured by CT or MRI.

2.2 Non-measurable lesions

a. All other lesions, including small lesions (pathological lymph nodes with the longest diameter of < 10 mm or the short axis of ≥ 10 mm to < 15 mm at baseline; nodules with the short axis of < 10 mm are considered non-pathological and should not be recorded as non-target lesions [NTLs]).

b. Truly non-measurable lesions include the following: bone lesions, submeningeal disorders, ascites, pleural/pericardial effusion, inflammatory breast diseases, lymphangitis of the skin or lung, and abdominal masses/abdominal organomegaly identified by physical examination, which cannot be measured by CT or MRI.

c. Previously irradiated lesions as local post-radiation changes that may affect the size of the lesion. Therefore, previously irradiated lesions will not be considered measurable and should be selected as NTLs at baseline and followed up as part of NTL assessment.

d. Skin lesions in clinical examination.

2.3 Special considerations

a. Lytic bone lesions or mixed lytic-blastic lesions with identifiable soft tissue components may be considered measurable if the soft tissue component meets the definition of measurability. Vitreous injury is considered non-measurable.

b. Cystic metastatic tumors can be considered as measurable lesions if they meet the criteria for measurability from a radiological point of view. However, if non-cystic lesions exist in the same patient, these non-cystic lesions should be selected as target lesions (TLs).

2.4 Target lesions (TLs)

Up to 5 **measurable** lesions (up to 2 lesions per organ) representing all involved lesions suitable for accurate re-measurement should be identified as baseline TLs.

2.5 Non-target lesions (NTLs)

All other lesions (or sites of disease) not recorded as TLs should be identified as NTLs at baseline.

3. Measurement Method

The same assessment method and the same technique should be used to characterize each identified and reported lesion at baseline and during follow-up.

The methods used for RECIST assessment are summarized in the table below, and the tumor assessment methods that cannot be used in this trial are discussed below with a justification.

Table 1. Summary of assessment methods

Target lesion	Non-target lesions	New lesions
CT (preferred)<br>MRI	CT (preferred)<br>MRI<br>Clinical examination<br>X-ray, chest X-ray	CT (preferred)<br>MRI<br>Clinical examination<br>X-ray, chest X-ray<br>Ultrasound<br>Bone scan<br>FDG-PET

3.1 CT and MRI

CT and MRI are generally considered as the best currently available and reproducible methods for measuring

response of TLs, assessment of NTLs, and identification of new lesions.

In this study, CT examinations of the chest and abdomen are recommended to assess tumor burden at baseline and follow-up. CT with intravenous (IV) contrast is the preferred method. MRI should be used when CT is not feasible or medically contraindicated. For the assessment of brain injury, MRI is the preferred method.

3.2 Clinical examination

Clinical examination will not be used for the assessment of TLs. Clinically detected lesions may be selected as TLs if they are subsequently assessed by CT or MRI scans. Clinical examination can be used to assess NTLs and also to identify new lesions. However, these patients also need to have other lesions that can be assessed by CT, MRI, or general X-ray.

3.3 X-ray

3.3.1 General X-ray

General X-ray can be used as a method to assess bone NTLs and to identify new bone lesions.

3.3.2 Chest X-ray

Chest X-ray is not used to assess TLs, which will be assessed by CT or MRI. However, chest X-ray can be used to assess NTLs and identify new lesions.

3.4 Ultrasound

Ultrasound examination is not used to assess TLs and NTLs because it is not a reproducible method, does not provide a precise assessment of tumor size, and it is subjective and operator-dependent. However, ultrasound examination can be used to identify new lesions. If new clinical symptoms appear and ultrasound examination is performed, new lesions should be confirmed by CT or MRI.

3.5 Endoscopy and laparoscopy

Endoscopy and laparoscopy will not be used for tumor assessment as they are not validated in the context of tumor measurement.

3.6 Tumor markers

Tumor markers will not be used for tumor response evaluation according to RECIST 1.1.

3.7 Cytology and histology

Histology will not be used for tumor response evaluation according to RECIST 1.1.

Cytological confirmation of the tumor origin of any effusion that appears or worsens during treatment is required when the measurable tumor meets the criteria for response or stable disease. In such cases, cytology is necessary to distinguish between response/stable disease (effusion may be a side effect of treatment) and progressive disease (if the tumor origin of the effusion is confirmed). In the absence of cytology results, significant worsening (from trace to large amounts) of effusion or the presence of clinically significant effusion (requiring changes in drug therapy) during study treatment will be considered as progression of NTLs or disease progression due to new lesions.

3.8 Isotope bone scan

Bone lesions identified by isotope bone scans at baseline and confirmed by CT, MRI, or X-ray should be recorded as NTLs and followed up by the same method as baseline assessment.

Isotope bone scan can be used as an assessment method to identify new bone lesions at follow-up. Positive hotspots identified by bone scans performed at any time point during the trial (not present on baseline bone scan assessment) will be recorded as new lesions. Investigators should consider the positive hotspot to be an important new site of malignant disease and a representative of true disease progression in order to record new lesions. In case of equivocal findings on bone scan, confirmation by CT, MRI, and X-ray is recommended.

3.9 FDG-PET scan

A fluorodeoxyglucose-positron emission tomography (FDG-PET) scan may be used as a method of identifying new lesions according to the following algorithm: New lesions will be recorded if there is positive FDG uptake (defined as uptake greater than twice that of surrounding tissues) that is not present on the baseline FDG-PET scan; or if the positive FDG uptake corresponds to the site of a new lesion on CT/MRI at the same follow-up. If no baseline FDG-PET scan is available and there is no evidence of new lesions on CT/MRI scans, subsequent CT/MRI assessments should be continued to confirm new lesions as per the protocol or clinical indication.

4. Tumor Response Evaluation

4.1 Evaluation plan

CT examinations of the chest and abdomen (including the liver and adrenal glands) will be used to assess tumor burden at baseline and follow-up. CT with intravenous (IV) contrast is the preferred method. MRI should be used when CT is not feasible or medically contraindicated.

Baseline tumor assessment, which should be performed ≤ 28 days prior to start of study treatment, should involve all

regions in which diseases under assessment are known to metastasize, and potentially involved regions should be additionally assessed based on signs and symptoms of individual patients. Follow-up assessments should be performed every 6 weeks (± 7 days) after the start of treatment until discontinuation of study treatment or withdrawal of informed consent. Imaging examination should also be performed at follow-up if new lesions are suspected at other sites.

If unscheduled assessments are performed and the patient has no progression, every effort should be made to enable the patient to perform subsequent assessments at the scheduled visits. This schedule will be followed in order to minimize any unintentional deviations due to the assessment of some patients at a follow-up frequency different from that for other patients.

4.2 Target lesions

4.2.1 Target lesion recording

Up to 5 measurable lesions (up to 2 lesions per organ, including lymph nodes) representing all involved lesions should be identified as baseline TLs. TLs should be selected on the basis of their size (the longest diameter of non-lymph node lesions or the short axis of lymph node lesions), but in addition should be those that lend themselves to reproducible repeated measurements. It may be the case that, on occasion, the largest lesion does not lend itself to reproducible measurement in which circumstance the next largest lesion which can be measured reproducibly should be selected.

The location of each TL should be recorded, along with the maximum diameter of non-lymph node lesions (or the short axis of lymph node lesions). All measurements should be recorded in millimeters. At baseline, the sum of diameters of all TLs will be calculated and reported as the baseline sum of diameters. At follow-up, the sum of diameters of all TLs will be calculated and reported as the follow-up sum of diameters.

Special cases

a. For TLs that can be measured in 2 or 3 dimensions, the longest diameter will be always reported. For pathological lymph nodes that can be measured in 2 or 3 dimensions, the short axis will be always reported.

b. If the thickness of slices used in CT/MRI is > 5 mm, the minimum size of measurable lesions at baseline should be twice the slice thickness on baseline scan.

c. If the lesion disappears completely, the longest diameter should be recorded as 0 mm.

d. If the TL splits into 2 or more parts, the sum of diameters of these parts should be recorded.

e. If 2 or more TLs are combined, the sum of diameters of the combined lesions should be recorded, and the diameter of other lesions should be recorded as 0 mm.

f. If the TL is believed to be present and is faintly seen but too small to measure, a default value of 5 mm should be assigned. If accurate measurements can be obtained, they should be recorded, even if they are below 5 mm.

g. If the TL is too large to be accurately measured, an estimated value of the lesion size should be given.

h. When any intervention is made to the TL during the study, such as radiotherapy, embolization, and surgery, the size of the TL should still be given where possible.

4.2.2 Evaluation of target lesions

Table 2 provides the criteria used to determine objective tumor response of TLs.

Table 2. Overall response of target lesions

**Complete response (CR)**	Disappearance of all target lesions from baseline. Any pathological lymph nodes selected as TLs must have a short axis of < 10 mm.
**Partial response (PR)**	At least a 30% decrease in the sum of diameters of TLs, taking as reference the baseline sum diameters.
**Stable disease (SD)**	Neither sufficient shrinkage to qualify for PR nor sufficient increase to qualify for PD.
**Progressive disease (PD)**	At least a 20% increase in the sum of diameters of TLs, taking as reference the smallest sum on study (this includes the baseline sum if that is the smallest on study). In addition to the relative increase of 20%, the sum must also demonstrate an absolute increase of at least 5 mm.
**Inevaluable (NE)**	Only relevant TLs are not assessed or not evaluable or have lesion intervention.<br>Note: If the sum of diameters meets the criteria of PD, the lesion is not inevaluable and should be classified as a PD.

4.3 Non-target lesions

4.3.1 Evaluation of non-target lesions

All other lesions (or sites of disease) not recorded as TLs should be identified as NTLs at baseline. These lesions are not required to be measured, but their status should be followed up at subsequent follow-ups. At each visit, the

investigator should document the overall assessment of NTL response. Table 3 provides the criteria used to determine and document the overall response of NTLs at each visit.

Table 3. Overall response of NTLs

**Complete response (CR)**	Disappearance of all NTLs from baseline. All lymph nodes must be non-pathological in size (the short axis < 10 mm).
**Non-CR/Non-PD**	Persistence of one or more NTL(s).
**Progressive disease (PD)**	Unequivocal progression of existing NTLs. An unequivocal progression can be a significant progression in one or several lesions. In all cases, the progression must be clinically significant for the physician to consider treatment alteration or discontinuation.
**Inevaluable (NE)**	Only one or some relevant NTLs are not assessed, and, in the opinion of the investigator, an overall NTL assessment cannot be provided at this visit.

To achieve "unequivocal progression" on the basis of NTLs, there must be an overall level of substantial worsening in non-target disease such that, even in presence of SD or PR in TLs, the overall tumor burden has increased sufficiently to merit discontinuation of therapy. A modest "increase" in the size of one or more NTLs is usually not sufficient to qualify for unequivocal progression status.

4.4 New lesions

Details of any new lesions and the date of assessment will be recorded. If 1 or more new lesions appear, the assessment result is progression.

A lesion identified on a follow-up assessment that is not scanned at baseline is considered a new lesion and will indicate disease progression.

The finding of a new lesion should be unequivocal, i.e., not attributable to differences in scanning technique, change in imaging modality, or findings thought to represent something other than tumor.

If a new lesion is equivocal, for example because of its small size, continued therapy and follow-up evaluation will clarify if it represents truly new disease. If repeat scans confirm there is definitely a new lesion, then progression should be declared using the date of the initial scan.

4.5 Symptomatic deterioration

Symptomatic deterioration is not a descriptor of an objective response: it is a reason for stopping study therapy. Patients with "symptomatic deterioration" requiring discontinuation of study treatment but having no objective evidence of PD should continue to undergo assessments using RECIST 1.1 according to the clinical study protocol until an objective PD is observed.

4.6 Assessment of overall response

The overall response is derived using the algorithm shown in Table 4.

Table 4. Overall response

Target lesion	Non-target lesions	New lesions	Overall response
CR	CR	No	CR
CR	NA	No	CR
CR	Non-CR/Non-PD	No	PR
CR	NE	No	PR
PR	Non-PD	No	PR
SD	Non-PD	No	SD
NE	Non-PD	No	NE
NA	CR	No	CR
NA	Non-CR/Non-PD	No	SD(Non-CR/Non-PD)
NA	NE	No	NE
PD	Any	Yes or No	PD
Any	PD	Yes or No	PD
Any	Any	Yes	PD

NA = Not available (only applicable when this item is not available at baseline); "/" in the table above means "and".

5. Radiographic Imaging Criteria

The following is for clinical studies only. Assessments conducted using standardized protocols for CT and MRI can be compared within the study and between different studies, regardless of where the examination is performed.

5.1 CT scan

CT scans of the chest and abdomen (including the liver and adrenal glands) should be continuous across all anatomical areas of interest.

The most critical CT image acquisition parameters for optimal tumor assessment using RECIST 1.1 are anatomic coverage, contrast administration, slice thickness, and reconstruction interval.

- **Anatomic coverage**

The optimal anatomic coverage for most solid tumors is the chest, abdomen, and pelvis.

Coverage should encompass all areas of known predilection for metastases in the disease under evaluation and should additionally investigate areas that may be involved based on signs and symptoms of individual patients.

Because a lesion later identified in a body part not scanned at baseline would be considered as a new lesion representing disease progression, careful consideration should be given to the extent of imaging coverage at baseline and at subsequent follow-up time points. This will enable better consistency not only of tumor measurements but also identification of new lesions.

- **IV contrast administration**

Optimal visualization and measurement of metastases in solid tumors requires consistent administration (dose and rate) of IV contrast as well as timing of scanning. Typically, most abdominal imaging is performed during the portal venous phase and (optimally) at about the same time frame after injection on each examination. An adequate volume of a suitable contrast agent should be given so that the metastases are demonstrated to the best effect and a consistent method is used on subsequent examinations for any given patient. It is very important that the same technique be used at baseline and on follow-up examinations for a given patient. For patients who develop contraindications to contrast after baseline contrast CT is done, the decision as to whether non-contrast CT or MRI (enhanced or non-enhanced) should be performed should also be based on the tumor type, anatomic location of the disease and should be optimized to allow for comparison to the prior studies if possible. Each case should be discussed with the radiologist to determine if substitution of these other approaches is possible and, if not, the patient should be considered not evaluable from that point forward. Care must be taken in the measurement of TLs on a different modality and interpretation of non-target disease or new lesions, since the same lesion may appear to have a different size using a new modality. Oral contrast is recommended to help visualize and differentiate structures in the abdomen.

If iodinated contrast is medically contraindicated at baseline or at any time point during the trial, the recommended methods are chest CT without contrast and abdominal and pelvic MRI with contrast. If MRI is not available, CT without IV contrast is performed in one of the chest, abdomen, and pelvis. For the assessment of brain lesions, MRI is the preferred method.

- **Slice thickness and reconstruction interval**

It is recommended that CT scans be performed at 5 mm contiguous slice thickness; this guideline presumes a minimum 5 mm thickness in recommendations for measurable lesion definition. Occasionally, institutions may perform medically acceptable scans at slice thicknesses greater than 5 mm. If this occurs, the minimum size of measurable lesions at baseline should be twice the slice thickness of the baseline scans.

Assessments should include all window settings, especially the lung and soft tissue windows in the chest. When measuring lesions, TLs should be re-measured using the same window settings throughout the trial. Assessments should include all images from each examination, not only "selected" images of significant lesions.

5.2 MRI scan

MRI has excellent contrast, spatial, and temporal resolution; however, there are many image acquisition variables involved in MRI, which greatly impact image quality, lesion conspicuity, and measurement. Furthermore, the availability of MRI is variable globally. The modality used at follow-up should be the same as that used at baseline and the lesions should be measured/assessed on the same pulse sequence. Generally, axial imaging of the abdomen and pelvis with T1 and T2 weighted imaging along with gadolinium enhanced imaging should be performed. The field of view, matrix, number of excitations, phase encode steps, and use of fat suppression and fast sequences should be optimized for the specific body part being imaged as well as the scanner utilized. It is beyond the scope of this appendix to prescribe specific MRI pulse sequence parameters for all scanners, body parts, and diseases. Ideally, the same type of scanner should be used and the image acquisition protocol should be followed as closely as possible to prior scans. Body scans should be performed with breath-hold scanning techniques if possible.

For these reasons, CT is chosen as the imaging modality.

5.3 FDG-PET scan

FDG-PET has gained acceptance as a valuable tool for detecting, staging and restaging several malignancies. If FDG-PET scans are included in a protocol, by consensus, an FDG uptake period of 60 min prior to imaging has been decided as the most appropriate for imaging of patients with malignancy. Whole-body acquisition is important

since this allows for sampling of all areas of interest and can assess if new lesions have appeared thus determining the possibility of interval progression of the disease. Images from the base of the skull to the level of the mid-thigh should be obtained 60 min post injection. PET camera specifications are variable and manufacturer specific, so every attempt should be made to use the same scanner, or the same model scanner, for serial scans on the same patient. Whole-body acquisitions can be performed in either 2- or 3-dimensional mode with attenuation correction, but the method chosen should be consistent across all patients and serial scans in the clinical trial.

5.3.1 PET/CT scan

At present, low dose or attenuation correction CT portions of a combined PET–CT are of limited use in anatomically based efficacy assessments and it is therefore suggested that they should not be substituted for dedicated diagnostic contrast enhanced CT scans for anatomically based RECIST 1.1 measurements. In exceptional cases, if a study site can document that the CT performed as part of a PET-CT is of identical diagnostic quality to a diagnostic CT (with IV and oral contrast) then the CT portion of the PET/CT can be used for RECIST measurements. Note, however, that the PET portion of the CT introduces additional data which may bias an investigator if it is not routinely or serially performed.

6. Reference

Eisenhauer EA, Therasse P, Bogaerts J, Schwartz LH, Sargent D, Ford R, et al New response evaluation criteria in solid tumours: Revised RECIST guideline (version 1.1). Eur J Cancer. 2009;45:228-47.

Appendix E. List of Medications to Be Avoided (Including but Not Limited to the Following)

- Patients must discontinue these medications according to the time window specified below before using the investigational medical product.
- These drugs shall be avoided throughout the trial and for 28 days after discontinuation of the investigational medicinal product, except for topical use (e.g., skin application, inhaled spray, and eye drops).
- If clinically indicated for the treatment of an adverse event, a patient may take any medication.
- Glucocorticoids: Long-term systemic use of glucocorticoids is not recommended for palliative or supportive care purposes. Short-term use of glucocorticoids may be permitted for the treatment of non-autoimmune diseases (e.g., delayed allergic reactions caused by contact allergens) in individual subjects following discussion with the sponsor.

■ **Drugs known to prolong the QT interval**

Drug name	Discontinuation time prior to aumolertinib
Clarithromycin, droperidol, erythromycin, procainamide, levofloxacin, agrylin**agrylin**, ciprofloxacin, cocaine, ondansetron, papaverine hydrochloride, sulpiride, terfenadine, and terlipressin	2 days
Cisapride, disopyramide, dofetilide, domperidone, ibutilide, quinidine, sotalol, sparfloxacin, thioridazine, cilostazol, flecainide, gatifloxacin, grepafloxacin, ibutilide, moxifloxacin, oxaliplatin, propofol, roxithromycin, and sevoflurane	7 days
Bepridil, chlorpromazine, halofantrine, haloperidol, mesoridazine, azithromycin, citalopram, chlorpromazine, dronedarone, escitalopram, fluconazole, levomepromazine, and levosulpiride	14 days
Donepezil and terodiline	3 weeks
Levomethadyl, methadone, pimozide, astemizole, probucol, and vandetanib	4 weeks
Arsenic trioxide and ibogaine	6 weeks
Pentamidine	8 weeks
Amiodarone and chloroquine	1 year

■ **Known sensitive substrates, strong inhibitors, and inducers of CYP3A4**

CYP3A4 strong inducers

Carbamazepine, phenytoin, rifampin, St. John's wort, and dexamethasone; dexamethasone may be used for supportive treatment if needed at the investigator's discretion, but should be used for ≤ 7 consecutive days

CYP3A4 strong inhibitors

Boceprevir, clarithromycin, conivaptan, grapefruit juice, indinavir, itraconazole, ketoconazole, lopinavir/ritonavir, nefazodone, nelfinavir, posaconazole, ritonavir, saquinavir, telaprevir, telithromycin, and voriconazole

CYP3A4 sensitive substrates

Alfentanil, aprepitant, budesonide, buspirone, conivaptan, darifenacin, darunavir, dasatinib, dronedarone, eletriptan, eplerenone, everolimus, felodipine, indinavir, fluticasone, lopinavir, lovastatin, lurasidone, maraviroc, midazolam, nisoldipine, quetiapine, saquinavir, sildenafil, simvastatin, sirolimus, tolvaptan, tipranavir, triazolam, ticagrelor, and vardenafil

■ **Traditional Chinese medicines with known anti-tumor indications (patients should discontinue the use of the following drugs for at least 14 days before starting the investigational medicinal product; other traditional Chinese medicines with anti-tumor indications should also be prohibited and washed out for at least 14 days; traditional Chinese medicines without anti-tumor indications are not recommended)**

Cinobufotalin Tablets, Cinobufotalin Injection, Pingxiao Tablets (Capsules), Aidi Injection, Antike Capsules, Shenlian Capsules (Granules), Cidan Capsules, Compound Banmao Capsules, Compound Taxus Capsules, Compound Kushen Injection, Ganfule Tablets (Capsules), Huazheng Huisheng Oral Liquid, Huisheng Oral Liquid, Jinlong Capsules, Kanglaite Soft Capsules, Kanglaite Injection, Weimaining Capsules, Xiaoaiping Pills (Tablets, Capsules, Granules, Oral Liquid), Tongguanteng Injection (Xiaoaiping Injection), Brucea Javanica Oil Emulsion Injection, Brucea Javanica Oil Soft Capsules (Oral Emulsion), and Zilongjin Tablets

Appendix F. QTc Fridericia's Formula

QTcF = QT/(RR^0.33)

Note: QT refers to the interval between the beginning of the Q wave and the end of the T wave; RR refers to the mean interval from the R wave in the QRS complex to the next R wave (obtained by dividing 60 s by heart rate).

Appendix G. Creatinine Clearance Formula (Cockcroft-Gault Formula)

$$\frac{(140 - \text{Age}) \times \text{Body weight (kg)}}{72 \times \text{Serum creatinine (mg/dL)}} \text{ (Female} \times 0.85)$$

Note: Creatinine clearance is expressed in mL/min; age is in years; for males, use the unadjusted value; for females, multiply the result by 0.85.

Appendix H. Surgical Grading in *Administrative Measures for the Application of Medical Technology* (2018 Edition)

Surgical Grading in *Administrative Measures for the Application of Medical Technology*

**Grade**	**Definition**
Grade I surgery	Common surgery with relatively low risk, simple procedure, and low technical difficulty
Grade II surgery	Surgery with certain risk, general process complexity, and some technical difficulties
Grade III surgery	Surgery with relatively high risk, relatively complex process, and difficult procedure
Grade IV surgery	Major surgery with high risk, complex process, and significant difficulty

Appendix I Distribution of Proliferating Bone Marrow in Adult Cancer Patients

Distribution of proliferating bone marrow in adult cancer patients determined using FLT-PET imaging[9]

Site	Percentage of total red bone marrow
Skull	2.9
Cervical spine	4.3
Scapulae	3.8
Sternum	2.9
Thoracic spine	19.9
Proximal humeri	1.9
Ribs and clavicles	8.8
Lumbar spine	16.6
Sacrum	9.2
Pelvis	25.3
Proximal	4.5
