# Supplementary material for: High-Dose Aumolertinib for Untreated EGFR-Variant Non–Small Cell Lung Cancer With Brain Metastases: The ACHIEVE Phase 2 Nonrandomized Clinical Trial
Source: JAMA Oncol. 2025 Jun 26;11(8):900–8. doi: 10.1001/jamaoncol.2025.1779 (PMC12203398; doi:10.1001/jamaoncol.2025.1779)
Supplement: Supplement 2. — eTable 1. Treatment-related adverse events eTable 2. Post-hoc univariate analysis of progression-free survival eTable 3. Procedure of backward selection in multivariate Cox regression model eTable 4. Post-hoc multivariate Cox regression analysis of progression-free survival among patients with EGFR variants in baseline plasma ctDNA eFigure 1. Genomic landscape in patients with available baseline plasma samples for circulating tumor DNA sequencing eFigure 2. Association between EGFR variant clearance in plasma circulating tumor DNA at day 1 of cycle 2 and progression-free survival among patients with EGFR variants in baseline plasma circulating tumor DNA eFigure 3. Schoenfeld residual scaled plots in multiple univariate Cox regression models eFigure 4. Schoenfeld residual scaled plots in multivariate Cox regression model [file jamaoncol-e251779-s002.pdf]

Supplemental Online Content

Li H, Chen K, Gong L, et al. High-dose aumolertinib for untreated *EGFR*-variant non–small cell lung cancer with brain metastases: the ACHIEVE phase 2 nonrandomized clinical trial. *JAMA Oncol.* Published online June 26, 2025. doi:10.1001/jamaoncol.2025.1779

eTable 1. Treatment-related adverse events

eTable 2. Post-hoc univariate analysis of progression-free survival

eTable 3. Procedure of backward selection in multivariate Cox regression model

eTable 4. Post-hoc multivariate Cox regression analysis of progression-free survival among patients with EGFR mutations in baseline plasma ctDNA

eFigure 1. Genomic landscape in patients with available baseline plasma samples for circulating tumor DNA sequencing

eFigure 2. Association between EGFR mutation clearance in plasma circulating tumor DNA at day 1 of cycle 2 and progression-free survival among patients with EGFR mutations in baseline plasma circulating tumor DNA

eFigure 3. Schoenfeld residual scaled plots in multiple univariate Cox regression models

eFigure 4. Schoenfeld residual scaled plots in multivariate Cox regression model

This supplemental material has been provided by the authors to give readers additional information about their work.

© 2025 Li H et al. *JAMA Oncology*.

eTable 1. Treatment-related adverse events (N=63)[a]

	No. (%)	
**Event**	**Any grade**	**Grade ≥3**
Any event	58 (92.1)	20 (31.7)
Blood creatine phosphokinase increased	43 (68.3)	17 (27.0)
Aspartate aminotransferase increased	31 (49.2)	0
Alanine aminotransferase increased	24 (38.1)	2 (3.2)
Blood lactate dehydrogenase increased	17 (27.0)	0
White blood cell count decreased	12 (19.0)	0
Anemia	10 (15.9)	1 (1.6)
Rash	10 (15.9)	0
Platelet count decreased	9 (14.3)	0
Myalgia	7 (11.1)	0
Amylase increased	1 (1.6)	1 (1.6)
Conjunctivitis	1 (1.6)	1 (1.6)

[a]Any grade treatment-related adverse events occurred in ≥10% of patients and all grade ≥3 treatment-related adverse events were reported.

© 2025 Li H et al. *JAMA Oncology*.

eTable 2. Post-hoc univariate analysis of progression-free survival

	Univariate Cox model		
**Variable**	**HR (95% CI)**	**Unadjusted *P*-value**	**FDR Adjusted *P*-value[a]**
Age (years, n=63)	1.09 (0.93-1.27)	.29	.50
Time-varying age[b]	0.97 (0.91-1.03)	.31	-
Sex (female vs. male, n=63)	0.91 (0.49-1.70)	.78	.78
ECOG performance status (0 vs. 1, n=63)	0.44 (0.19-0.99)	.047	.28
Smoking status (never smokers vs. current or former smokers, n=63)	0.84 (0.42-1.69)	.63	.77
Clinical stage (IVA vs. IVB, n=63)	0.64 (0.31-1.35)	.24	.49
EGFR mutation type (exon 19 deletion vs. exon 21 L858R, n=63)	2.54 (0.32-20.8)	.39	.58
Time-varying EGFR mutation type[b]	0.43 (0.18-1.00)	.050	-
Symptoms of brain metastases (no vs. yes, n=63)	0.20 (0.02-1.87)	.16	.48
Time-varying symptom of brain metastases[b]	1.75 (0.70-4.40)	.23	-
Extracranial metastases (no vs. yes, n=63)	0.90 (0.46-1.75)	.76	.78
Number of brain metastases (1-3 vs. >3, n=63)	1.60 (0.85-3.00)	.14	.48
EGFR mutations in baseline plasma ctDNA (yes vs. no, n=60)	1.19 (0.57-2.51)	.65	.77
TP53 co-mutation (no vs. yes, n=45)	0.63 (0.29-1.36)	.24	.49
EGFR mutation clearance in plasma ctDNA at C2D1 (yes vs. no, n=45)	0.17 (0.07-0.42)	<.001	.002

Abbreviations: C2D1, day 1 of cycle 2; ctDNA, circulating tumor DNA; ECOG, Eastern Cooperative Oncology Group; EGFR, epidermal growth factor receptor; FDR, false discovery rate; HR, hazard ratio.

[a]FDR for multiple testing adjusted by Benjamini-Hochberg method.

[b]Three time-varying covariates (age×log[time], EGFR mutation type×log[time], and symptom of brain metastases×log[time]) were included in univariate Cox regression model based on proportional hazards assumption tested by examining Schoenfeld residuals.

© 2025 Li H et al. *JAMA Oncology*.

eTable 3. Procedure of backward selection in multivariate Cox regression model

Summary of backward elimination					
**Step**	**Effect removed**	**DF**	**Wald Chi-square**	**Pr > ChiSq**	**AIC**
1	Age	1	0.0348	0.8521	189.467
2	Extracranial metastases	1	0.1134	0.7364	187.502
3	Number of brain metastases	1	0.1227	0.7261	183.740

© 2025 Li H et al. *JAMA Oncology*.

eTable 4. Post-hoc multivariate Cox regression analysis of progression-free survival among patients with EGFR mutations in baseline plasma ctDNA (N=45)

	Multivariate Cox model	
**Variable**	**HR (95% CI)**	***P*-value**
Age (years)[a]		
Sex (male vs. female)	1.53 (0.44-5.39)	.50
ECOG performance status (0 vs. 1)	0.49 (0.16-1.52)	.22
Smoking status (never smokers vs. current or former smokers)	0.18 (0.04-0.77)	.021
Clinical stage (IVA vs. IVB)	0.78 (0.25-2.45)	.68
EGFR mutation type (exon 19 deletion vs. exon 21 L858R)	2.24 (0.19-26.58)	.52
Time-varying EGFR mutation type[b]	0.39 (0.14-1.08)	.070
Symptoms of brain metastases (no vs. yes)	0.14 (0.04-0.51)	.003
Extracranial metastases (no vs. yes)[a]		
Number of brain metastases (1-3 vs. >3)[a]		
TP53 co-mutation (no vs. yes)	1.16 (0.06-23.62)	.92
Time-varying TP53 co-mutation[b]	1.21 (0.39-3.72)	.74
EGFR mutation clearance in plasma ctDNA at C2D1 (yes vs. no)	0.14 (0.04-0.47)	.001

Abbreviations: C2D1, day 1 of cycle 2; ctDNA, circulating tumor DNA; ECOG, Eastern Cooperative Oncology Group; EGFR, epidermal growth factor receptor; HR, hazard ratio.

[a]Three variables were excluded from model in backward stepwise selection procedure with a significant level 0.5.

[b]Two time-varying covariates (EGFR mutation type×log[time] and TP53 co-mutation×log[time]) were included in multivariate Cox regression model based on proportional hazards assumption tested by examining Schoenfeld residuals.

© 2025 Li H et al. *JAMA Oncology*.

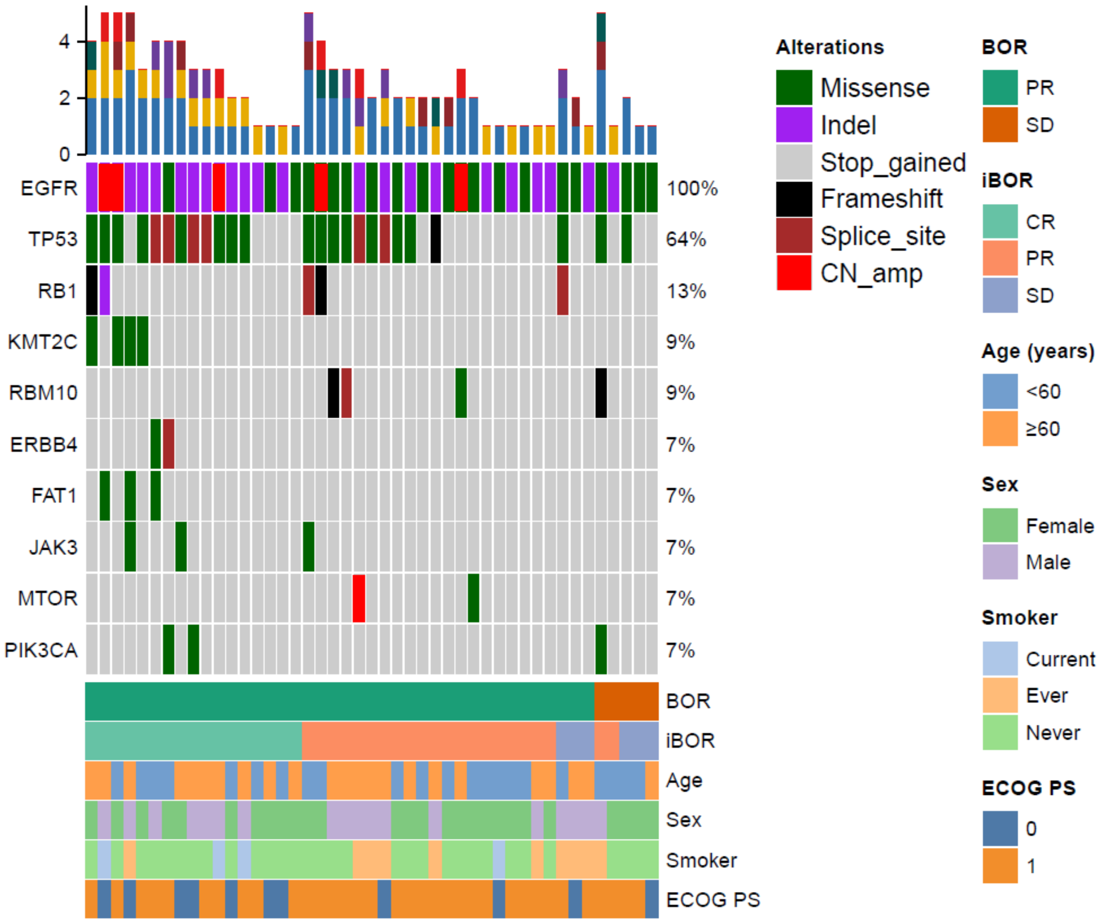

eFigure 1. Genomic landscape in patients with available baseline plasma samples for circulating tumor DNA sequencing (N=60). BOR, best overall response; CR, complete response; ECOG PS, Eastern Cooperative Oncology Group performance status; iBOR, intracranial best overall response; PR, partial response; SD, stable disease.

© 2025 Li H et al. *JAMA Oncology*.

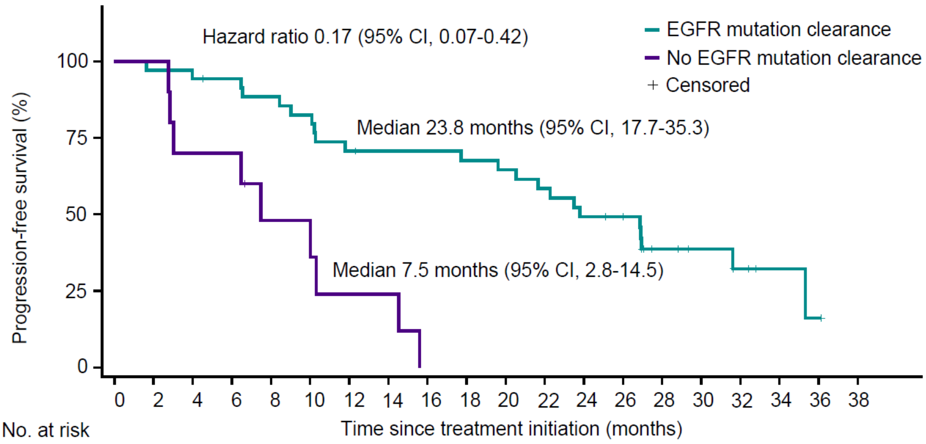

No. at risk (censored)	0	2	4	6	8	10	12	14	16	18	20	22	24	26	28	30	32	34	36	38
EGFR mutation clearance	35 (0)	34 (0)	33 (0)	32 (1)	30 (1)	28 (1)	24 (1)	23 (2)	23 (2)	22 (2)	21 (2)	19 (2)	16 (2)	15 (3)	8 (7)	6 (9)	4 (10)	2 (12)	1 (12)	0 (13)
No EGFR mutation clearance	10 (0)	10 (0)	7 (0)	7 (0)	4 (1)	4 (1)	2 (1)	2 (1)	0 (1)	0 (1)	0 (1)	0 (1)	0 (1)	0 (1)	0 (1)	0 (1)	0 (1)	0 (1)	0 (1)	0 (1)

eFigure 2. Association between EGFR mutation clearance in plasma circulating tumor DNA at day 1 of cycle 2 and progression-free survival among patients with EGFR mutations in baseline plasma circulating tumor DNA (N=45). EGFR, epidermal growth factor receptor.

© 2025 Li H et al. *JAMA Oncology*.

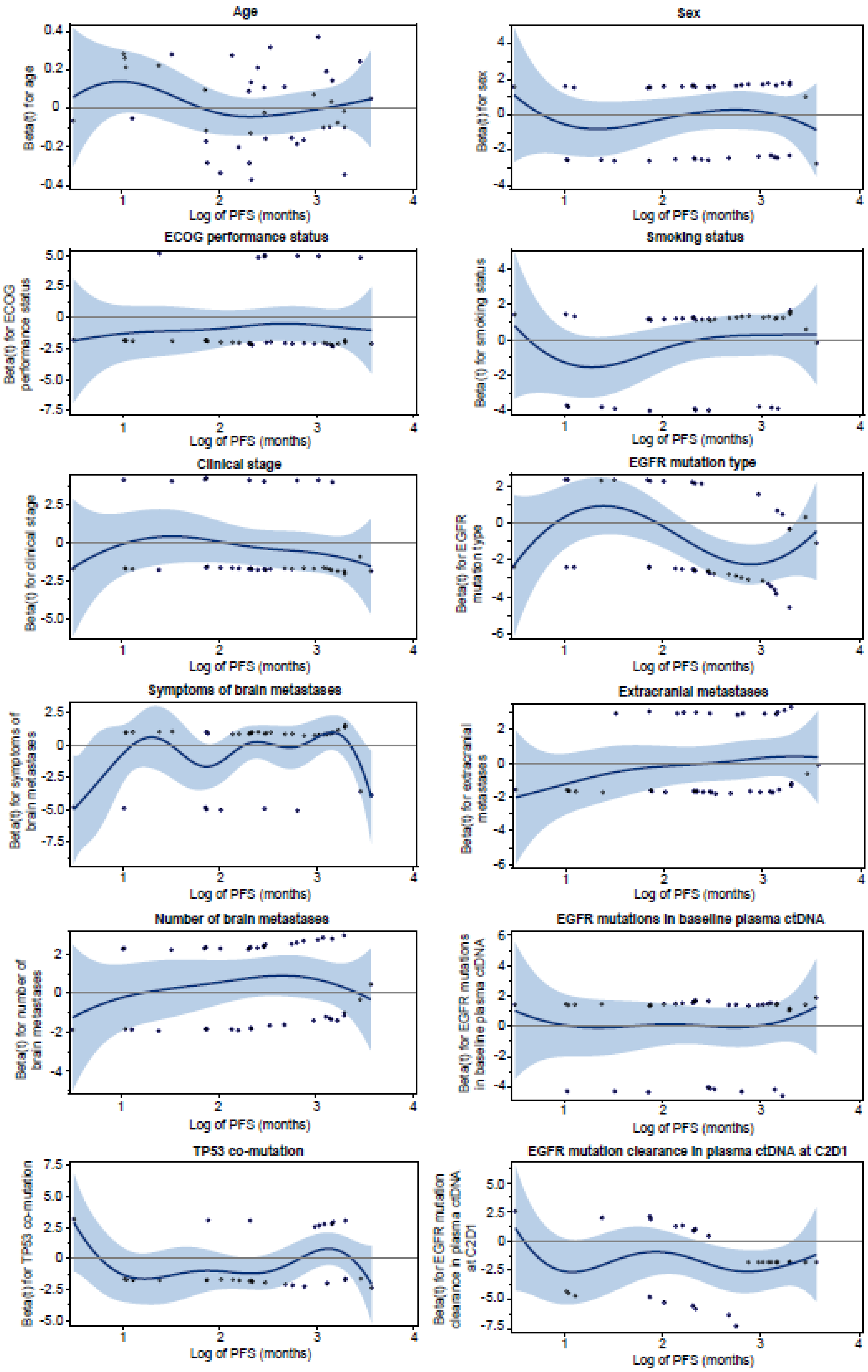

eFigure 3. Schoenfeld residual scaled plots in multiple univariate Cox regression models.

© 2025 Li H et al. *JAMA Oncology*.

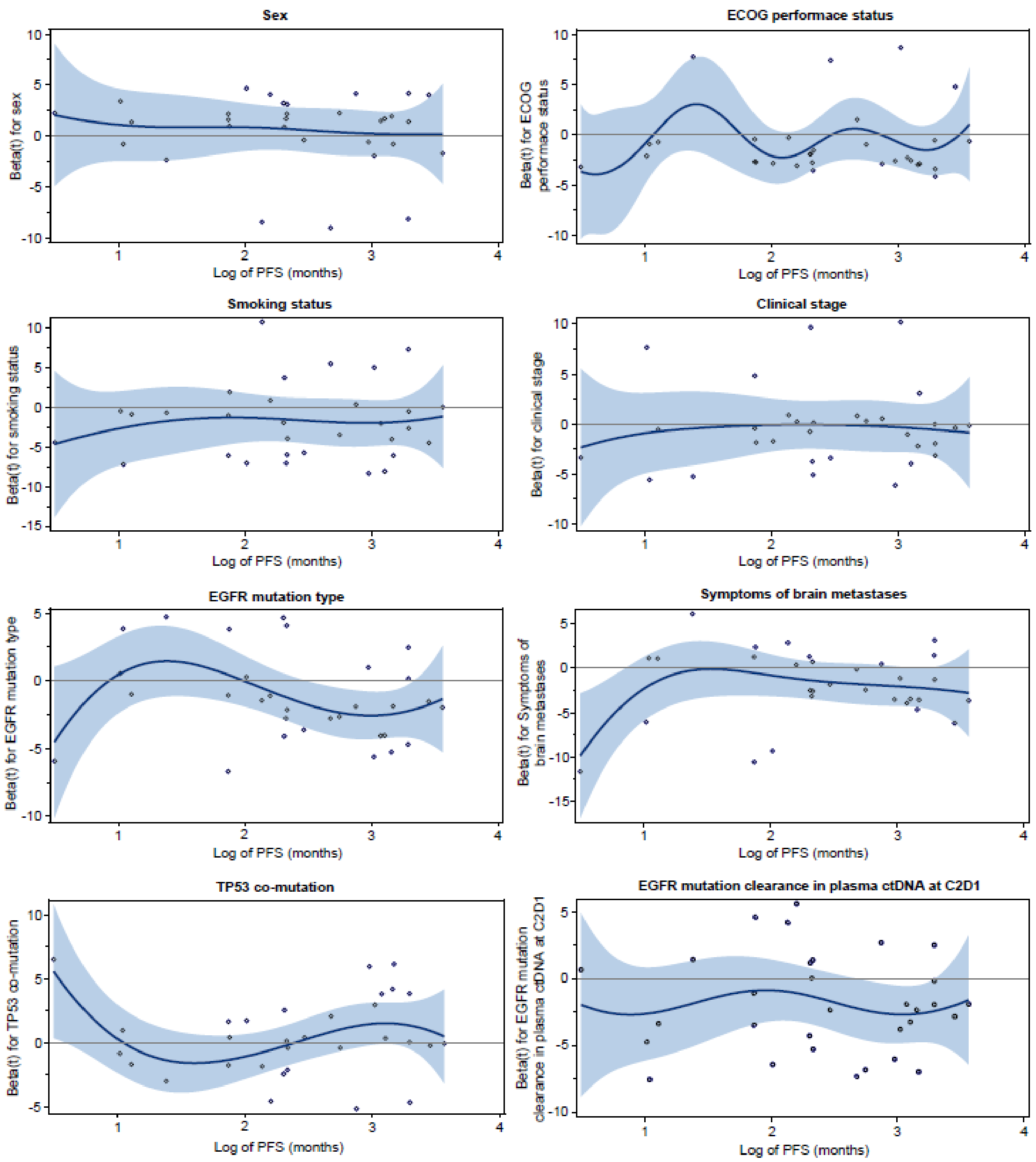

eFigure 4. Schoenfeld residual scaled plots in multivariate Cox regression model.

© 2025 Li H et al. *JAMA Oncology*.
