# Supplementary material for: High-Dose Aumolertinib for Untreated EGFR-Variant Non–Small Cell Lung Cancer With Brain Metastases: The ACHIEVE Phase 2 Nonrandomized Clinical Trial
Source: JAMA Oncol. 2025 Jun 26;11(8):900–8. doi: 10.1001/jamaoncol.2025.1779 (PMC12203398; doi:10.1001/jamaoncol.2025.1779)
Supplement: Supplement 3. — Data Sharing Statement [file jamaoncol-e251779-s003.pdf]

Data Sharing Statement

Li. High-Dose Aumolertinib for Untreated EGFR-Variant Non–Small Cell Lung Cancer With Brain Metastases. *JAMA Oncol*. Published June 26, 2025. doi:10.1001/jamaoncol.2025.1779

Data

Additional Information: Name of the trial registry:Almonertinib as First-line Treatment in Patients With EGFR Mutations Positive in Advanced NSCLC With Brain Metastases (ACHIEVE) registry's URL: https://clinicaltrials.gov/study/NCT04808752?term=NCT04808752&rank=1 the trial registration number：NCT04808752
Data available: Yes
Data types: Deidentified participant data
How to access data: fanyun@zjcc.org.cn
When available: With publication

Supporting Documents

Document types: None

Additional Information

Who can access the data: researchers whose proposed use of the data has been approved
Types of analyses: Any
Mechanisms of data availability: After approval of proposal
